# BACH1 stabilization by antioxidants facilitates hepatocellular carcinoma metastasis

**DOI:** 10.1038/s41419-026-08865-0

**Published:** 2026-06-09

**Authors:** Qiqi Bu, Zhanhang Wang, Qing Wang, Xiaojing Qi, Shanshan Lin, Shujuan Liu, Yiguo Zhang

**Affiliations:** 1 The Laboratory of Cell Biochemistry and Topogenetic Regulation, College of Bioengineering & Chongqing Academy of Medical Sciences, Chongqing General Hospital, Faculty of Medicine, Chongqing University, Chongqing, 400044 China; 2 College of Animal Science and Technology, Northwest A&F University, Yangling, 712100 China; 3 Yu-Yue Pathology Scientific Research Center, 313 Gaoteng Avenue, Jiulongpo District, Chongqing, 400039 China; 4 Department of Laboratory Medicine, Chongqing Center for Clinical Laboratory, Chongqing Academy of Medical Sciences, Chongqing General Hospital, School of Medicine, Chongqing University, Chongqing, 401147 China

**Keywords:** Cell invasion, Metastasis

## Abstract

Reactive oxygen species (ROS) plays certain contradictory and complex roles in the carcinogenesis and progression of cancers. In such process, antioxidants enable to promote the malignant invasion and migration of hepatocellular carcinoma (HCC) cells, but the underlying mechanisms remain elusive. Here, we found that two antioxidants, glutathione (GSH) and N-acetylcysteine (NAC), stabilized BACH1 (i.e., BTB and CNC homology 1) by decreasing ROS levels, thus facilitated BACH1-dependent migration of HCC cells in vitro and their metastasis in vivo. Mechanistically, knockout of BACH1 mildly alleviated endoplasmic reticulum stress/adaptive unfolded protein response (UPR) and repressed epithelial-mesenchymal transition (EMT) to inhibit cell migration. Further examinations revealed that BACH1 binds to consensus ARE sites in the promoter regions of key genes responsible for glycolytic pathway to activate their transcription, while concurrently repressing the expression of mitochondrial respiratory chain genes. This dual regulation thereby promoted the Warburg effect and facilitated HCC cell migration driven by glycolysis. The BACH1-regulated metabolic reprogramming was further unraveled by non-targeted metabolomics. Collectively, these demonstrate that the redox-sensitive transcription factor BACH1 functions as a crucial metastasis-promoter of HCC, and thus antioxidants stimulate BACH1-dependent HCC metastasis.

## Introduction

Liver cancer is a malignant tumor with considerable morbidity and mortality globally, posing significant public health challenges [1]. Hepatocellular carcinoma (HCC), is the most prevalent type of liver cancer and the leading reason for cancer-related death. Despite significant advances in treatments, the prognosis for patients remains unsatisfactory, which is inseparable from the high metastasis rates of HCC [2, 3]. In such context, it is essential to understand the underlying mechanisms of HCC metastasis as well as molecular targets that can be applied for therapy.

Tumor cells generally produce greater levels of reactive oxygen species (ROS) than normal cells [4]. Over the past decades, the perspective that ROS stimulates tumorigenesis and development has dominated. Relevant studies have shown that mitochondrial ROS contribute normal cells initiate nuclear or mitochondrial DNA mutations and then neoplastic transformation [5]. Moreover, ROS signaling promotes proliferation and survival of tumor cells in many cancers [6, 7]. This has prompted many people to supplement their diets with antioxidants in the hope of preventing or even treating cancer. However, with the further development of scientific research, many clinical trials have arrived at an opposite conclusion, that ROS limits tumor progression while antioxidants can help [8–11]. For example, oxidative stress can inhibit melanoma cell metastasis while administering antioxidants in drinking water accelerates tumor metastasis in the mice model of melanoma [8–10]. Similarly, supplementing with the antioxidants N-acetylcysteine (NAC) or vitamin E promotes KRAS-induced lung cancer metastasis [11]. In HCC, high levels of ROS cause methylation of E-cadherin promoter, repressing its expression and enhancing epithelial-mesenchymal transition (EMT) [12]. Conversely, antioxidant supplements promote HCC formation and tumor growths by reducing intracellular ROS [13]. The contradictory roles of ROS in cancer hinder a clear comprehension of redox regulation. Elucidating these mechanisms remains an important goal for future research [14].

In absence of exogenous antioxidant intervention, tumor cells maintain redox balance by expressing the endogenous antioxidative genes, most of which are modulated by nuclear factor erythroid 2-related factor 2 (NRF2) [15]. Like NRF2, the redox-sensitive transcription factor BTB and CNC homology 1 (BACH1) is also a major player in cap’n’collar and basic region-leucine zipper (CNC-bZIP) family, which performs an essential role in the maintenance of heme homeostasis and regulation of antioxidant genes transcription [16, 17]. Under oxidative stress conditions, ROS oxidizes heme-containing proteins to release free heme, promoting the BACH1 degradation by the proteasome [18, 19]. NRF2 accumulates and forms functional heterodimers with Mafs for binding to antioxidant response elements (*ARE*s) in the promoter domains of antioxidative genes, activating their transcriptional expression [20]. BACH1 competes with NRF2 for *AREs* and acts as a transcriptional suppressor of antioxidative genes, including heme oxygenase 1 (HO-1) [21]. Of note, BACH1 promotes invasion and migration in a variety of cancer cells by transcriptionally activating critical metastasis-related genes [22–25]. Antioxidant treatment or NRF2 activation drives lung cancer metastasis by reducing ROS levels to stabilize BACH1, thereby enhancing the transcription of key glycolytic enzyme genes [11]. Previous studies have shown that antioxidants promote the migration and invasion of HCC cells [13]. However, whether this pro-metastatic effect is mediated by BACH1, as well as the precise regulatory mechanisms of BACH1 in HCC metastasis, remain poorly understood.

To address these questions, we demonstrated that the antioxidants NAC and glutathione (GSH) enhanced HCC cell metastasis in vivo using mouse models. BACH1 knockout suppressed metastasis and abolished the pro-metastatic effect of antioxidants in HCC cells in vivo. The in vitro experiments showed that antioxidants stabilized BACH1 through decreasing ROS and free heme levels, promoted BACH1-dependent HCC cell migration and invasion. Mechanistically, knockout of BACH1 mildly alleviated endoplasmic reticulum stress/ adaptive unfolded protein response (UPR) and repressed EMT to inhibit cell migration. For another, BACH1 activated transcription of key genes in glycolytic pathway and negatively regulated expression of mitochondrial respiratory chain genes, thus facilitating the Warburg effect and glycolysis-mediated HCC cell migration. In the end, non-targeted metabolomics revealed that BACH1 regulated metabolic reprogramming of HCC cells. Taken together, BACH1 stabilization by antioxidants facilitated HCC metastasis. Our studies provide novel insights into potential therapeutic targets for metastasis of HCC.

## Methods

### Cell lines culture, transfection and chemical treatment

Human hepatocellular carcinoma (HepG2) cell line was originally obtained from Zhong Yuan Ltd. (Beijing, China). Three other cell lines, HepG2 BACH1-KO (knockout mutant), Lenti-BACH1 (stably overexpressing BACH1) or Lenti-Control were all established from HepG2 cells. We also acquired the HCCLM3 cell line from the Living Cancer Institute of Fudan University, China, and created another HCCLM3 BACH1-KO (knockout mutant) cell line from it. These cell lines were constructed as we described in detail previously [26]. Additionally, we reconstituted BACH1 expression in the HepG2 BACH1-KO cells by infecting them with a lentivirus carrying the human BACH1 cDNA (lenti-BACH1). A control cell line was established in parallel by infecting HepG2 BACH1-KO cells with a control lentivirus (lenti-Control). Following infection, cells were selected with hygromycin B (200 μg/mL) for 7 days to establish stable cell lines, designated as HepG2 BACH1-KO + Lenti-BACH1 and HepG2 BACH1-KO + Lenti-Control, respectively. Successful reconstitution of BACH1 expression was confirmed by Western blot analysis.

All cell lines were cultured in DMEM (Gibco, USA) supplemented with 100 units/mL streptomycin and penicillin, 10% fetal bovine serum (FBS) at 37°C and 5% CO_2_ concentration, and performed according to the ATCC guidelines. An expression plasmid for human BACH1 was constructed to clone its coding sequence into the pcDNA3.1 vector, and cells were transfected by incubation with Lipofectamine 3000 (Invitrogen, USA). Cells were treated with the following chemicals, N-Acetyl-L-cysteine (NAC, A7250, Sigma-Aldrich), L-Glutathione reduced (GSH, V900456, Sigma-Aldrich), Cycloheximide (HY-12320, MCE, Shanghai, China), Hemin (51280, Sigma-Aldrich), UK-5099 (HY-15475, MCE, Shanghai, China), Etomoxir (HY-50202, MCE, Shanghai, China) and 2-Deoxy-D-glucose (2-DG, HY-13966, MCE, Shanghai, China).

### Animal experiments

Wild-type HCCLM3 and HCCLM3 BACH1-KO cell lines at exponential growth phase (1×10^6^) were resuspended in 0.2 mL PBS solution and injected into the tail veins of 5-week old male nude mice to establish metastasis model in vivo (*n* = 15 per cell line). Subsequently, mice injected with the same cells were randomly divided into three groups of five and given distilled water, NAC (150 mg/kg) or GSH (100 mg/kg) respectively. After 6 weeks, all mice were sacrificed and their lungs were dissected. To count the number of metastatic nodules in the lungs. Lungs were embedded in paraffin for hematoxylin-eosin staining and Immunohistochemistry (IHC). Animal experiments employed randomization and blinding.

### Immunohistochemistry (IHC)

Briefly, fixed mice tissues were embedded in paraffin and produced as 5 μm sections. The detailed procedure is described in the Supplementary file.

### Cell migration and invasion assays

Transwell chambers with 8-μm-pore were designed to assay the ability to invasion and migration of HCC cells. For the migration assay, experimental cells (5×10^4^) were suspended in 200 μL serum free DMEM and seeded in the upper chambers. Adding 450 μL of DMEM supplemented with 20% FBS to the lower chambers, and cells were then incubated at 37°C in an incubator with 5% CO_2_ for 24 h.

For invasion assay, we precoated the mixture of matrigel (Solarbio, Beijing, China) and DMEM to the upper chamber membrane about 2 h in 37°C. Subsequently, experimental cells (5×10^4^) were suspended in 200 μL serum free DMEM and seeded in the upper chambers. Adding 450 µl of DMEM supplemented with 20% FBS to the lower chambers, and cells were incubated in the 37°C incubator with 5% CO_2_ for 24 h. Then cells were fixed with paraformaldehyde for 30 min and stained with crystalline violet staining solution for 20 min. After removing non-migratory/invasive cells from the upper surface of filter membrane, the stained cells were counted in five randomized fields using a light microscope.

### Intracellular ROS and GSH content measurement

The intracellular ROS was measured with ROS assay kit (Solarbio, Beijing, China). In brief, experimental cells were seeded in 6-well plates and treated with NAC or GSH for 24 h, respectively. Subsequently, the cells were incubated with a 10 μM DHE probe for 40 min at 37°C. After the cells were washed with serum free culture medium three times, the fluorescent intensity was measured with a fluorescence microscope in 610 nm emission and 518 nm excitation lights.

The experimental cells were seeded in 6-well plates and treated with GSH or NAC for 24 h, respectively. GSSG and GSH assay kit (Beyotime, Shanghai, China) was used to measure intracellular total GSH content based on the manufacturer’s instructions.

### Free heme measurement

HepG2 and HCCLM3 cells were treated with NAC or GSH for 24 h, respectively. Subsequently, Neutral acetone was used to extract free heme in cells, as described [27]. Heme concentration was quantified following the instruction of the Heme assay kit (MAK316, Sigma-Aldrich) and normalized using the protein concentration in each sample.

### Real-time quantitative PCR

The total RNA of the cells was extracted with an RNA kit (Tiangen, Beijing, China), for more details see the Supplementary file. The primer pairs used for real-time quantitative PCR were listed in Table S1.

### Western blotting

The experimental cell proteins were extracted with RIPA buffer (Solarbio, Beijing, China) according to the procedure described in the Supplementary file. Primary antibodies used in the immunoblotting process including BACH1 (sc-271211, Santa Cruz Biotechnology), NRF2 (ab62352), GSR (ab124995), GCLC (ab207777), NQO1 (ab80588), GCLM (ab126704), HO-1 (ab52947), GPX1 (ab108427), XBP1 (ab109221), IRE1 (ab37073), eIF2A (ab169528), PERK (ab229912), SNAL1 (ab216347), SNAIL2 (ab302780), NDUFC2 (ab192265) (all these antibodies purchased from Abcam), HK1 (D221854), PFKL (D222865), SDHB (D262175) (these antibodies purchased from Sangon Biotechnology, China), SOD1 (AF3418, R&D Systems, USA), G6PD (A1537), TXN2 (A12591), p-PERK (AP1501), GLUT4 (A7637), LDHA(A1146), GLUT1 (A11208), MTCO1 (A17889), MTCO2 (A11913) (all eight antibodies purchased from ABclonal, Wuhan, China), FGF21 (A5710, bimake, USA), HSP60 (A5629, bimake, USA), GRP75 (A5420, bimake, USA), CDH1(20874-1-AP, Proteintech Group), CDH2 (22018-1-AP, Proteintech Group), Vimentin (60330-1-Ig, Proteintech Group), GAPDH (#2118, Cell Signaling Technology), HK2 (#2867, Cell Signaling Technology), β-actin (TA-09, from ZSGB-BIO, Beijing, China) and GLUT3 (bs-22374R, BIOSS, Beijing, China). Finally, the protein bands were detected using an ECL illumination system and gray-scale calculations were performed using ImageJ software.

### Pyruvate and lactate content measurement

Both pyruvate and lactate contents were measured using their respective assay kits (BC2200, BC2230, Solarbio, China) following the manufacturer’s instructions. Pyruvate and lactate concentrations were calculated from standard curves and normalized using the protein concentrations. Enhanced BCA protein assay kit (P0010, Beyotime, Shanghai, China) was used to measure the protein concentration in each sample.

### ATP measurement

Experimental cells were seeded into 6-well plates and lysed in the lysis buffer (200 μL/well) at 4°C. Subsequently, cell lysates were centrifuged at 4°C, 12,000 g for 5 min and the supernatant was transferred to a clean centrifuge tube. Intracellular ATP levels were measured according to the instruction of the ATP assay kit (Beyotime, Shanghai, China). The concentrations of ATP were calculated from the standard curve and normalized using the protein concentrations in each sample. All samples were normalized based on cell count and subjected to three independent replicate experiments.

### CHIP-seq analysis

Briefly, cell samples were crosslinked with formaldehyde and subsequently terminated with glycine. Subsequently, ultrasonic or enzymatic digestion fragments the chromatin (primarily into 200–500 base pairs). According to the manufacturer’s instructions, add anti-BACH1 antibody to capture the DNA-protein complex. DNA was purified using the DNA Clean & Concentrator-5 Kit and sequenced on an Illumina HiSeqTM 2000 platform.

### Luciferase reporter assay

Those *AREs* and its neighboring sequences included in the HK1, GAPDH, PFKL, or GLUT4 promoters were inserted into pGL3 promoter vectors to create ARE reporter gene plasmids. To generate a mutant version lacking the predicted BACH1 binding site, site-directed mutagenesis was performed on the relevant *ARE* reporter plasmids (HK1-LUC-#1, GAPDH-LUC-#6 and GLUT4-LUC-#11) using a Site-Directed Mutagenesis Kit (Beyotime Biotechnology, Shanghai, China). More steps in the Supplementary file.

### Metabolomics analysis

HepG2, HepG2 BACH1-KO, Lenti-BACH1 and Lenti-Control cells (5×10^6^) were seeded in 10 cm cell-culture dish, each experimental cells were repeated six times (*n* = 6). Prior to the start of experiments, cells were maintained in SFM (Gibco, 10639011) for at least two passages to ensure metabolic steady state. When cell density reached 80%, they were washed three times with pre-chilled PBS, and metabolic activity was inhibited by adding pre-chilled 80% methanol. After freezing in liquid nitrogen for 15 min, cells were stored at -80°C immediately. Further metabolite extraction and UPLC-MS analysis were performed by Genomics Institute (Beijing, China). For data analysis of non-targeted metabolomics, raw data was collected for metabolite peak extraction and identification using Compound Discoverer 3.4 software (Thermo Fisher Scientific, USA). The result file was inputted to MetaX [28] for data preprocessing and next step analysis. The Probabilistic Quotient Normalization (PQN) [29] method was used to normalize the data.

### Statistical analysis

The statistical analyses were conducted using the GraphPad Prism 9.1 and SPSS 23.0 tools. All successfully tested animals and samples were included in our analysis. The data were subjected to at least three independent experiments and are expressed as (mean ± SD). Statistical differences between the experimental groups and within the groups were calculated using one-way ANOVA. The *p*-value ≤ 0.05 was regarded statistically significant.

## Results

### Antioxidants NAC and GSH stimulate BACH1-dependent HCC cell metastasis

To elucidate the role of BACH1 in antioxidant-promoted HCC cell metastasis and further explore its potential mechanisms in regulating HCC metastasis. We used a lentiviral system to establish stable BACH1-overexpressing and control cell lines in HepG2 cells, named Lenti-BACH1 and Lenti-Control, respectively. Furthermore, cell lines with BACH1 knockout were established in HCCLM3 and HepG2 cells through gene editing technology (CRISPR/Cas9), and were named HCCLM3 BACH1-KO and HepG2 BACH1-KO. These cell lines were constructed as we previously described in detail [26] and re-validated via Western blotting in this study (Fig. S1A–C). To further confirm antibody specificity, we performed additional Western blotting. As shown in Fig. S1D, BACH1 protein was undetectable in HepG2 BACH1-KO cells, while Fig. S1E confirmed the absence of NRF2 protein in NRF2-knockout HepG2 cells. BACH1 is renowned for its repression of the transcription of antioxidant genes. We found that knockout of BACH1 in HCCLM3 and HepG2 cells resulted in a significant upregulation of mRNA and protein levels of abundant antioxidant genes, further validating the successful knockout of BACH1 (Fig. S1F–K). Conversely, enforced BACH1 overexpression in HepG2 cells (Lenti-BACH1) was accompanied by a marked reduction in both mRNA and protein levels of antioxidant genes (Fig. S1L–N). Furthermore, subcellular localization analysis confirmed that the ectopically expressed BACH1 protein was predominantly localized in the nucleus (Fig. S1O). These results collectively demonstrate that the overexpressed BACH1 is transcriptionally active in our experimental system.

**Fig. 1:**
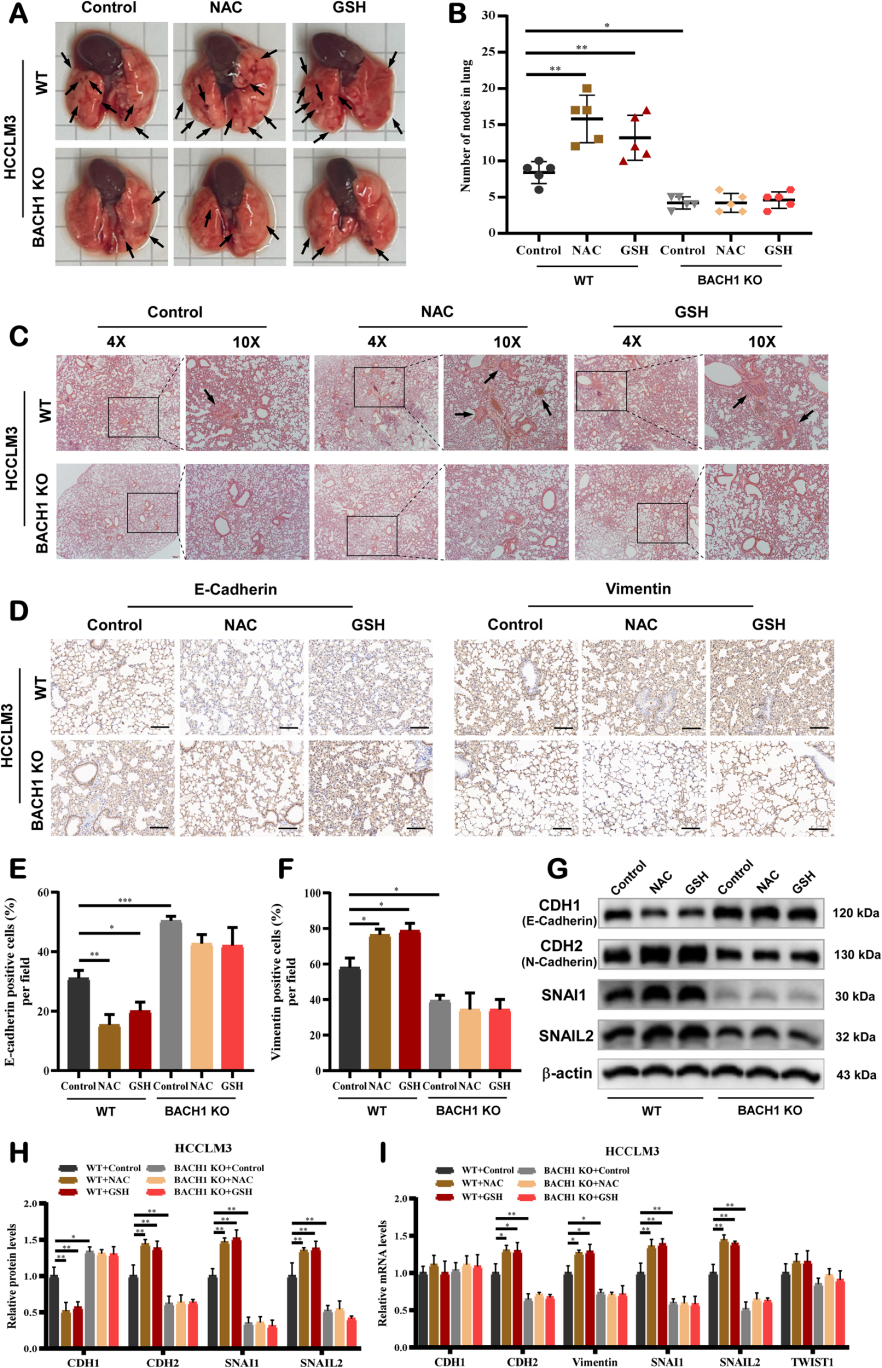
Antioxidants NAC and GSH stimulate BACH1-dependent HCC cell metastasis in vivo. **A** Representative images of lung histological lesions in control, NAC and GSH-treated mice after injection of HCCLM3 and HCCLM3 BACH1-KO cells into nude mice tail vein. Black arrows indicating visible lung metastatic nodules. **B** Quantification of lung metastatic nodules in each group (*n* = 5). All comparative experiments shown were performed concurrently in the same experimental batch. **C** Representative pictures of HE staining of lung tumors. Black arrows indicate metastatic tumor nodules. Scale bar = 200 µm in 4× images and 100 µm in 10× images. **D** IHC assay for E-cadherin and Vimentin protein in lung tissues of HCC cell metastasis model mice. Scale bar = 100 µm. **E, F** Quantification of E-cadherin and Vimentin protein expression in lung metastatic nodules detected by IHC (*n* = 5). **G, H** Detection and quantification of E-Cadherin (CDH1), N-Cadherin (CDH2), SNAI1 and SNAIL2 levels in HCCLM3 and HCCLM3 BACH1-KO cells treated with control, 5 mM NAC or 2 mM GSH for 24 h by Western blotting (*n* = 3). **I** RT-qPCR analysis of CDH1, CDH2, Vimentin, SNAI1, SNAIL2 and TWIST1 levels in HCCLM3 and HCCLM3 BACH1-KO cells treated with control, 5 mM NAC or 2 mM GSH for 24 h (*n* = 3 × 3). Data are presented as mean ± SD (*p < 0.05, **p < 0.01, ***p < 0.001).

Next, wild-type HCCLM3 and HCCLM3 BACH1-KO cells were injected into nude mice tail vein to establish in vivo metastasis models, followed by gavage administration of antioxidant NAC (150 mg/kg) or GSH (100 mg/kg), respectively. Metastasis was assessed based on the amount of lung nodules of mice after 6 weeks of treatment. Relevant results showed that in mice injected with wild-type HCCLM3 cells, the amount of lung metastatic nodules was markedly greater in NAC- and GSH-treated groups compared with the control group. However, NAC and GSH had no significant effect on lung metastasis in mice injected with HCCLM3 BACH1-KO cells (Fig. 1A, B). These indicated that GSH and NAC promoted HCC cells metastasis in vivo and this promotion was effectively reversed by the deletion of BACH1. Interestingly, lung metastatic nodules were obviously reduced in mice injected with HCCLM3 BACH1-KO cells compared to the placebo group injected with the wild-type of HCCLM3 cells (Fig. 1A, B), suggesting that knockout of BACH1 inhibited the metastasis of HCCLM3 cells in vivo. HE staining was consistent with the above results (Fig. 1C). Furthermore, immunohistochemical analysis showed that NAC and GSH treatments reduced the EMT marker E-cadherin level and upregulated the level of Vimentin in lung tissues of HCCLM3 cell metastasis model mice whereas the expressions of Vimentin and E-cadherin were not affected by NAC and GSH in lung tissues of HCCLM3 BACH1-KO cell metastasis model mice. BACH1 knockout led to an upregulation of E-cadherin and a downregulation of Vimentin in the lung tissues of metastasis model mice, respectively (Fig. 1D–F). To validate the EMT-related changes observed in vivo, we performed parallel experiments using cell extracts from cultures in vitro. As shown in Fig. 1G, H, NAC and GSH treatment downregulated the protein expression of epithelial marker CDH1 (E-cadherin) and upregulated the protein expression of mesenchymal marker CDH2 (N-cadherin) and EMT-related transcription factors SNAI1 and SNAIL2 in wild-type HCCLM3 cells, while these changes were largely abolished in HCCLM3 BACH1-KO cells. Knockout of BACH1 resulted in a significant increase in CDH1 protein level and a marked decrease in CDH2, SNAI1 and SNAIL2 protein levefls. q-PCR results further demonstrated that treatment with NAC and GSH significantly increased mRNA expression of CDH2, SNAI1, SNAIL2 and Vimentin in wild-type HCCLM3 cells, without affecting CDH1 and TWIST1 levels. Knockout of the BACH1 gene eliminated the changes in EMT-associated gene expression induced by NAC and GSH. Moreover, mRNA levels of CDH2, SNAI1, SNAIL2 and Vimentin were markedly reduced in HCCLM3 BACH1-KO cells compared to wild-type counterparts (Fig. 1I). These in vitro and in vivo findings collectively demonstrate that BACH1 knockout suppresses EMT and metastasis in HCCLM3 cells, whilst also counteracting the promotion of NAC and GSH on the EMT and metastasis of HCCLM3 cells.

We also explored the role of BACH1 in antioxidant-accelerated HCC cells migration and invasion in vitro by transwell assay. As shown in Fig. S2A–D, NAC and GSH treatments significantly increased the invasion and migration abilities of wild-type HCCLM3 and HepG2 cells, but did not affect the migration and invasion of HCCLM3 BACH1-KO and HepG2 BACH1-KO cells compared to the controls. Furthermore, the migration and invasion abilities of HepG2 BACH1-KO and HCCLM3 BACH1-KO cells were significantly lower than those of wild-type cells. This suggested that the absence of BACH1 led to significant reductions in the migration and invasion abilities of HCC cells, and almost eliminated the promotion of NAC and GSH on the invasion and migration abilities of HCC cells, which is consistent with the results obtained from the in vivo experiments. Altogether, the antioxidants NAC and GSH stimulated BACH1-dependent migration and invasion of HCC cells in vitro as well as metastasis in vivo.

### The antioxidants NAC and GSH stabilize BACH1 protein by decreasing ROS levels

**Fig. 2:**
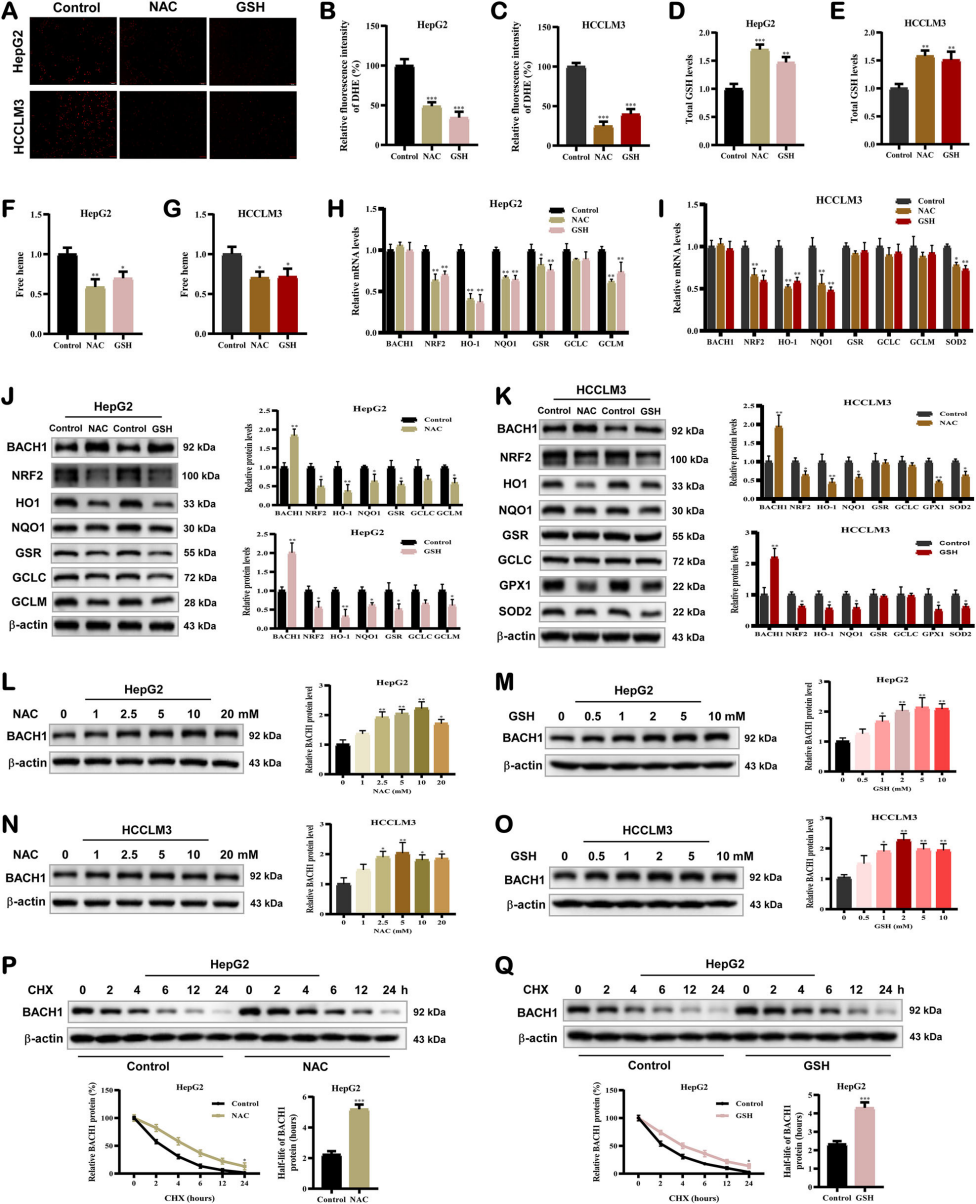
The antioxidants NAC and GSH stabilize BACH1 protein by decreasing ROS levels. **A** ROS levels in HCCLM3 and HepG2 cells after treatment with control, 5 mM NAC or 2 mM GSH for 24 h were assayed using DHE probe by fluorescence microscope. **B, C** Quantification of relative DHE fluorescence intensity in HepG2 and HCCLM3 cells treated with control, 5 mM NAC or 2 mM GSH for 24 h, corresponding to the representative images shown in **A** (*n* = 3). **D, E** Total GSH levels in HepG2 and HCCLM3 cells after treatment with control, 5 mM NAC or 2 mM GSH for 24 h (*n* = 3 × 3). **F, G** Free heme levels in HepG2 and HCCLM3 cells treated with control, 5 mM NAC or 2 mM GSH for 24 h (*n* = 3 × 3). **H** RT-qPCR analysis of BACH1, NRF2, NQO1, HO-1, GSR, GCLM and GCLC levels in HepG2 cells treated with control, 5 mM NAC or 2 mM GSH for 24 h (*n* = 3 × 3). **I** RT-qPCR analysis of BACH1, NRF2, NQO1, HO-1, GSR, GCLC, GCLM and SOD2 levels in HCCLM3 cells treated with control, 5 mM NAC or 2 mM GSH for 24 h (*n* = 3 × 3). **J** Detection and quantification of BACH1, NRF2, NQO1, HO-1, GSR, GCLM and GCLC levels in HepG2 cells treated with controls, 5 mM NAC or 2 mM GSH for 24 h by Western blotting (*n* = 3). **K** Detection and quantification of BACH1, NRF2, HO-1, NQO1, GSR, GCLC, GPX1 and SOD2 levels in HCCLM3 cells treated with controls, 5 mM NAC or 2 mM GSH for 24 h by Western blot (*n* = 3). **L, M** Western blot analysis and quantification of BACH1 protein levels in HepG2 cells after treatment with different concentrations of NAC **L** or GSH **M** for 24 h (*n* = 3). **N, O** Western blot analysis and quantification of the BACH1 protein levels in HCCLM3 cells after treated with various concentrations of NAC **N** or GSH **O** for 24 h (*n* = 3). **P** Detection and quantification of BACH1 protein levels in NAC-treated and control HepG2 cells after the incubation with 50 μg/mL cycloheximide (CHX) at various times by Western blotting. The half-life of BACH1 protein was calculated (*n* = 3). **Q** Detection and quantification of BACH1 protein levels in control and GSH-treated HepG2 cells after incubation with 50 μg/mL cycloheximide (CHX) at various times by Western blotting. The half-life of BACH1 protein was calculated (*n* = 3). Data are shown with mean ± SD (*p < 0.05, **p < 0.01, ***p < 0.001).

ROS could oxidize heme-containing proteins to facilitate free heme release [19, 30], while the higher levels of intracellular free heme was conducive to the degradation of BACH1 by proteasomes [18, 31]. In contrast, the decrease in ROS levels prevented BACH1 protein degradation in lung cancer cells by reducing the release of free heme [11]. To verify whether antioxidants NAC and GSH could also regulate BACH1 protein in a similar way in HCC cells, we first examined the changes in intracellular ROS levels after treatment with NAC or GSH. As shown in Fig. 2A–C, NAC and GSH significantly reduced the ROS levels in HepG2 and HCCLM3 cells compared with controls. To biochemically verify that antioxidant treatments (NAC and GSH) successfully altered the intracellular redox state, we quantified total GSH levels, which served as a key indicator of antioxidant capacity. Consistent with this aim, both treatments significantly increased the levels of total GSH in both types of HCC cells (Fig. 2D, E). Next, free heme was detected. As expected, NAC and GSH reduced the levels of free heme in HCCLM3 and HepG2 cells (Fig. 2F, G). The qPCR results revealed that NAC and GSH treatments led to a significant reduction in mRNA levels of NRF2 and its target genes HO-1, NQO1, GSR and GCLM in HepG2 cells without affecting BACH1 and GCLC (Fig. 2H), and also resulted in down-regulation of NRF2 and the mRNA expression of its target genes NQO1, SOD2 and HO-1 in HCCLM3 cells without affecting BACH1, GSR, GCLC and GCLM (Fig. 2I). However, western blotting results found that NAC and GSH treatments significantly increased the protein expression of BACH1 in both HepG2 and HCCLM3 cells. Furthermore, NAC and GSH treatments led to a significant reduction in protein levels of NRF2, NQO1, HO-1, GSR and GCLM in HepG2 cells without affecting GCLC. In HCCLM3 cells, protein levels of NRF2, NQO1, HO-1, SOD2 and GPX1 were markedly decreased by NAC and GSH treatments, but GSR and GCLC were not remarkably changed compared with controls (Fig. 2J, K). Notably, while NAC and GSH treatment led to an increase in BACH1 protein expression and a concurrent decrease in NRF2 mRNA levels, chromatin immunoprecipitation sequencing (ChIP-seq) analysis of BACH1 in HepG2 cells did not detect significant binding peaks within the NRF2 genomic region (Fig. S3). This indicates that BACH1 may not directly bind to or regulate NRF2 transcription. Furthermore, the downregulation of NRF2 target genes is therefore a combined consequence of reduced NRF2 expression and elevated levels of their transcriptional repressor BACH1. The above results suggested that antioxidant intervention induced a low oxidative stress status in HCC cells, reduced free heme levels and modulated BACH1 protein abundance via post-transcriptional regulation.

To further characterize this post-transcriptional regulation, we performed dose-response experiments, which showed that NAC and GSH treatments enhanced BACH1 protein expression in both HepG2 and HCCLM3 cells at moderate concentrations, whereas the magnitude of this upregulation diminished at higher doses (Fig. 2L–O). This non-linear response, coupled with the unchanged BACH1 mRNA levels, further supports that antioxidants modulate BACH1 abundance through post-transcriptional mechanisms, such as altered protein stability or translation efficiency, which may be subject to feedback regulation at higher antioxidant concentrations. Next, we performed cycloheximide (CHX) chase assays to determine whether the upregulation of BACH1 involves enhanced protein stability. As shown in Fig. 2P–Q, the degradation of BACH1 protein was significantly slowed in HepG2 cells pretreated with NAC or GSH compared to untreated controls. Quantification of band intensity revealed that the half-life of BACH1 was markedly prolonged under antioxidant conditions. This result provides direct evidence that the antioxidants NAC and GSH stabilize the BACH1 protein, thereby leading to its accumulation independently of transcription. Collectively, these indicated that the antioxidants GSH and NAC stabilized BACH1 protein by decreasing the levels of ROS.

### BACH1 facilitates HCC cell invasion and EMT

**Fig. 3:**
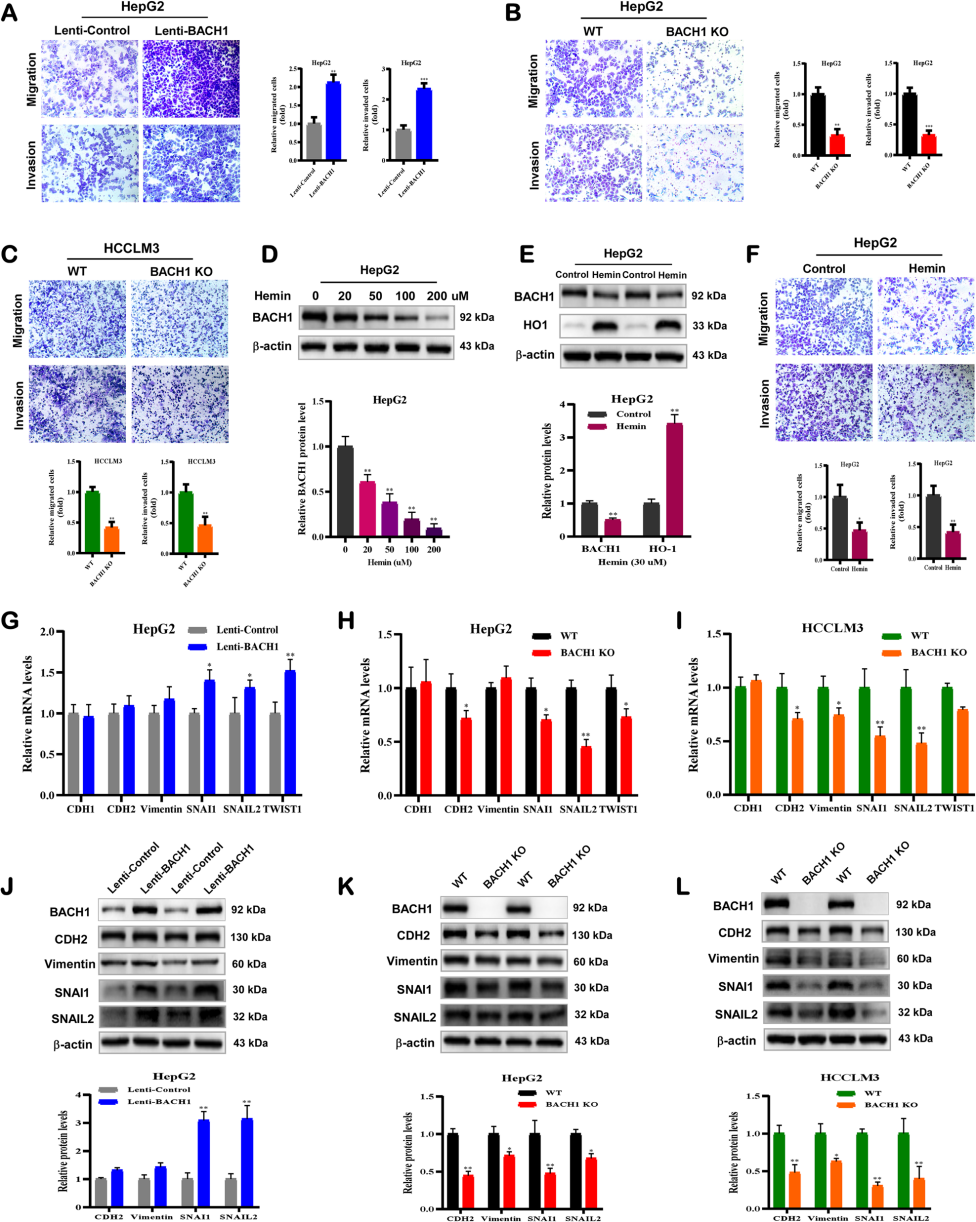
BACH1 promotes invasion and EMT of HCC cells. **A** Transwell invasion and migration assays of Lenti-Control and Lenti-BACH1 cells. Left, representative images of migrated and invaded cells (*n* = 3). **B** Transwell invasion and migration assays of HepG2 and HepG2 BACH1-KO cells. Left, representative images of migrated and invaded cells (*n* = 3). **C** Transwell invasion and migration assays of HCCLM3 and HCCLM3 BACH1-KO cells. Up, representative images of migrated and invaded cells (*n* = 3). **D** Western blot analysis and quantification of the BACH1 protein level in HepG2 cells treated with different concentrations of hemin for 24 h (*n* = 3). **E** Western blot analysis and quantification of HO-1 and BACH1 protein levels in HepG2 cells treated with control and 30 μM Hemin for 24 h (*n* = 3). **F** Transwell invasion and migration assays of HepG2 cells after treatment with control and 30 μM Hemin for 24 h. Up, representative images of migrated and invaded cells (*n* = 3). **G** RT-qPCR analysis of CDH2, Vimentin, CDH1, SNAI1, SNAIL2 and TWIST1 levels in Lenti-BACH1 and Lenti-Control cells (*n* = 3 × 3). **H** RT-qPCR analysis of CDH2, Vimentin, CDH1, SNAI1, SNAIL2 and TWIST1 levels in HepG2 BACH1-KO and HepG2 cells (*n* = 3 × 3). **I** RT-qPCR analysis of CDH1, CDH2, Vimentin, SNAI1, SNAIL2 and TWIST1 levels in HCCLM3 and HCCLM3 BACH1-KO cells (*n* = 3 × 3). **J** Detection and quantification of CDH2, Vimentin, SNAI1 and SNAIL2 levels in Lenti-BACH1 and Lenti-Control cells by Western blot (*n* = 3). **K** Detection and quantification of CDH2, Vimentin, SNAI1 and SNAIL2 levels in HepG2 BACH1-KO and HepG2 cells by Western blot (*n* = 3). **L** Detection and quantification of CDH2, Vimentin, SNAI1 and SNAIL2 levels in HCCLM3 BACH1-KO and HCCLM3 cells by Western blot (*n* = 3). Data are shown with mean ± SD (*p < 0.05, **p < 0.01, ***p < 0.001).

Then, the effect of BACH1 on HCC cell metastasis in vitro was further verified by transwell assay. In Fig. 3A, overexpression of BACH1 remarkably enhanced invasion and migration of HepG2 cells. By contrast, knockout of BACH1 in HCCLM3 and HepG2 cells reduced a marked reduction in cell invasion and migration (Fig. 3B, C). Furthermore, we utilized hemin, an oxidized form of heme, to induce BACH1 degradation. Western blot analysis showed that hemin inhibited the protein expression of BACH1 in HepG2 cells in a dose-dependent manner (Fig. 3D). Based on the dose-response results, 30 μM hemin was selected for subsequent experiments, as it effectively reduced BACH1 protein expression and induced HO-1 (Fig. 3E). Transwell assays demonstrated that hemin treatment significantly suppressed the migration and invasion abilities of HepG2 cells (Fig. 3F). These findings suggest that BACH1 promotes the metastatic behavior of HCC cells in vitro.

EMT is strongly associated with cancer metastasis, changes in expressions of EMT-related genes in HCC cells after overexpression or knockout of BACH1 were further examined. qPCR results suggested that BACH1 overexpression remarkably upregulated the EMT-related transcription factors SNAI1, SNAIL2, and TWIST1 mRNA levels, but did not affect mesenchymal markers CDH2 and Vimentin, and also epithelial marker CDH1 (Fig. 3G). Knockout of BACH1 led to marked decrease of CDH2, SNAI1, SNAIL2 and TWIST1 mRNA levels in HepG2 BACH1-KO cells without affecting CDH1 and Vimentin (Fig. 3H), and also caused down-regulation of CDH2, Vimentin, SNAI1, and SNAIL2 mRNA expression in HCCLM3 BACH1-KO cells without affecting CDH1 and TWIST1 (Fig. 3I). Western blotting revealed that BACH1 overexpression promoted protein expression of SNAI1 and SNAIL2 in HepG2 cells, but did not affect CDH2 and Vimentin protein levels (Fig. 3J). In contrast, knockout of BACH1 inhibited the protein levels of CDH2, Vimentin, SNAI1 and SNAIL2 in both HepG2 and HCCLM3 cells (Fig. 3K, L).

During cancer metastasis, tumor cells were exposed to conditions like hypoxia, inadequate nutrient supply and lactic acidosis, which contributed to the accumulation of mis-folded proteins in endoplasmic reticulum (ER), thereby causing ER stress also known as unfolded protein response (UPR) [32]. Similarly, mitochondrial dysfunction could also lead to unfolded protein accumulation, which in turn triggered the unfolded protein response of mitochondria (UPR^mt^) to repair dysfunctional mitochondria to prolong cell survival [33, 34]. Studies have previously shown that activation of UPR signaling induced the EMT-like phenotype, down-regulated epithelial markers and up-regulated mesenchymal markers to influence cancer metastasis [35]. Our results showed that knockout of BACH1 markedly down-regulated mRNA levels of UPR signaling molecules IRE1, XBP1 and ATF4 without affecting eIF2α in HepG2 cells, as well as down-regulated mRNA levels of IRE1 and XBP1 without affecting eIF2α and ATF4 in HCCLM3 cells. Moreover, mRNA levels of UPR^mt^ pathway-related genes ATF5 and FGF21 were also significantly suppressed by BACH1 knockout (Fig. S4A, B). Western blotting results revealed that knockout of BACH1 in HepG2 cells led to a significant reduction in protein expressions of IRE1, XBP1, eIF2α, and UPR^mt^ pathway-related genes FGF21, HSP60, GRP75, and decreased the protein phosphorylation of PERK (Fig. S4C), while deletion of BACH1 in HCCLM3 BACH1-KO cells down-regulated protein levels of IRE1, XBP1, eIF2α, PERK, FGF21, and GRP75 without affecting HSP60 (Fig. S4D). These results demonstrated that BACH1 knockout suppressed both EMT and the UPR/UPR^mt^ pathways. The concomitant downregulation of EMT markers and UPR signaling components suggests a potential link between BACH1-mediated UPR activation and the promotion of EMT in HCC cells. Future studies are warranted to investigate whether the suppression of UPR is a direct mechanistic contributor to the inhibition of metastasis upon BACH1 loss.

### BACH1 inhibits mitochondrial electron transport chain and activates glycolysis

**Fig. 4:**
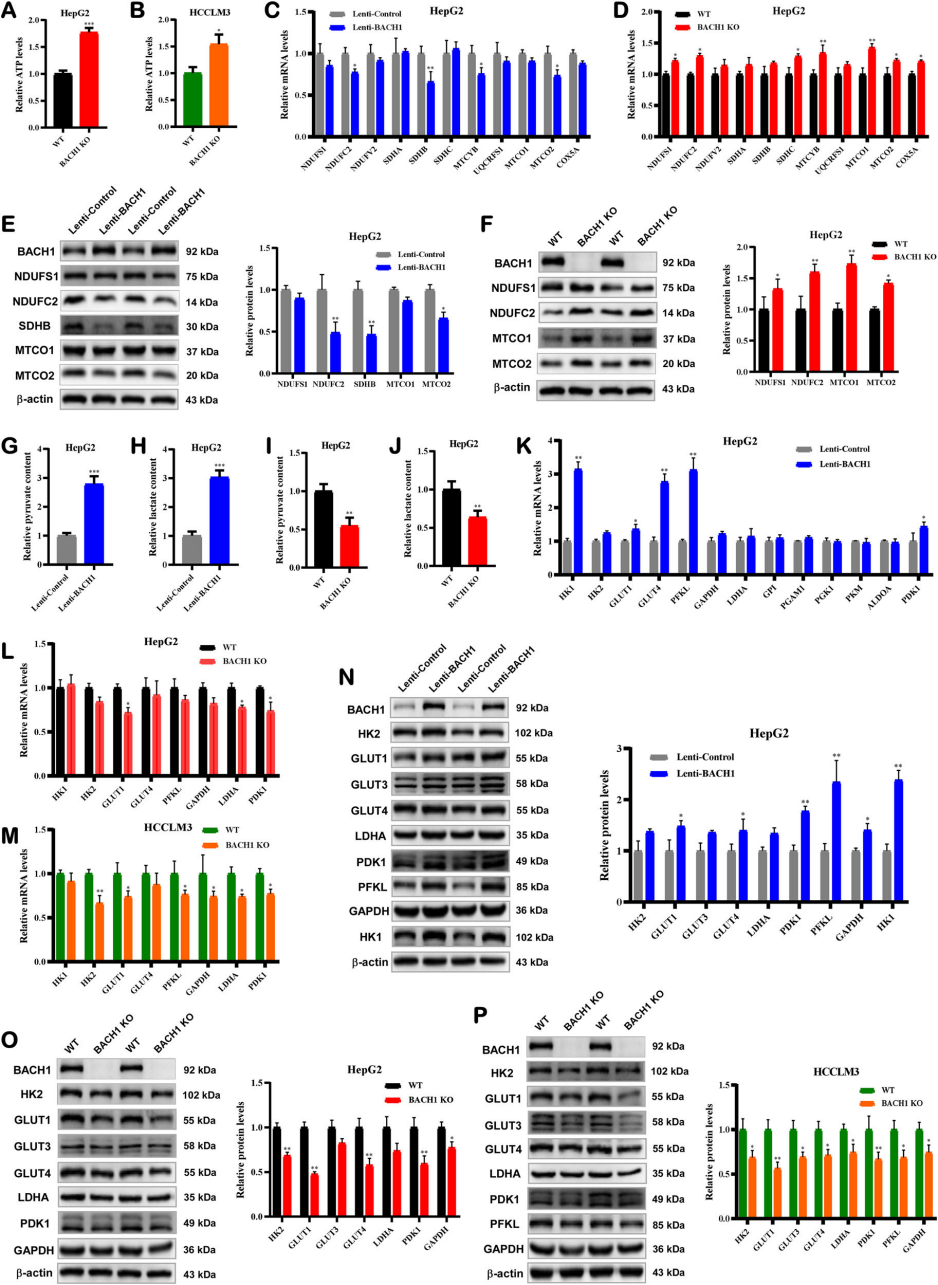
BACH1 inhibits mitochondrial electron transport chain and activates glycolysis. **A** ATP levels in HepG2 BACH1-KO and HepG2 cells (*n* = 3 × 3). **B** ATP levels in HCCLM3 BACH1-KO and HCCLM3 cells (*n* = 3 × 3). **C** Detection of NDUFS1, NDUFC2, NDUFV2, SDHA, SDHB, SDHC, MTCYB, UQCRFS1, MTCO1, MTCO2 and COX5A levels in Lenti-BACH1 and Lenti-Control cells by RT-qPCR (*n* = 3 × 3). **D** Detection of NDUFS1, NDUFC2, NDUFV2, SDHA, SDHB, SDHC, MTCYB, UQCRFS1, MTCO1, MTCO2 and COX5A levels in HepG2 BACH1-KO and HepG2 cells by RT-qPCR (*n* = 3 × 3). **E** Western blotting analysis and quantification of NDUFS1, NDUFC2, SDHB, MTCO1 and MTCO2 levels in Lenti-BACH1 and Lenti-Control cells (*n* = 3). **F** Western blotting analysis and quantification of NDUFS1, NDUFC2, MTCO1 and MTCO2 levels in HepG2 BACH1-KO and HepG2 cells (*n* = 3). **G, H** The contents of pyruvate **G** and lactate **H** in Lenti-BACH1 and Lenti-Control cells (*n* = 3 × 3). **I, J** The contents of pyruvate **I** and lactate **J** in HepG2 BACH1-KO and HepG2 cells (*n* = 3 × 3). **K** RT-qPCR analysis of HK1, HK2, GLUT1, GLUT4, PFKL, GAPDH, LDHA, GPI, PGAM1, PGK1, PKM, ALDOA and PDK1 levels in Lenti-BACH1 and Lenti-Control cells (*n* = 3 × 3). **L** RT-qPCR analysis of HK1, HK2, GLUT1, GLUT4, PFKL, GAPDH, LDHA and PDK1 levels in HepG2 BACH1-KO and HepG2 cells (*n* = 3 × 3). **M** RT-qPCR analysis of HK1, HK2, GLUT1, GLUT4, PFKL, GAPDH, LDHA and PDK1 levels in HCCLM3 BACH1-KO and HCCLM3 cells (*n* = 3 × 3). **N** Detection and quantification of HK2, GLUT1, GLUT3, GLUT4, LDHA, PDK1, PFKL, GAPDH and HK1 levels in Lenti-BACH1 and Lenti-Control cells by Western blot (*n* = 3). **O** Detection and quantification of HK2, GLUT1, GLUT3, GLUT4, LDHA, PDK1 and GAPDH levels in HepG2 BACH1-KO and HepG2 cells by Western blot (*n* = 3). **P** Detection and quantification of HK2, GLUT1, GLUT3, GLUT4, LDHA, PDK1, PFKL and GAPDH levels in HCCLM3 BACH1-KO and HCCLM3 cells by Western blot (*n* = 3). Data are presented with mean ± SD (*p < 0.05, **p < 0.01, ***p < 0.001).

Knockout of BACH1 inhibited the UPR^mt^ pathway. To further clarify the impact of BACH1 on the mitochondrial function in HCC cells, we first examined the changes of ATP levels after BACH1 knockout. As shown in Fig. 4A, B, deletion of BACH1 in HCCLM3 BACH1-KO and HepG2 BACH1-KO cells markedly elevated ATP levels compared to control. Next, effects of BACH1 on expressions of some mitochondrial electron transport chain (ETC) complex subunit genes in HepG2 cells was analyzed by qPCR and Western blotting. The results demonstrated that BACH1 overexpression significantly suppressed mRNA levels of NDUFC2, SDHB, MTCYB and MTCO2 without affecting NDUFS1, NDUFV2, SDHA, SDHC, UQCRFS1, MTCO1 and COX5A (Fig. 4C). By contrast, knockout of BACH1 up-regulated mRNA expressions of NDUFS1, NDUFC2, SDHC, MTCYB, MTCO1, MTCO2 and COX5A, but had no remarkable effect on mRNA levels of NDUFV2, SDHA, SDHB and UQCRFS1 (Fig. 4D). Western blotting analysis were consistent. BACH1 overexpression reduced protein levels of NDUFC2, SDHB and MTCO2 without affecting NDUFS1 and MTCO1 (Fig. 4E). In contrast, the protein levels of NDUFS1, NDUFC2, MTCO1 and MTCO2 were markedly elevated in HepG2 BACH1-KO cells compared to controls (Fig. 4F). These results indicated that BACH1 knockout enhanced ATP production in HCC cells by promoting the expression of ETC complex subunit genes to activate mitochondrial oxidative phosphorylation (OXPHOS), while BACH1 overexpression inhibited OXPHOS process by down-regulating ETC complex subunit genes.

Previous studies revealed ETC deficient cancer cells could not produce mitochondrial ATP, but they were still able to proliferate using ATP produced by glycolysis [36]. Cancer cells reprogrammed themselves metabolically to adapt more efficiently to environmental changes and lack of nutritional conditions, and the Warburg effect is one of the most common metabolic reprogramming, characterized by elevated lactate production and glucose uptake [37, 38]. The acidic extracellular microenvironment resulting from lactate has been proven to facilitate cancer cell metastasis, EMT, immune escape and drug resistance [39]. We found that overexpression of BACH1 resulted in markedly greater levels of pyruvate and lactate in HepG2 cells than controls (Fig. 4G, H). Conversely, knockout of BACH1 significantly suppressed pyruvate and lactate levels in HepG2 cells (Fig. 4I, J). These indicated that BACH1 could activate glycolysis. Then, we further employed qPCR and Western blotting to investigate the effects of BACH1 on the expression of glycolytic pathway key genes in HCC cells. qPCR results demonstrated that mRNA levels of HK1, GLUT1, GLUT4, PFKL and PDK1 were up-regulated after BACH1 overexpression, whereas HK2, GAPDH, LDHA, GPI, PGAM1, PGK1, PKM and ALDOA were not significantly changed (Fig. 4K). Furthermore, BACH1 knockout suppressed mRNA expressions of GLUT1, LDHA and PDK1 in HepG2 cells without affecting HK1, HK2, GLUT4, PFKL, GAPDH, and also down-regulated mRNA levels of HK2, GLUT1, PFKL, GAPDH, LDHA and PDK1 in HCCLM3 cells without influencing HK1 and GLUT4 (Fig. 4L, M). Western blotting analysis showed that BACH1 overexpression in HepG2 cells promoted protein expressions of GLUT1, GLUT4, PDK1, PFKL, GAPDH and HK1, but had no remarkable effect on protein levels of HK2, GLUT3 and LDHA (Fig. 4N). Conversely, deletion of BACH1 in HepG2 BACH1-KO cells resulted in decreased protein levels of HK2, GLUT1, GLUT4, PDK1 and GAPDH, but did not affect GLUT3 and LDHA, whereas deletion of BACH1 in HCCLM3 BACH1-KO cells resulted in decreased protein levels of HK2, GLUT1, GLUT3, GLUT4, LDHA, PDK1, PFKL and GAPDH protein expressions to varying degrees (Fig. 4O, P). Taken together, BACH1 inhibited mitochondrial ETC and activated glycolysis thereby facilitated the Warburg effect in HCC cells. To more clearly illustrate the consistent regulatory effects of BACH1 in HCC, we summarized the genes/proteins that were consistently regulated by BACH1 in both HepG2 (KO/Lenti) and HCCLM3 (KO) cells in this study. As shown in Table S2, in addition to the significant suppression of genes and proteins related to canonical antioxidant signaling pathways by BACH1, a panel of EMT master regulators and core glycolytic transporters/enzymes exhibited consistent activation by BACH1 across the two HCC cell models and under both experimental conditions (KO/Lenti). Specifically, BACH1 markedly promoted the expression of SNAI1 and SNAI2, the pivotal transcription factors that drive EMT, and simultaneously upregulated the expression of key glycolytic molecules including glucose transporters GLUT1/GLUT4, pyruvate dehydrogenase kinase PDK1, and glycolytic enzyme GAPDH. All these alterations were consistent in HepG2 BACH1-KO cells, HCCLM3 BACH1-KO cells, and HepG2 Lenti-BACH1 cells compared with corresponding control cells, and were validated by RT-qPCR and/or Western blot analysis. These findings confirm that BACH1 functions as a positive upstream activator of EMT progression and glycolytic flux in HCC.

### Glycolysis mediates the facilitation of BACH1 on migration and invasion of HCC cells

**Fig. 5:**
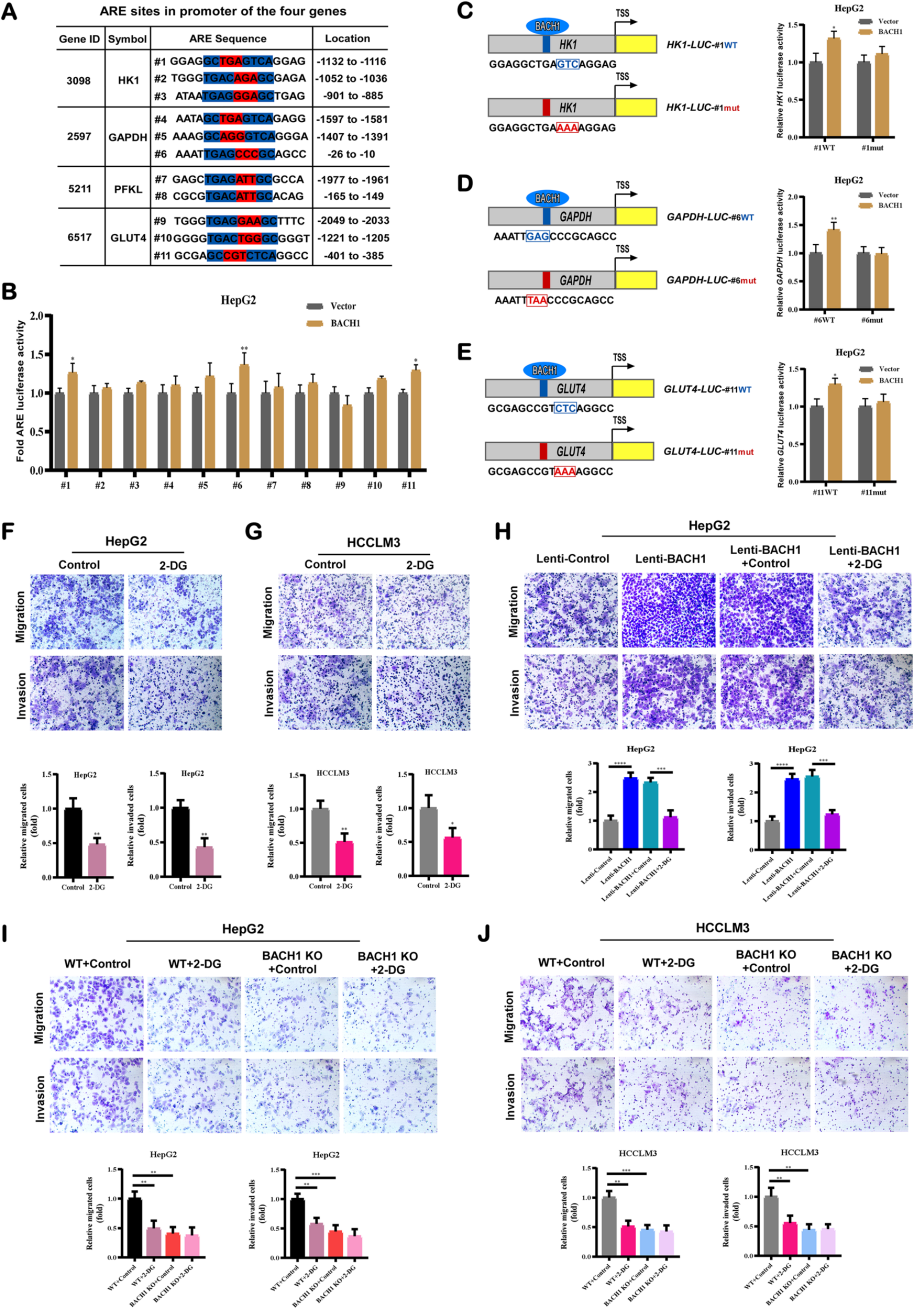
Glycolysis mediates the promotion of BACH1 on invasion and migration of HCC cells. **A** Sequences and locations of ARE-consensus DNA binding sites in HK1, GAPDH, PFKL and GLUT4 promoter **B** The effects of BACH1 on luciferase activation at AREs of HK1, GAPDH, PFKL and GLUT4 promoter in HepG2 cells (*n* = 3 × 3). **C** Schematic diagram of mutation at ARE #1 site on the HK1 promoter. Right, effects of BACH1 on luciferase activation at wild-type ARE #1 site and mutant ARE #1 site on the HK1 promoter in HepG2 cells (*n* = 3 × 3). **D** Schematic diagram of mutation at ARE #6 site on the GAPDH promoter. Right, effects of BACH1 on luciferase activation at wild-type ARE #6 site and mutant ARE #6 site on the GAPDH promoter in HepG2 cells (*n* = 3 × 3). **E** Schematic diagram of mutation at ARE #11 site on the GLUT4 promoter. Right, effects of BACH1 on luciferase activation at wild-type ARE #11 site and mutant ARE #11 site on the GLUT4 promoter in HepG2 cells (*n* = 3 × 3). Transwell invasion and migration assays of HepG2 **F** and HCCLM3 **G** cells after treatment with control and 5 mM 2-DG for 24 h. Up, representative images of migrated and invaded cells (*n* = 3). **H** Transwell invasion and migration assays of Lenti-BACH1 cells, Lenti-Control cells and control or 5 mM 2-DG treated Lenti-BACH1 cells. Up, representative images of migrated and invaded cells (*n* = 3). **I** Transwell invasion and migration assays of HepG2 and HepG2 BACH1-KO cells after treatment with control and 5 mM 2-DG for 24 h, respectively. Up, representative images of migrated and invaded cells (*n* = 3). **J** Transwell invasion and migration assays of HCCLM3 and HCCLM3 BACH1-KO cells after treatment with control and 5 mM 2-DG for 24 h, respectively. Up, representative images of migrated and invaded cells (*n* = 3). Data are reported with mean ± SD (*p < 0.05, **p < 0.01, ***p < 0.001, ****p < 0.0001).

To determine whether BACH1 directly regulates the transcription of glycolysis-related genes, we performed ChIP-seq analysis in HepG2 cells. As shown in Fig. S5A–D, the prominent enrichment of red peaks over the input signals at these gene loci indicated BACH1 binding at the genomic regions of key glycolysis-associated genes HK1, GAPDH, PFKL and GLUT4. Then, we analyzed the promoter region sequences of these genes and identified several ARE consensus DNA-binding sites, which are known to be the canonical binding motifs for BACH1 (Fig. 5A). Further dual luciferase reporter gene assay demonstrated that #1 ARE in HK1 promoter, #6 ARE in GAPDH promoter and #11 ARE in GLUT4 promoter exhibited significant increases in luciferase activity following BACH1 overexpression in HepG2 cells (Fig. 5B). When the same assay was performed in HEK293 cells, we detected BACH1-mediated activation at overlapping but distinct sites (sites #2, #5, #11), with the magnitude of activation and the set of responsive AREs differing from those in HepG2 cells (Fig. S5E). The discrepancy in responsive ARE sites between HepG2 and HEK293 cells can be attributed to the cell-type-specific transcriptional landscape. Specifically, transcriptional activity of BACH1 depends on co-regulatory proteins that are differentially expressed in hepatic versus non-hepatic cells. HepG2 cells express liver-enriched transcription factors and co-factors that may stabilize BACH1 binding to certain *ARE*s, whereas HEK293 cells lack these hepatic-specific factors, altering the pattern of functional *ARE* activation. To functionally validate the identified *ARE* sites, we performed site-directed mutagenesis in HepG2 cells. As shown in Fig. 5C–E, mutation of the core *ARE* sequences in the promoters of HK1 (#1 site), GAPDH (#6 site), and GLUT4 (#11 site) completely abolished the transcriptional activation by BACH1 overexpression. Collectively, these demonstrated that the identified *ARE* sites were directly required for BACH1 to enhance the transcription of glycolysis-related genes in HepG2 cells. These suggested that BACH1 activated transcriptional expression of HK1, GAPDH and GLUT4 genes by binding to specific ARE sites on their promoters, which in turn promoted cellular glycolytic pathway.

Next, the role of glycolysis in BACH1 regulating HCC cell migration and invasion was explored via the inhibition of glycolysis by 2-DG. We treated HCC cells with 2-DG at concentrations that did not alter baseline cell apoptosis and viability (Fig. S5F–I). Transwell assay revealed that 2-DG treatment notably depressed invasion and migration of HCCLM3 and HepG2 cells (Fig. 5F, G). To further validate the specificity of glycolysis in driving metastasis, we inhibited alternative energy metabolism pathways, including lipid oxidation (using Etomoxir) and TCA cycle function (using UK-5099). Both treatments caused a marked reduction in ATP production in HepG2 and HCCLM3 cells (Fig. S5J–M), confirming effective pathway inhibition. However, at concentrations that did not affect cell viability (Fig. S5N–Q), neither etomoxir nor UK-5099 significantly altered the migration capacity of HCC cells (Fig. S5R–U). These findings demonstrate that the suppression of metastasis is specifically linked to glycolysis inhibition, rather than a general reduction in cellular energy levels. Furthermore, we also treated BACH1-overexpressing and BACH1-knockout HCC cells with 2-DG to investigate whether BACH1 promotes HCC metastasis through glycolysis. In Lenti-BACH1 cells, the increased invasion and migration by BACH1 overexpression were effectively reversed by 2-DG and restored to a level close to that of the control cells (Fig. 5H). In contrast, 2-DG treatment had only a minimal effect on migration and invasion in HepG2 BACH1-KO and HCCLM3 BACH1-KO cells (Fig. 5I, J). This was in stark contrast to its robust inhibitory effect in wild-type cells, indicating that glycolysis mediated the promotion of BACH1 on HCC cell migration and invasion.

### BACH1 regulates metabolic reprogramming in HCC cells

**Fig. 6:**
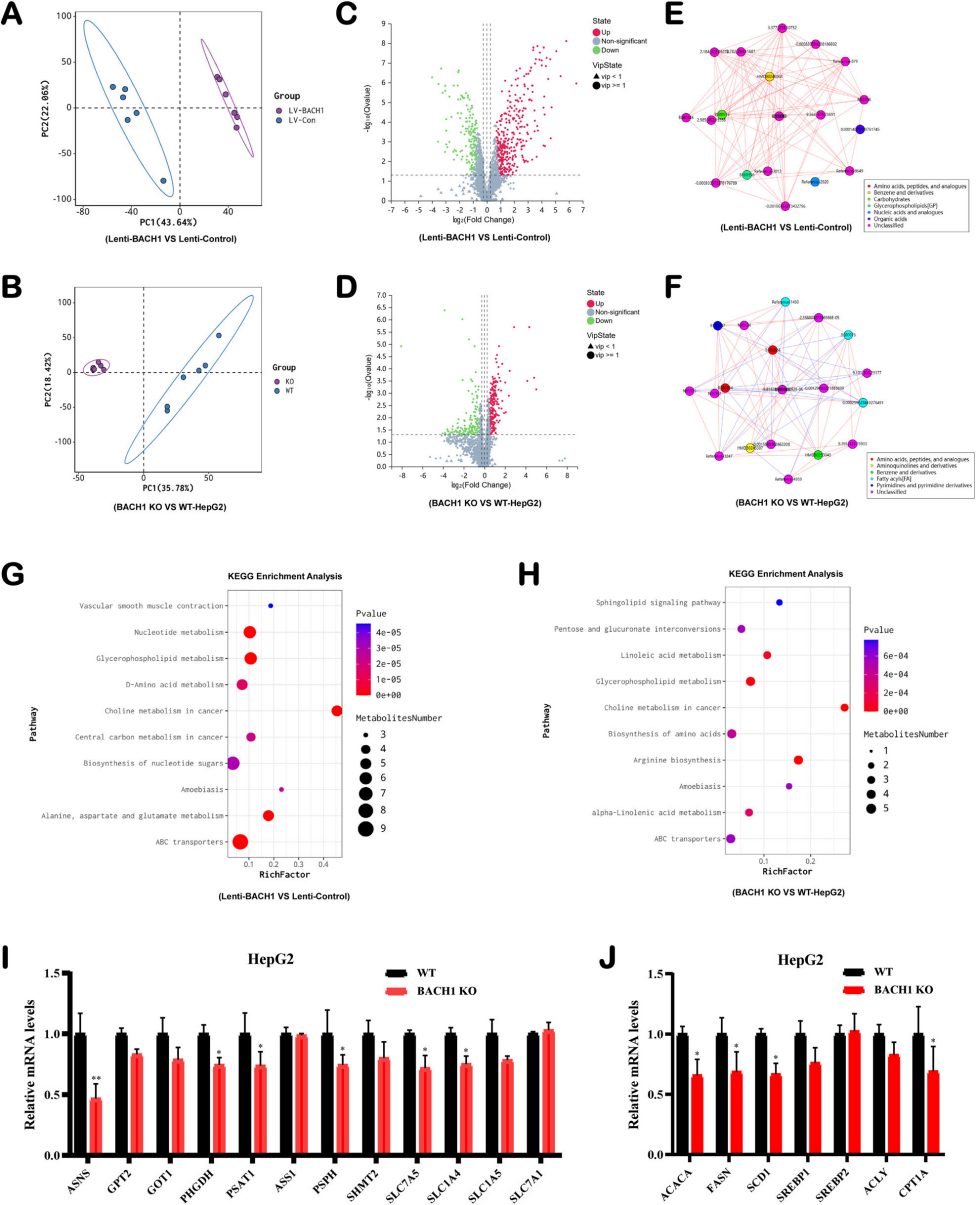
BACH1 regulates metabolic reprogramming in HCC cells. **A** The PLS-DA of Lenti-BACH1 and Lenti-Control cells samples. **B** The PLS-DA of HepG2 BACH1-KO and HepG2 cells samples. **C, D** Volcano plots of differential metabolites in Lenti-BACH1 compared to Lenti-Control **C** and HepG2 BACH1-KO versus HepG2 **D** cells, markedly upregulated differential metabolites are indicated in red, markedly downregulated differential metabolites in green and gray represents insignificant metabolites. Correlation network diagrams of differential metabolites in Lenti-BACH1 versus Lenti-Control **E** and HepG2 BACH1-KO versus HepG2 **F** cells, the color of lines represents correlation, red lines indicate positive correlation and blue lines indicate negative correlation. **G, H** KEGG metabolic pathways enrichment analyses of differential metabolites in Lenti-BACH1 compared to Lenti-Control **G** and HepG2 BACH1-KO versus HepG2 **H** cells, the top 10 significantly enriched metabolic pathways are shown in bubble charts. **I** Detection of ASNS, GPT2, GOT1, PHGDH, PSAT1, ASS1, PSPH, SHMT2, SLC7A5, SLC1A4, SLC1A5 and SLC7A1 levels in HepG2 BACH1-KO and HepG2 cells by RT-qPCR (*n* = 3 × 3). **J** Detection of ACACA, FASN, SCD1, SREBP1, SREBP2, ACLY and CPT1A levels in HepG2 BACH1-KO and HepG2 cells by RT-qPCR (*n* = 3 × 3). The data are presented with mean ± SD (*p < 0.05, **p < 0.01).

Our results confirmed that BACH1 facilitated Warburg effect. To gain a fuller understanding of BACH1 regulation on metabolic reprogramming in HCC cells, non-targeted metabolomic sequencing was performed on Lenti-Control, Lenti-BACH1, HepG2 and HepG2 BACH1-KO cells. Figure 6A, B showed the distribution of each sample in the partial least squares discriminant analysis (PLS-DA) model. It could be seen that Lenti-BACH1 and Lenti-Control cells were obviously separated, as well as a good discrimination existed between HepG2 and HepG2 BACH1-KO groups. Volcano plots of differential metabolites indicated that 338 metabolites were upregulated and 159 were downregulated in Lenti-BACH1 cells versus Lenti-Control cells, whereas 212 metabolites were upregulated and 144 were downregulated in HepG2 BACH1-KO cells versus HepG2 cells (Fig. 6C, D). Next, spearman correlation coefficients between pairwise differential metabolites in Lenti-BACH1 compared to Lenti-Control and HepG2 BACH1-KO compared to HepG2 cells were calculated respectively, and the metabolite pairs with |r | > 0.8 and *p* < 0.05 were displayed as correlation network diagrams. As shown in Fig. 6E, F, metabolite nodes such as peptides and analogues, amino acids, benzene and derivatives, carbohydrates, glycerophospholipids (GP) had more edges connected in Lenti-BACH1 versus Lenti-Control cells, whereas peptides and analogues, amino acids, aminoquinolines and derivatives, benzene and derivatives, fatty acyls in HepG2 BACH1-KO versus HepG2 cells, suggesting their central position in networks. Further KEGG metabolic pathway enrichment analyses of differential metabolites revealed that significantly enriched metabolic pathways in both Lenti-BACH1 versus Lenti-Control and HepG2 BACH1-KO versus HepG2 cells mainly included glycerophospholipid metabolism, choline metabolism in cancer, amoebiasis and ABC transporters. In addition, the top 10 metabolic pathways significantly enriched in BACH1 overexpression group also included vascular smooth muscle contraction, central carbon metabolism in cancer, D-amino acid metabolism, nucleotide metabolism, biosynthesis of nucleotide sugars, alanine, aspartate and glutamate metabolism, whereas BACH1 knockout group also included sphingolipid signaling pathway, pentose and glucuronate interconversions, linoleic acid metabolism, biosynthesis of amino acids, arginine biosynthesis, alpha-linolenic acid metabolism (Fig. 6G, H).

On the basis of metabolomic analysis, effects of BACH1 on gene expressions related to amino acid synthesis and transport were verified by qPCR. The results showed that knockout of BACH1 in HepG2 cells markedly reduced mRNA levels of amino acid synthesis-related genes ASNS, PHGDH, PSAT1, PSPH and amino acid transport-related genes SLC7A5 and SLC1A4, without affecting GPT2, GOT1, ASS1, SHMT2, SLC1A5 and SLC7A1 (Fig. 6I). Moreover, the mRNA levels of fatty acid synthesis-related genes ACACA, FASN, SCD1 and CPT1A were also markedly decreased by BACH1 knockout, while SREBP1, SREBP2 and ACLY showed insignificant changes (Fig. 6J). Altogether, BACH1 regulated the metabolic reprogramming in HCC cells.

### Reconstitution of BACH1 reverses the metabolic and migratory phenotypes of HepG2 BACH1‑KO cells

To further and more rigorously verify the functional role of BACH1, we reconstituted BACH1 expression in HepG2 BACH1‑KO cells using lentiviral delivery, generating a stable BACH1 reconstitution cell line (HepG2 BACH1-KO + Lenti-BACH1) and a corresponding parallel control cell line (HepG2 BACH1-KO + Lenti-Control). Western blot analysis confirmed effective restoration of BACH1 protein levels in the HepG2 BACH1-KO + Lenti-BACH1 cells (Fig. S6A). Then, we validated the expression of glycolysis-related genes, ETC complex subunit genes, and EMT-related genes in WT-HepG2 BACH1-KO HepG2 BACH1-KO + Lenti-Control and HepG2 BACH1-KO + Lenti-BACH1 cells. The results revealed that the suppressed expression of glycolysis-related genes HK2, GLUT1, GLUT4, LDHA and PDK1 in BACH1-KO cells was significantly rescued upon BACH1 re-expression (Fig. S6B). Concurrently, the upregulation of ETC complex subunit genes NDUFS1, NDUFC2, MTCO1 and MTCO2 observed in BACH1-KO cells was significantly reversed after reconstitution of BACH1 (Fig. S6C). Moreover, the alterations in EMT markers, including CDH2, Vimentin, SNAI1, and SNAIL2, were largely restored in the BACH1-KO + Lenti-BACH1 cells (Fig. S6D).

In functional assays, the impaired migration and invasion capabilities caused by BACH1 knockout were markedly recovered following reconstitution of BACH1 (Fig. S6E). Interestingly, we previously observed that antioxidants NAC and GSH enhanced migration and invasion in WT-HepG2 cells, but this pro-migratory effect was abolished in BACH1-KO cells. However, in BACH1-KO + Lenti-BACH1 cells, the promotive effect of antioxidants on migration and invasion was partially restored (Fig. S6F), further confirming the critical role of BACH1 in antioxidant-induced HCC metastasis. Together, the rescue of molecular and functional phenotypes upon BACH1 reconstitution confirms its indispensable role in promoting glycolysis, suppressing mitochondrial respiration, and inducing EMT. Notably, the dependency of antioxidant-promoted metastasis on BACH1 unveils its unique integrative role at the nexus of metabolism and redox signaling.

## Discussion

**Fig. 7:**
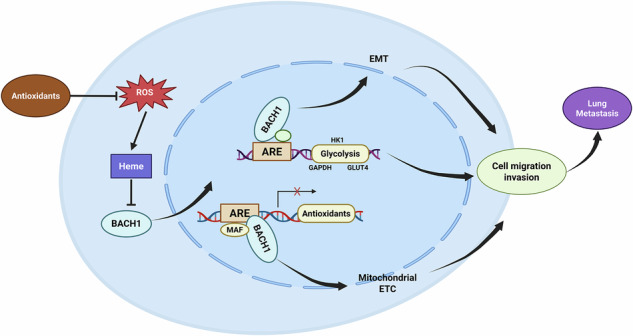
Schematic representation of the mechanism that BACH1 stabilization by antioxidants facilitates HCC metastasis. Antioxidants stabilize BACH1 protein through reducing ROS and free heme levels, thus stimulating BACH1-dependent HCC metastasis. BACH1 promotes migration and invasion of HCC cells by enhancing EMT. BACH1 also activates the transcription of key genes in glycolysis and represses the expression of mitochondrial ETC genes, thereby facilitating Warburg effect and glycolysis-mediated cell migration and invasion. This figure was created with BioRender.com.

ROS is a class of unstable oxygen-containing molecules and prevalent by-products in the process of cellular metabolism [40]. ROS at physiological level is the major signaling molecule that regulates cell differentiation, proliferation, motility, angiogenesis, and appropriate intracellular ROS levels are essential for maintaining normal cellular function [41]. However, overproduction of ROS and its induced oxidative stress can damage biomolecules like DNA, proteins and lipids, leading to diseases such as cancer [42–44]. The role of ROS and antioxidants in cancer occurrence and progression has long been controversial. Antioxidants have been proven to accelerate migration and invasion of HCC cells [13], but its underlying mechanisms are still unclear. In this study, we found that antioxidants stabilized BACH1 protein by decreasing ROS and free heme levels, thereby stimulating BACH1-dependent in vitro invasion, migration and metastasis in vivo of HCC cells. Mechanistically, BACH1 promoted cell migration and invasion by enhancing EMT. On the other hand, BACH1 activated transcription of key genes in glycolysis while repressing expression of mitochondrial ETC complex subunit genes to promote the Warburg effect and glycolysis-mediated invasion and migration of HCC cells (Fig. 7).

Previous studies have revealed that endogenous antioxidant transcription factor NRF2 promoted the metastasis of lung cancer by suppressing heme and FBXO22-mediated degradation of BACH1, whereas exogenous antioxidants suppressed BACH1 degradation via reducing ROS levels and the release of free heme, which was also capable of facilitating lung cancer metastasis [11, 45]. Our results found that in HCC cells, antioxidants treatment down-regulated expressions of NRF2 and its targeted genes, promoted the expression and stability of BACH1 protein by reducing ROS and free heme levels, thus accelerating BACH1-dependent HCC metastasis. Interestingly, NAC and GSH treatment also reduced NRF2 mRNA levels. Although BACH1 stabilization was observed under these conditions, our BACH1 ChIP-seq analysis did not reveal direct binding to the NRF2 genomic region, suggesting that the decrease in NRF2 transcription is unlikely to be mediated by direct BACH1 repression. Instead, we speculate that indirect mechanisms may be involved. For instance, BACH1 might regulate the expression of other transcription factors that control NRF2 transcription, or the altered redox state could affect the activity of additional redox‑sensitive transcription factors [15, 46]. Alternatively, post-transcriptional regulation of NRF2 mRNA stability might contribute [47]. Further studies are warranted to dissect these possibilities. Therefore, inducing the release of free heme through pro-oxidants to stimulating BACH1 protein degradation could be a potential strategy for the therapeutic of HCC metastasis.

EMT is thought to be a key mechanism for promoting tumor metastasis and involves multiple dynamic changes in cells and tissues, as the loss of cell polarity and down-regulation of epithelial cell adherence molecules, enhancing migration and the ability to invade adjacent tissues [48]. This process is coordinated and dynamically regulated by SNAIL and ZEB transcriptional repressors of epithelial genes [49]. Our studies revealed that BACH1 promoted the expression of transcription factors SNAI1 and SNAIL2 in HCC cells, enhancing EMT as well as cell migration and invasion. Consistently, previous work has demonstrated that BACH1 promotes EMT by activating SNAI2 in pancreatic cancer [50], supporting a conserved pro‑EMT function of BACH1 across cancer types. Furthermore, a large amount of evidence indicated that UPR activation contributed to tumor progression by modulating cancer characteristics, including the invasion-metastasis cascade reaction [51, 52]. For instance, lysyl oxidase-like 2 (LOXL2) overexpression induced expression of multiple EMT transcription factors via activating IRE1-XBP1 signaling pathway, with SNAI1, SNAIL2, ZEB2 and TCF3 being direct targets of XBP1 [53]. In mammary epithelial cells, serine protease inhibitor SERPINB3 induced adaptive UPR, thereby activating expression of NF-κB, IL-6 and promoting EMT [54]. Similarly, UPR^mt^ has also been proven to facilitate migration and invasion of cancer cells by enhancing EMT [55]. In our study, we found that BACH1 knockout in HCC cells concurrently suppressed the EMT program and the activation of both the UPR and UPR^mt^ pathways. Given the established role of UPR signaling in promoting an EMT-like phenotype and metastasis under stress conditions, our findings lead us to hypothesize a plausible model. BACH1 may drive HCC metastasis not only through its direct transcriptional targets but also potentially by sustaining the activation of cellular stress response pathways (UPR/UPR^mt^), thereby fostering a pro-metastatic cellular state. However, we acknowledge that our current data establish a potential association rather than a definitive causal relationship between the suppression of UPR/UPR^mt^ and the inhibition of metastasis upon BACH1 loss. It remains to be determined whether the downregulation of UPR is a direct driver of the reduced metastatic capacity, or a parallel event downstream of BACH1. To directly test this potential mechanistic link, future studies employing rescue experiments will be crucial. For example, investigating whether the impaired migration and invasion in BACH1-knockout cells can be reversed by reactivating specific UPR branches would help establish UPR as a functional conduit for BACH1-promoted metastasis. Additionally, exploring whether BACH1 transcriptionally regulates key UPR/UPR^mt^ components will deepen the mechanistic understanding. Despite this remaining question, our work expands the functional scope of BACH1 in HCC by connecting it to cellular stress response pathways, positioning it as a potential node integrating stress adaptation with invasive behavior. Notably, a recent study demonstrated that BACH1 can indirectly induce the expression of FGF21, a key effector molecule of the UPR^mt^ pathway [56], further supporting a regulatory link between BACH1 and mitochondrial stress responses.

Additional exploration of the effects of BACH1 on mitochondrial function showed that knockout of BACH1 activated mitochondrial OXPHOS by promoting expression of ETC complex subunit genes and then enhanced ATP production in HCC cells. Conversely, BACH1 overexpression inhibited OXPHOS process through the down-regulation of ETC complex subunit genes. In differentiated SH-SY5Y cells, hemin alleviated lead-induced impairment of OXPHOS and reduced ATP, and knockout of BACH1 could salvage lead-induced downregulation of OXPHOS-related gene transcription and expression [57]. In breast cancer, BACH1 has been reported to negatively regulate transcription of ETC genes to promote metastasis [58], which is consistent with the regulatory role of BACH1 on mitochondrial function observed in the present study. However, mitochondrial OXPHOS was suppressed in BACH1 overexpressing HCC cells while cell invasion and migration were increased, indicating that cancer cells needed to generate energy through other metabolic pathways to satisfy their demand. It was well known that even under aerobic conditions, cancer cells consume more glucose to produce lactic acid, i.e., the “Warburg effect”, and this metabolic reprogramming of cells was considered to be one of the characteristics of cancer [59, 60]. Our findings demonstrated that BACH1 promoted the production of pyruvate and lactate, and enhanced the expression of glycolysis-related genes in HCC cells. Mechanistic investigations further revealed that BACH1 facilitated the transcription of glycolytic key enzymes HK1 and GAPDH, as well as the glucose transporter gene GLUT4 by binding to *ARE* sites on their promoters, thereby activating glycolytic pathway. Meanwhile, the acidic tumor microenvironment resulting from increased lactate has been proven to favor cancer cells’ invasion and metastasis. We also found that 2-DG, an inhibitor of glycolytic pathway, suppressed the migration and invasion of HCC cells, whereas alterations in other energy production pathways (lipid oxidation and TCA cycle) had minimal effects on Migration phenotype. Notably, 2-DG treatment significantly reversed the enhanced migration and invasion induced by BACH1 overexpression, while exerting negligible effects on the already reduced migratory and invasive capacities of BACH1-KO cells. Previous studies have established that the deubiquitinating enzyme USP47 stabilized BACH1 through interacting with it, activated BACH1-mediated HK2 and GAPDH transcription and promoted the Warburg effect as well as the progression of non-small cell carcinoma [61]. In microglia, the absence of BACH1 reduced lactic acid production by lowering HK2 and GAPDH transcription [62]. In HCC, Hemin-induced BACH1 inhibition significantly down-regulated the expression of HK2 and reduced glycolysis and lymph node metastasis caused by UBR7 knockout [63]. Collectively, our results are consistent with these prior findings, confirming that BACH1 functions as a key activator of glycolysis. This activation drives the Warburg effect and, consequently, promotes HCC cell invasion and migration driven by glycolysis.

While our study establishes BACH1 as a direct transcriptional regulator of key glycolytic genes, as evidenced by its genomic loci binding (ChIP-seq) and the functional necessity of specific ARE motifs (luciferase/mutagenesis assays). This molecular capacity directly translates to enhanced glycolytic flux and a Warburg-like metabolic phenotype, providing bioenergetic basis for BACH1-promoted invasion and migration. However, an intriguing observation is that genetic ablation of BACH1 did not significantly alter the mRNA levels of HK1, GAPDH and GLUT4 under standard culture conditions. This observation, while initially seeming at odds with the direct transactivation data, underscores the robustness and redundancy inherent in core metabolic networks. Essential pathways like glycolysis are often buffered by parallel transcription factors (e.g., HIF-1α, c-Myc) [64, 65] and homeostatic mechanisms, which likely compensate to maintain basal expression upon the loss of a single regulator like BACH1. Therefore, the unchanged mRNA levels do not negate the direct regulatory role of BACH1, but rather refine our understanding of its physiological function. BACH1’s transcriptional impact may be most critical under specific stresses (e.g., hypoxia, nutrient deprivation) where its activity provides a competitive advantage by rapidly boosting glycolytic capacity beyond basal levels. Future work under pathophysiological conditions will further elucidate the dynamic contribution of BACH1 to metabolic reprogramming. Furthermore, we performed non-targeted metabolomics sequencing to comprehensively define the broader metabolic reprogramming orchestrated by BACH1. The results revealed that modulating BACH1 expression led to extensive alterations in cellular metabolic patterns. Specifically, knockout of BACH1 inhibited the expression of genes involved in key anabolic pathways, including amino acid synthesis, transport, and fatty acid synthesis in HepG2 cells. These findings indicate that the role of BACH1 extends beyond glycolytic amplification to coordinate a comprehensive pro-tumor metabolic network, simultaneously fueling energy production and biosynthetic precursor supply to support invasion and metastasis.

Several limitations warrant consideration. To begin with, our study revealed that antioxidants stimulated BACH1-dependent HCC metastasis. Therefore, pro-oxidants might accelerate free heme release and thus induce BACH1 protein degradation. However, whether pro-oxidants can inhibit metastasis to achieve the purpose of treating HCC requires extensive preclinical studies for verification. Moreover, angiogenesis is an essential component of the metastasis pathway, these new blood vessels are the major conduits for tumor cells to leave original site and enter the circulation [66]. In lung cancer, antioxidants enhanced tumor angiogenesis in a BACH1-dependent manner. BACH1-induced increases in angiogenesis and glycolysis contributed to antioxidant-accelerated lung tumor metastasis [67]. Nevertheless, it remains to be further explored whether BACH1-dependent HCC metastasis facilitated by antioxidants is associated with angiogenesis. It is important to acknowledge the methodological considerations of this work. Our study primarily utilized the tail vein injection model to investigate tumor cell colonization and outgrowth in a distant organ. It should be noted that this approach models experimental metastasis, wherein tumor cells are introduced directly into the venous circulation. This bypasses several critical early steps of the metastatic cascade, such as local invasion, intravasation, and survival in the bloodstream. Consequently, our findings are focused on the later stages of metastasis—specifically, the adaptation and proliferation of tumor cells within the lung microenvironment. Furthermore, as intravenously injected cells are mechanically trapped in the pulmonary capillary bed, this model predominantly examines lung colonization, thereby limiting insights into organ-specific tropism. Future studies employing orthotopic spontaneous metastasis models will be essential to validate the functional relevance of these mechanisms within the context of the complete metastatic cascade.

In summary, the transcription factor BACH1 accelerated HCC progression by promoting metastasis of HCC cells. Targeting BACH1 and its downstream genes might be a potential avenue for clinical treatment of HCC. Antioxidants stimulated BACH1-dependent HCC metastasis. This study provided a theoretical reference for exploring new anti-tumor molecular targets and the utilization of antioxidants in HCC therapy.

## Supplementary information

supplementary information

original Western blots

## Data Availability

The datasets generated during the current study are available from the corresponding author upon reasonable request.
