# Supplementary material for: BACH1 stabilization by antioxidants facilitates hepatocellular carcinoma metastasis
Source: Cell Death Dis. 2026 Jun 9;17(1):725. doi: 10.1038/s41419-026-08865-0 (PMC13473626; doi:10.1038/s41419-026-08865-0)
Supplement: Supplementary file 1 [file 41419_2026_8865_MOESM1_ESM.pdf]

BACH1 stabilization by antioxidants facilitates hepatocellular carcinoma metastasis

Qiqi Bu[1,#], Zhanhang Wang[2,#], Qing Wang[3], Xiaojing Qi[1], Shanshan Lin[1], Shujuan Liu[2], Yiguo Zhang[1,4,*]

[1]The Laboratory of Cell Biochemistry and Topogenetic Regulation, College of Bioengineering, Chongqing University, Chongqing 400044, China

[2]College of Animal Science and Technology, Northwest A&F University, Yangling, Shaanxi 712100, China

[3]Yu-Yue Pathology Scientific Research Center, 313 Gaoteng Avenue, Jiulongpo District, Chongqing 400039, China

[4]Department of Laboratory Medicine, Chongqing Center for Clinical Laboratory, Chongqing Academy of Medical Sciences, Chongqing General Hospital, School of Medicine, Chongqing University, Chongqing 401147, China

# These authors contributed equally to this work.

*Corresponding author: yiguozhang@cqu.edu.cn

Methods

1.1. Immunohistochemistry (IHC)

In brief, the fixed mice tissues were embedded in paraffin and made into 5 µm sections. After deparaffinized with xylene and rehydrated with graded ethanol, the sections were incubated with 3% hydrogen peroxide for 10 min to block endogenous peroxidase. Subsequently, sections were microwaved in 0.01M citrate buffer (PH 6.0) for 20 min at 95°C and blocked with BSA for 30 min after cooling. Incubations were performed with primary antibodie against E-cadherin (20874-1-AP, Proteintech Group), Vimentin (60330-1-Ig, Proteintech Group) overnight at 4°C and then HRP-conjugated secondary antibodie at room temperature for 1 h. After stained with DAB, counterstained with hematoxylin, sections were dehydrated rapidly and sealed with neutral resin. Images were captured with confocal microscopy (Olympus).

1.2. Real-time quantitative PCR

Total RNAs of experimental cells were extracted with the RNA simple kit (Tiangen, Beijing, China) and added the premixed reverse transcription reagent (PrimeScript™ RT Master Mix, RR036A, Takara, JPN) to obtain the cDNAs.

The real-time PCR reaction mixture (20μL) including GoTaq qPCR Master Mix (Promega, USA), primers (synthesized by Tsingke, Chengdu, China and listed in Table S1) and cDNA templates were incubated at 95°C for 5 min, followed by 40 cycles at 95°C for 15 s and extending at 60°C for 30 s, in the CFX Connect Real-Time PCR Detection System (Bio-Rad, CA, USA). Therein, β-actin was used as an internal control for normalization. The $2^{-\triangle\triangle}$Ct method was used to calculate the relative mRNA expression abundances.

1.3. Western blotting with distinct antibodies

The experimental cells were lysed in the RIPA buffer (R0010, Solarbio, Beijing, China) supplemented with the protease and Protein Phosphatase inhibitor (P6730, P1260, Solarbio). The lysates were diluted with 5 × SDS-PAGE loading buffer (P1040, Solarbio), denatured at 100°C for 10 min. Equal amounts of protein extracts were loaded in each well of SDS-PAGE gels containing 8% (or 10%, 12%) polyacrylamide, and transferred to the polyvinylidene fluoride membranes (Millipore Co., Tullagreen, Ireland).

1.4. Luciferase reporter assay

The ARE and adjacent sequences contained in the HK1, GAPDH, PFKL or GLUT4 promoter were inserted into the pGL3-promoter vector to construct ARE reporter gene plasmids. To generate a mutant version lacking the predicted BACH1 binding site, site-directed mutagenesis was performed on the HK1 (or GAPDH/GLUT4) -luciferase reporter plasmid using a Site-Directed Mutagenesis Kit (Beyotime Biotechnology, Shanghai, China). BACH1 expression plasmid was constructed as described in 2.1. HEK293 or HepG2 cells were seeded in 12-well plate and cultured overnight. When the cells density reached 70%-80%, they were co-transfected with BACH1 expression plasmids, reporter gene plasmids and Renilla luciferase reporter plasmid (pRL-TK) for 8 h. After being incubated in complete medium for 24 h, the cells were lysed and dual-reporter assay (Promega, USA) was used to assay the

luciferase activities. The ratio of Firefly/Renilla luciferase activity was calculated.

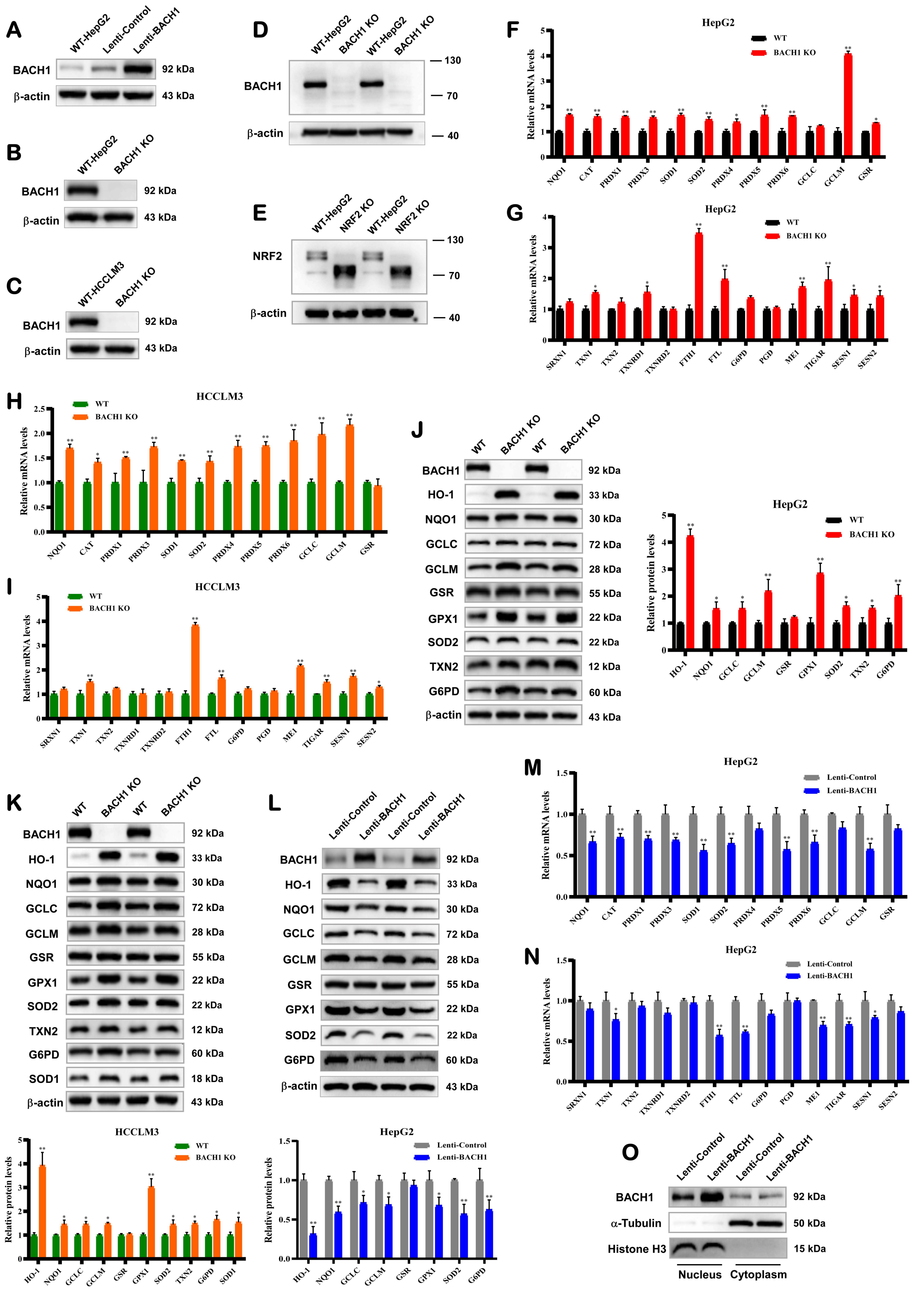

Fig. S1. Validation of BACH1 stable overexpression and knockout cell lines, as well as the specificity of antibodies against BACH1 and NRF2.

(A) Detection of BACH1 levels in wild-type HepG2, Lenti-Control and Lenti-BACH1 cells by

Western blotting. (B) Detection of BACH1 levels in wild-type HepG2 and HepG2 BACH1-KO cells by Western blotting. (C) Detection of BACH1 levels in wild-type HCCLM3 and HCCLM3 BACH1-KO cells by Western blotting. (D) Validation of the BACH1 antibody using extracts from wild-type HepG2 and HepG2 BACH1-KO cells. (E) Validation of the NRF2 antibody using extracts from wild-type HepG2 and HepG2 NRF2-KO cells. (F, G) Detection of NQO1, CAT, PRDX1, PRDX3, SOD1, SOD2, PRDX4, PRDX5, PRDX6, GCLC, GCLM, GSR, SRXN1, TXN1, TXN2, TXNRD1, TXNRD2, FTH1, FTL, G6PD, PGD, ME1, TIGAR, SESN1 and SESN2 levels in HepG2 and HepG2 BACH1-KO cells by RT-qPCR ($n = 3 \times 3$). (H, I) Detection of NQO1, CAT, PRDX1, PRDX3, SOD1, SOD2, PRDX4, PRDX5, PRDX6, GCLC, GCLM, GSR, SRXN1, TXN1, TXN2, TXNRD1, TXNRD2, FTH1, FTL, G6PD, PGD, ME1, TIGAR, SESN1 and SESN2 levels in HCCLM3 and HCCLM3 BACH1-KO cells by RT-qPCR ($n = 3 \times 3$). (J) Western blotting analysis and quantification of HO-1, NQO1, GCLC, GCLM, GSR, GPX1, SOD2, TXN2 and G6PD levels in HepG2 and HepG2 BACH1-KO cells ($n = 3$). (K) Western blotting analysis and quantification of HO-1, NQO1, GCLC, GCLM, GSR, GPX1, SOD2, TXN2, G6PD and SOD1 levels in HCCLM3 and HCCLM3 BACH1-KO cells ($n = 3$). (L) Western blotting analysis and quantification of HO-1, NQO1, GCLC, GCLM, GSR, GPX1, SOD2, and G6PD levels in Lenti-Control and Lenti-BACH1 cells ($n = 3$). (M, N) Detection of NQO1, CAT, PRDX1, PRDX3, SOD1, SOD2, PRDX4, PRDX5, PRDX6, GCLC, GCLM, GSR, SRXN1, TXN1, TXN2, TXNRD1, TXNRD2, FTH1, FTL, G6PD, PGD, ME1, TIGAR, SESN1 and SESN2 levels in Lenti-Control and Lenti-BACH1 cells by RT-qPCR ($n = 3 \times 3$). (O) Subcellular localization of BACH1 in Lenti-Control and Lenti-BACH1 cells. Data are presented as mean ± SD (*$p < 0.05$, **$p < 0.01$).

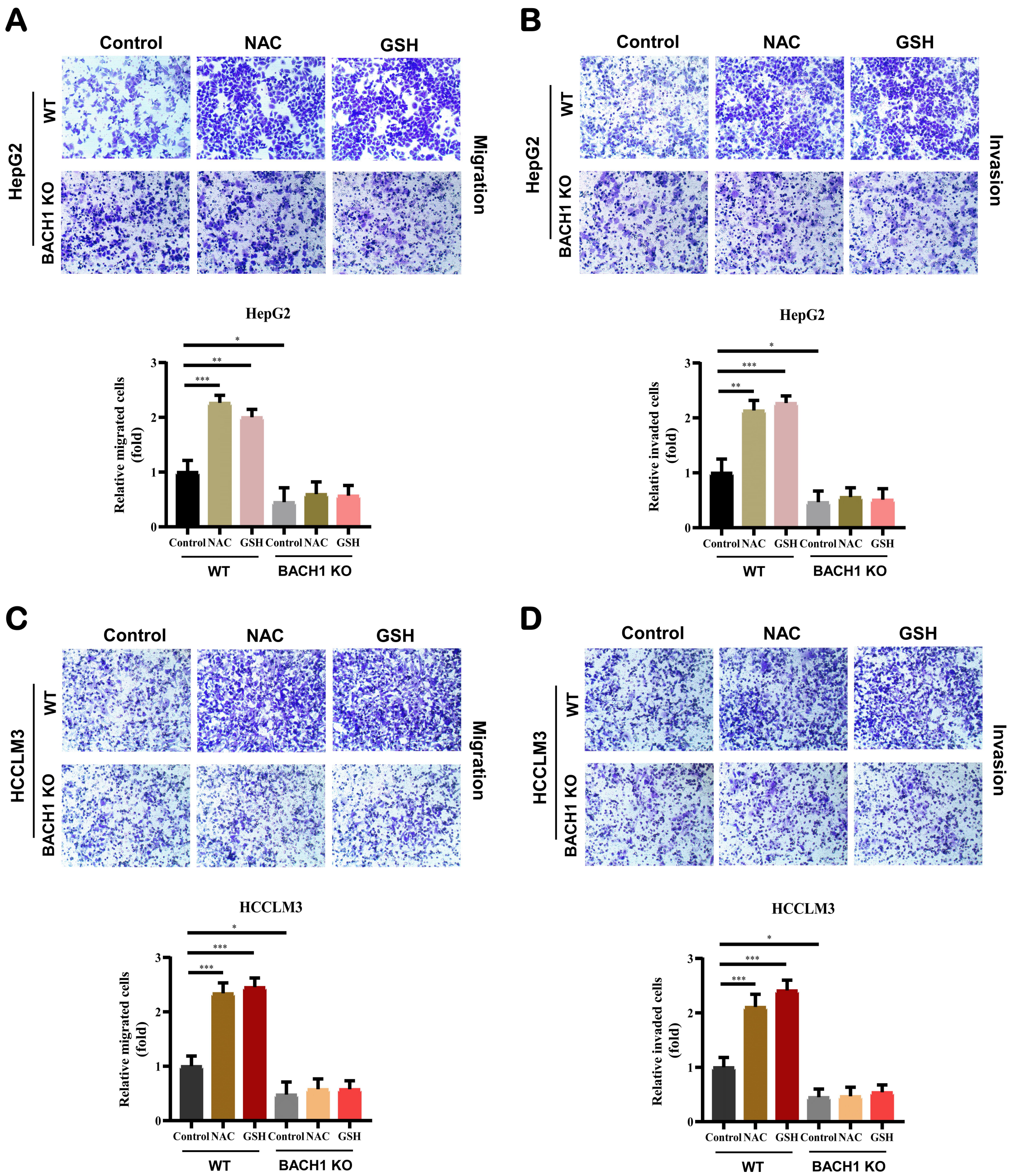

Fig. S2. Antioxidants NAC and GSH facilitated BACH1-dependent migration and invasion of HCC cells in vitro.

(A, B) Transwell migration (A) and invasion (B) assays of HepG2 and HepG2 BACH1-KO cells after treatment with control, 5 mM NAC and 2 mM GSH for 24 h, respectively. Up, representative images of migrated and invaded cells. (C, D) Transwell migration (C) and invasion (D) assays of HCCLM3 and HCCLM3 BACH1-KO cells after treatment with control, 5 mM NAC and 2 mM GSH for 24 h, respectively. Up, representative images of migrated and invaded cells. Data are expressed with mean ± SD (n = 3, *p < 0.05, **p < 0.01, ***p < 0.001).

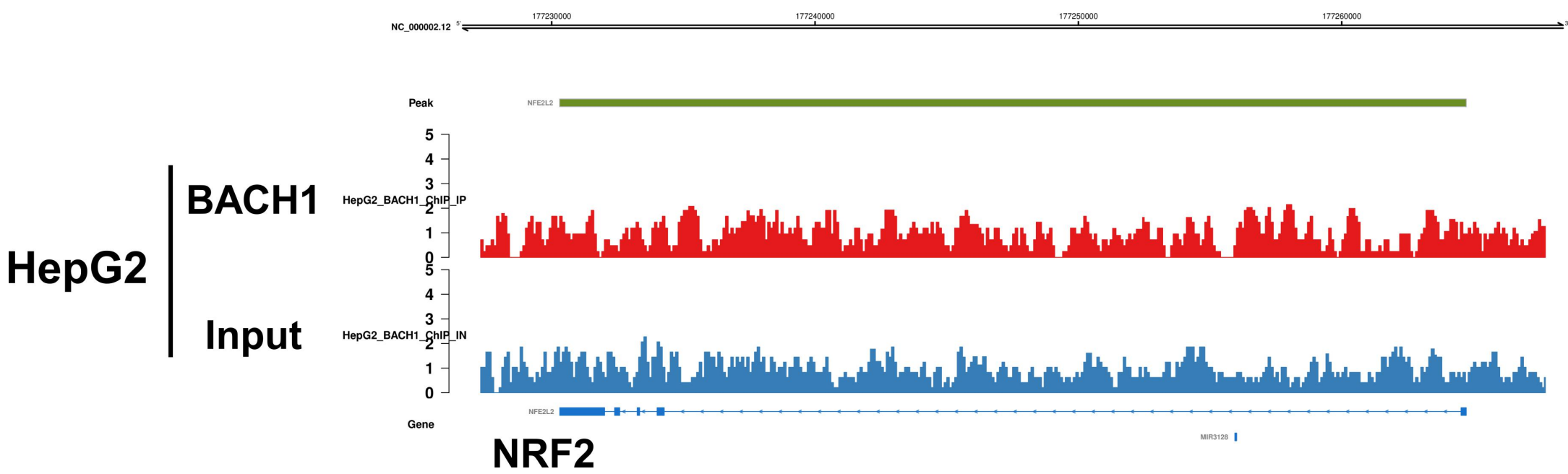

Fig. S3. BACH1 ChIP-seq analysis shows no significant enrichment at the NRF2 genomic region. ChIP-seq analysis was performed in HepG2 cells using an antibody against BACH1. The red tracks represent the normalized read density from the BACH1 ChIP sample, while the blue tracks represent the Input control. The green bar indicates the gene body, with the direction of transcription indicated by the arrow. No significant binding peak was detected at the NRF2 genomic region.

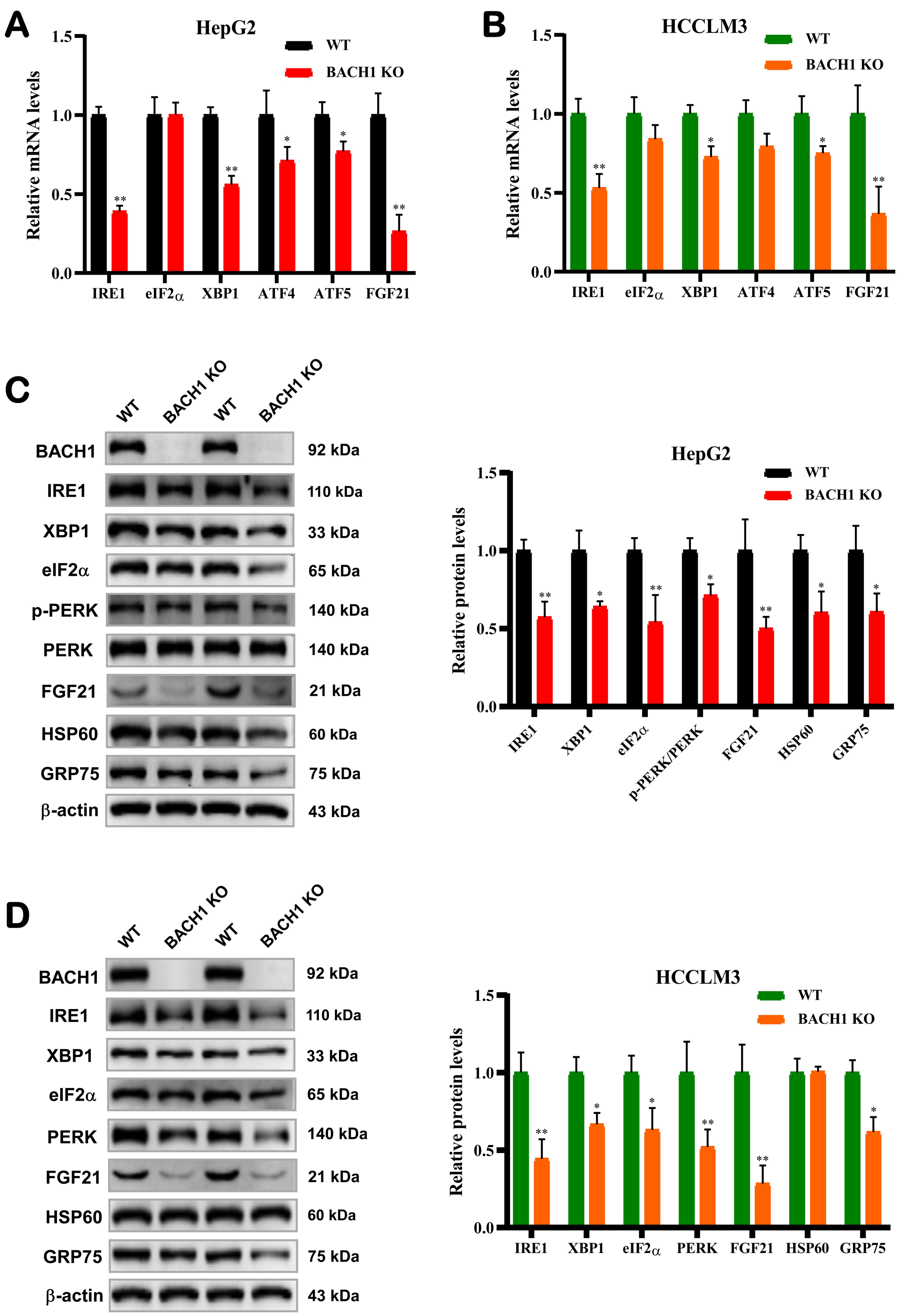

Fig. S4. Knockout of BACH1 inhibits UPR and UPR^{mt} pathways.

(A) Detection of IRE1, eIF2α, XBP1, ATF4, ATF5 and FGF21 levels in HepG2 and HepG2 BACH1-KO cells by RT-qPCR (n = 3 × 3). (B) Detection of IRE1, eIF2α, XBP1, ATF4, ATF5 and FGF21 levels in HCCLM3 and HCCLM3 BACH1-KO cells by RT-qPCR (n = 3 × 3). (C) Western blotting analysis and quantification of IRE1, XBP1, eIF2α, p-PERK/PERK, FGF21, HSP60 and GRP75 levels in HepG2 and

HepG2 BACH1-KO cells (n = 3). (D) Western blotting analysis and quantification of IRE1, XBP1, eIF2α, PERK, FGF21, HSP60 and GRP75 levels in HCCLM3 and HCCLM3 BACH1-KO cells (n = 3). Data are showed as mean ± SD (*$p < 0.05$, **$p < 0.01$).

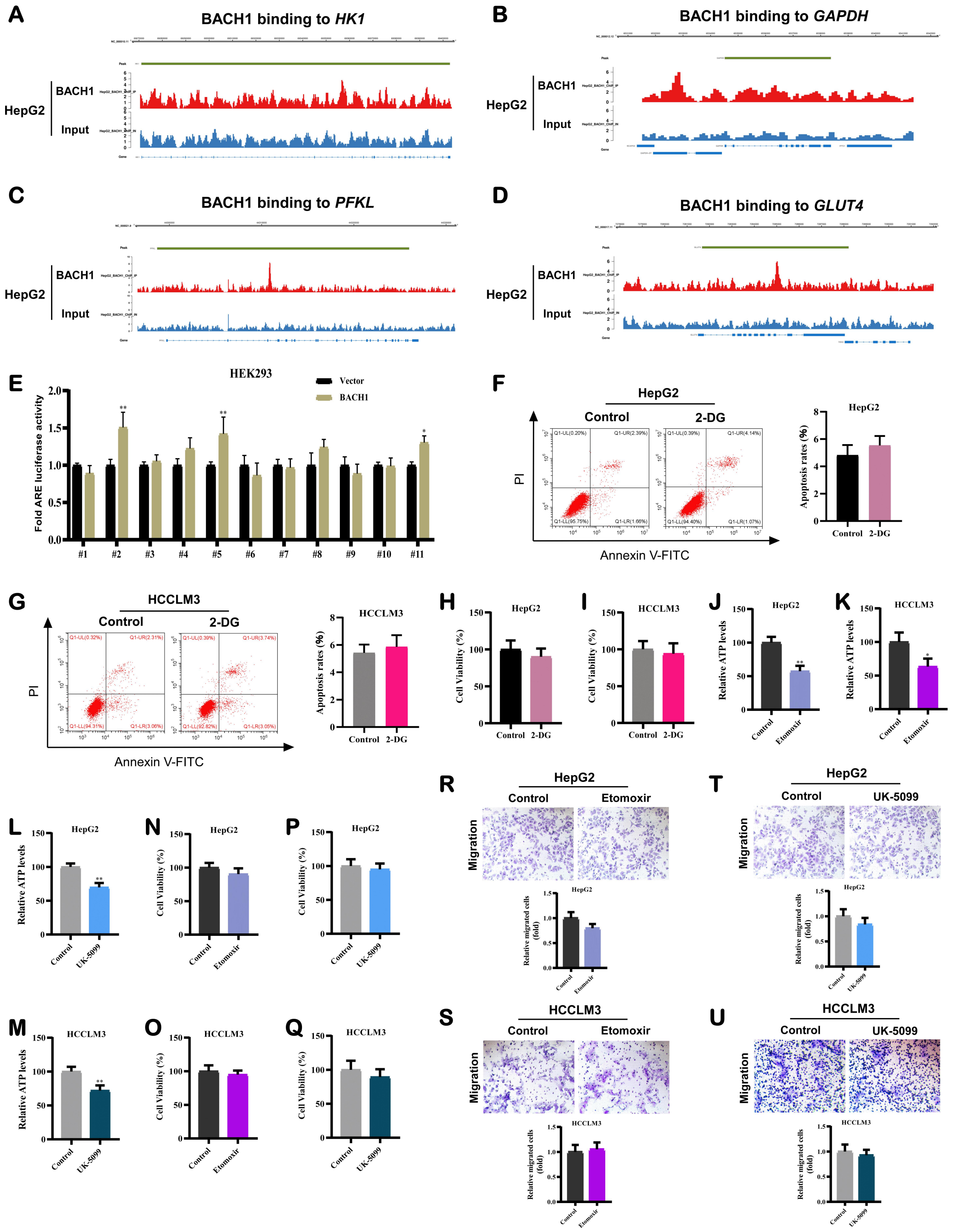

Fig. S5. Glycolysis specifically mediates the promotion of BACH1 on invasion and migration of HCC cells.

(A-D) ChIP-seq analysis was performed in HepG2 cells using an antibody against BACH1 to assess its binding to HK1 (A), GAPDH (B), PFKL (C), and GLUT4 (D). The red tracks represent the normalized read density from the BACH1 ChIP sample, while the blue tracks represent the Input control. The green bar at the top of each panel indicates the gene body, with the direction of transcription indicated by the arrow. (E) The effects of BACH1 on luciferase activation at AREs of HK1, GAPDH, PFKL and GLUT4 promoter in HEK293 cells (n = 3 × 3). (F, G) Cell apoptosis of HepG2 (F) and HCCLM3 (G) cells were analyzed by flow cytometry after treatment with control or 5 mM

2-DG for 24 h (n = 3). (H, I) Cell viability of HepG2 (H) and HCCLM3 (I) cells were detected by CCK8 after treatment with control or 5 mM 2-DG for 24 h (n = 3 × 3). (J, K) ATP levels in HepG2 (J) and HCCLM3 (K) cells after treatment with control or 100μM Etomoxir for 24 h (n = 3 × 3). (L, M) ATP levels in HepG2 (L) and HCCLM3 (M) cells after treatment with control or 1μM UK-5099 for 24 h (n = 3 × 3). (N, O) Cell viability of HepG2 (N) and HCCLM3 (O) cells were detected by CCK8 after treatment with control or 100μM Etomoxir for 24 h (n = 3 × 3). (P, Q) Cell viability of HepG2 (P) and HCCLM3 (Q) cells were detected by CCK8 after treatment with control or 1μM UK-5099 for 24 h (n = 3 × 3). (R, S) Transwell migration assays of HepG2 (R) and HCCLM3 (S) cells after treatment with control or 100μM Etomoxir for 24 h. Up, representative images of migrated cells (n = 3). (T, U) Transwell migration assays of HepG2 (T) and HCCLM3 (U) cells after treatment with control or 1μM UK-5099 for 24 h. Up, representative images of migrated cells (n = 3). Data are showed as mean ± SD (*p < 0.05, **p < 0.01).

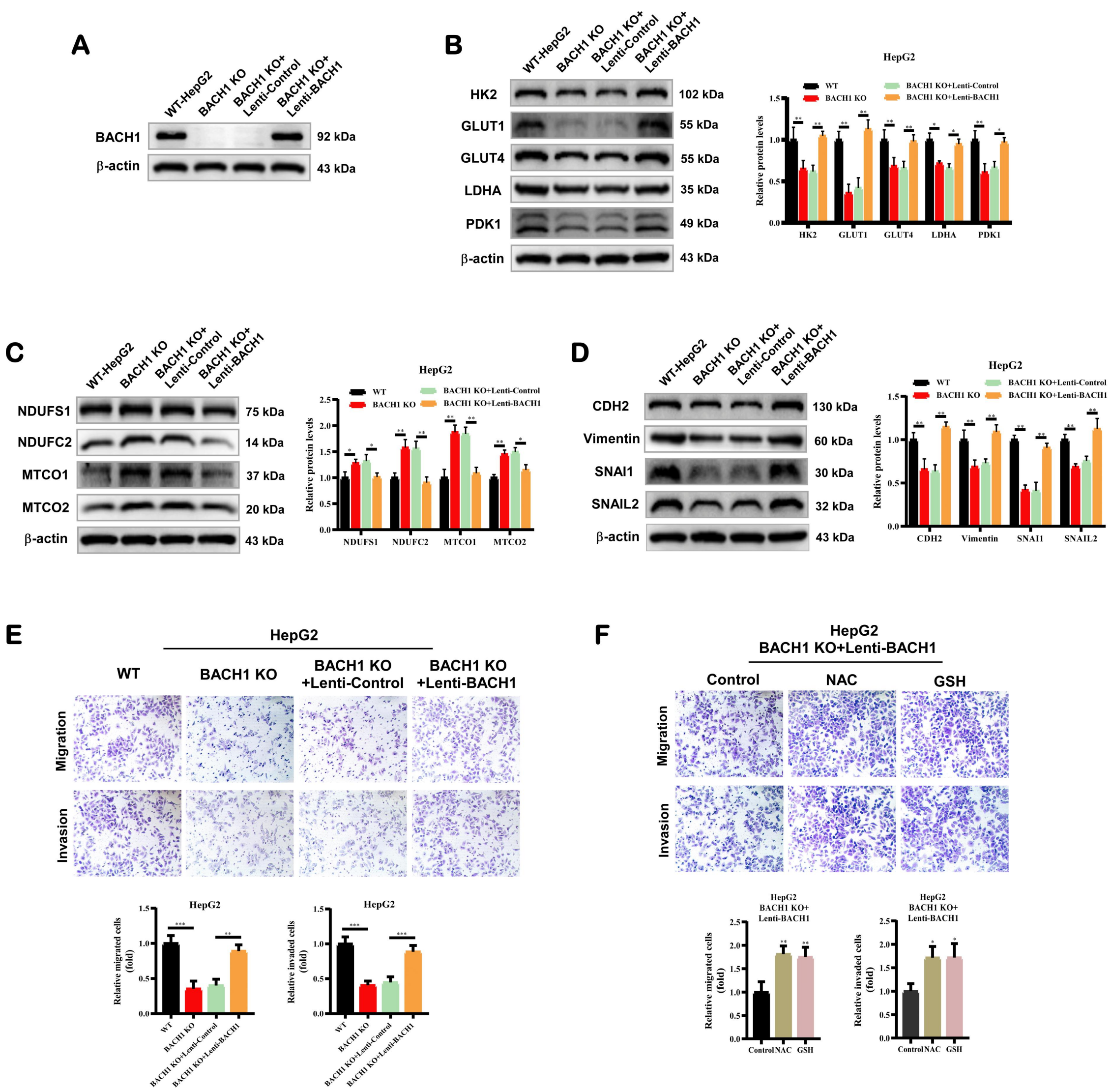

Fig. S6. Reconstitution of BACH1 reverses the metabolic and migratory phenotypes of HepG2 BACH1-KO cells.

(A) Detection of BACH1 levels in wild-type HepG2, HepG2 BACH1-KO, BACH1-KO+Lenti-Control and BACH1-KO+Lenti-BACH1 cells by Western blotting. (B)Western blotting analysis and quantification of HK2, GLUT1, GLUT4, LDHA and PDK1 levels in wild-type HepG2, HepG2 BACH1-KO, BACH1-KO+Lenti-Control and BACH1-KO+Lenti-BACH1 cells (n = 3). (C) Western blotting analysis and quantification of NDUFS1, NDUFC2, MTCO1 and MTCO2 levels in wild-type HepG2, HepG2 BACH1-KO, BACH1-KO+Lenti-Control and BACH1-KO+Lenti-BACH1 cells (n = 3). (D) Western blotting analysis and quantification of CDH2, Vimentin, SNAI1 and SNAIL2 levels in wild-type HepG2, HepG2 BACH1-KO, BACH1-KO+Lenti-Control and BACH1-KO+Lenti-BACH1 cells (n = 3). (E) Transwell invasion and migration assays of wild-type HepG2, HepG2 BACH1-KO, BACH1-KO+Lenti-Control and BACH1-KO+Lenti-BACH1 cells. Up, representative images of migrated and invaded cells (n = 3). (F) Transwell migration and invasion assays of HepG2 BACH1-KO+Lenti-BACH1 cells after treatment with control, 5 mM NAC or 2 mM GSH for 24 h, respectively. Up, representative images of migrated and invaded cells (n = 3). Data are showed as mean ± SD (*p < 0.05, **p < 0.01, ***p < 0.001).

Table S1. The primer pairs used for quantitative RT-PCR

Genes	Nucleotide sequences (5'→ 3')
ACTB	FP: CCAACCGCGAGAAGATGACC
	RP: GAGTCCATCACGATGCCAGT
NRF2	FP: TGTGGCATCACCAGAACACTC
	RP: TCCAGGGGCACTATCTAGCTC
HO-1	FP: CCAGGCAGAGAATGCTGAGTTC
	RP: AAGACTGGGCTCTCCTTGTTGC
NQO1	FP: GCAGACCTTGTGATATTCCAGTTCC
	RP: TGGCAGCGTAAGTGTAAGCAAAC
GSR	FP: CACGAGTGATCCCAAGCCC
	RP: CAATGTAACCTGCACCAACAATG
GCLC	FP: TCAATGGGAAGGAAGGTGTGTT
	RP: TTGTAGTCAGGATGGTTTGCGA
GCLM	FP: TGCTGTGTGATGCCACCAGA
	RP: CGCTTGAATGTCAGGAATGCTT
SOD2	FP: AATAGCTGGGATTACAGGTGCAT
	RP: CCAGACGGATCACTTGAGGTCAG
CDH1	FP: GCTAATTCTGATTCTGCTGCTCTTG
	RP: GTCCTCTTCTCCGCCTCCTTC
CDH2	FP: AGAGTTTACTGCCATGACGTT
	RP: ACTGATTCTGTACACTGCGTTC
Vimentin	FP: CACCAGCCGCAGCCTCTAC
	RP: CGAGAAGTCCACCGAGTCCTG
SNAI1	FP: CCTCGCTGCCAATGCTCATCTG
	RP: AGCCTTTCCCACTGTCCTCATCTG
SNAIL2	FP: GCCTGTCATACCACAACCAGAGATC
	RP: GGAGGAGGTGTCAGATGGAGGAG
TWIST1	FP: CAACAGCGAGGAAGAGCCAGAC
	RP: CCACAGCCCGCAGACTTCTTG
HK1	FP: CACATGGAGTCCGAGGTTTATG
	RP: CGTGAATCCCACAGGTAACTTC
HK2	FP: GAGCCACCACTCACCCTACT
	RP: CCAGGCATTCGGCAATGTG
GLUT1	FP: ATTGGCTCCGGTATCGTCAAC
	RP: GCTCAGATAGGACATCCAGGGTA
GLUT4	FP: TGGGCGGCATGATTTCCTC
	RP: GCCAGGACATTGTTGACCAG
PFKL	FP: GGTGCTGCGGAACGAGAAGTG
	RP: AGACGCCCTTGCCCTCTGATG
GAPDH	FP: ACAACTTTGGTATCGTGGAAGG
	RP: GCCATCACGCCACAGTTTC
LDHA	FP: ATGGCAACTCTAAAGGATCAGC
	RP: CCAACCCCAACAACTGTAATCT
GPI	FP: GACCGCTTCAACCACTTCAG
	RP: CACATCCTTGCCGTCTACCA
PGAM1	FP: TCAATGAGCGGCACTATGGG

Genes	Nucleotide sequences (5'→ 3')
	RP: GCTGTAGAAAGGATGGTCGGG
PGK1	FP: AGCCGAGCCAGCCAAAATA
	RP: TCAAAAACCCACCAGCCTTC
PKM	FP: ATGTCGAAGCCCCATAGTGAA
	RP: TGGGTGGTGAATCAATGTCCA
ALDOA	FP: GGAGGAGTATGTCAAGCGAGC
	RP: CGTGGTTAGAGACGAAGAGGG
PDK1	FP: ATTCAACTGCACCAAGACCT
	RP: TGCATCTGTCCCGTAACCCTC
NDUFS1	FP: TTCCAGCAAGCAAATGAGCTCT
	RP: AGGCTCTGCTAATTGAATCTGT
NDUFC2	FP: ACTCAGGAGCTAATACTGTCT
	RP: GGACAAGCATTTGATAAGCCAT
NDUFV2	FP: TCAGGTCTGCACTACTACACCC
	RP: CTCCCCAACCTTTATTCCAAGC
SDHA	FP: CCTACTTCAGCTGCACGTCT
	RP: ACAACCAGCACCATATATGCC
SDHB	FP: ACCTTCCGAAGATCATGCAG
	RP: CAATCCTTCGGGTGCAAGCTA
SDHC	FP: CCATATAGGCCAGGCTTTAGCA
	RP: GGCCAACAACTCGAAGTGACA
MTCYB	FP: CAGTCCCACCCTCACACGAT
	RP: GGTTGTTTGATCCCGTTTCGT
UQCRFS1	FP: TCCCTACTGTGTAAGCCGTGT
	RP: ACCAGACGTGAAGATAAACCCT
MTCO1	FP: ACCCCATTCTATACCAACACCT
	RP: ATGGGAGATTATTCCGAAGCCT
MTCO2	FP: GCTGTCCCCACATTAGGCTT
	RP: AGATTTCAGAGCATTGACCGTA
COX5A	FP: ACAAAGCAGGACCTCATAAGGA
	RP: CCATGCGGTTTACACTTTGTCA
ACACA	FP: GTTATTTCTCAGAGCTTCCGAAC
	RP: GAATTTCTTCTGCCAGTCCGAT
FASN	FP: GCCTGCCACAACTCCAAGGAC
	RP: TTCCTCAGCTGCTCCACGAAC
SCD1	FP: CACCACATTCTTCATTGATTGC
	RP: TCAGCCACTCTTGTAGTTTCCA
SREBP1	FP: GGAGCCATGGATTGCACTTT
	RP: CAGGAAGGCTTCAAGAGAGG
SREBP2	FP: CGACTCTGACCAGCACCCACA
	RP: GACGCTCAGGACAATCACACC
ACLY	FP: ATCGGTTCAAGTATGCTCGGG
	RP: GACCAAGTTTTCCACGACGTT
CPT1A	FP: TGCAAAGGCGACATCAATCCG
	RP: AAATCCACGTCGTTTGCCAGA
ASNS	FP: CTTGGTTGCTGCCACTCTGTT
	RP: TCACTCTCCTCCTCGGCTTTT
GPT2	FP: GCTCTTTCTCCTGGCTGATG

Genes	Nucleotide sequences (5′→ 3′)
	RP: CCTCCTCTGTAACCACACTCG
GOT1	FP: TCAAGGAGAAGCGGGTAGGA
	RP: AAACCAGCAGCGGAAAACAC
PHGDH	FP: GAAATCTCTCACGGGGGTTG
	RP: GTTCACATCCGCCTGCTTG
PSAT1	FP: ACCTCAACCCAGATGCCTC
	RP: TCACGGACAATCACCACGG
ASS1	FP: ATGTCCAGCAAAGGCTCCGT
	RP: CCAGATGAACTCCTCCACAAACTC
PSPH	FP: TGTCCTCCTGCTGATGCTTT
	RP: ATACCATTTGGCGTTATCCTTG
SHMT2	FP: CCTATGCTCGCCTCATTGACTAC
	RP: TCCACAGCCTTCACCCCTTT
SLC7A5	FP: CCGTGGACTTCGGGAACTATC
	RP: TGTGAACAGGGACCCATTGAC
SLC1A4	FP: TCAGCGACCCTTCCCTCTATG
	RP: TGGTGAAAATCTGTCCTGCGTT
SLC1A5	FP: TGGAGATGGAGGATGTGGGTT
	RP: GAAGCGGTAGGGGTTTTTGC
SLC7A1	FP: TCCCCATCCTGAGCATCTTC
	RP: CAGCATCCACACAGCAAACC
CAT	FP: CTCCAAATTACTACCCCAACAGC
	RP: ATCATTGGCAGTGTTGAATCTCC
PRDX1	FP: TTCACTGACAAACATGGGGAA
	RP: CGCTCACTTCTGCTTGGAGA
PRDX3	FP: ATCTTGCCTGGATAAATACACC
	RP: TGATCTTAGTGCAAGACCAGA
SOD1	FP: TTGCATCATTGGCCGCACACT
	RP: ATTACACCACAAGCCAAACGACT
PRDX4	FP: GTGTGTCCAACTGAAATTATCGC
	RP: ATTAATCCAGGCCAAATGGGT
PRDX5	FP: CATGGCCCCAATCAAGGTTCG
	RP: CACTATGCCATCCTGTACCAC
PRDX6	FP: TAGGTTGCTATACCAATGGCTT
	RP: ATTTCAAACAGAACCAGCAG
SRXN1	FP: GCTGTGTAACAAACCATCCCCAA
	RP: CAGCCCAAATTGCCAACCCAT
TXN1	FP: CCTTGCAAAATGATCAAGCCTT
	RP: TGGTGGCTTCAAGCTTTTCCT
TXN2	FP: TGACCACACAGACCTCGCCAT
	RP: ATGCCCACAAACTTGTCCACCAC
TXNRD1	FP: GGCTCTATGCAGGTTCCACT
	RP: TCAGAAAGGCCACAAGCACCA
TXNRD2	FP: AGATACCCCACGCACATCGAA
	RP: TTTTCCAGGGGATTCCTTCAGC
FTH1	FP: CTACTGGAACTGCACAAACTGG
	RP: CACCCAATTCTTTGATGGCTT
FTL	FP: TGAGCCACTTCTTCCGCGAAT

Genes	Nucleotide sequences (5′→ 3′)
	RP: CGCCACGCTGGTTTTGCATC
G6PD	FP: CGAGGCCGTCACCAAGAAC
	RP: GTAGTGGTCGATGCGGTAGA
PGD	FP: GTGGCCCCACATCAAGACC
	RP: GTCCCCATACTCTATCCCGTT
ME1	FP: GGGTCGGCTTTATCCTCCTT
	RP: ATGCTTCTTTGTTTTGCGGTT
TIGAR	FP: TCACTGCCCTAAAAGTCCTCT
	RP: AAACTTCTGCCCATTCCCTC
SESN1	FP: CCTCAATGCTTAGACGGGCAA
	RP: TTCAGGAGTGCAAACAACAGT
SESN2	FP: CACCCAGACATGCTGTGCTT
	RP: TAGCCATGGTCTTCCCAGGT
IRE1	FP: AGTCTCTGCCCATCAACCTC
	RP: GCATTCCACCGGAGCTCTCG
eIF2α	FP: ACAATCAGGAAACGATAAGCCAT
	RP: TGCCAAATCTGGACTCTTGTCAC
XBP1	FP: CACCCCTCCAGAACATCTCC
	RP: TGTCCAGAATGCCCAACAGG
ATF4	FP: CCCTTCACCTTCTTACAACCTC
	RP: TGCCCAGCTCTAAACTAAAGGA
ATF5	FP: GCCTCTACCTTCATTCCAAACCC
	RP: TTGACCTCCTGGCCTCAAACGA
FGF21	FP: GGAGCTTCTGCATCTATCCCAA
	RP: GTGCCAGATTCCAGTTGTCCA

Table S2. Genes/proteins consistently regulated by BACH1 in HepG2 (KO/Lenti) and HCCLM3 (KO) cell lines

Gene/Protein	HepG2 Lenti	HepG2 KO	HCCLM3 KO	Regulation	Evidence
**NQO1**	**-0.60±0.10 ****	**0.73±0.05 ****	**0.76±0.05 ****	**Suppressed**	**RT-qPCR**
	**-0.77±0.12 ****	**0.54±0.14 ***	**0.53±0.10 ***		**Western blot**
**CAT**	**-0.48±0.10 ****	**0.67±0.10 ****	**0.50±0.08 ***	**Suppressed**	**RT-qPCR**
**PRDX1**	**-0.52±0.07 ****	**0.68±0.10 ****	**0.60±0.16 ****	**Suppressed**	**RT-qPCR**
**PRDX3**	**-0.56±0.10 ****	**0.64±0.11 ****	**0.82±0.22 ****	**Suppressed**	**RT-qPCR**
**SOD1**	**-0.88±0.15 ****	**0.72±0.10 ****	**0.53±0.08 ****	**Suppressed**	**RT-qPCR**
**SOD2**	**-0.65±0.11 ****	**0.56±0.07 ****	**0.51±0.11 ****	**Suppressed**	**RT-qPCR**
	**-0.84±0.18 ****	**0.71±0.09 ***	**0.52±0.11 ***		**Western blot**
**PRDX5**	**-0.84±0.16 ****	**0.71±0.12 ****	**0.80±0.06 ****	**Suppressed**	**RT-qPCR**
**PRDX6**	**-0.63±0.13 ****	**0.70±0.11 ****	**0.88±0.11 ****	**Suppressed**	**RT-qPCR**
**GCLM**	**-0.87±0.14 ****	**2.03±0.14 ****	**1.11±0.05 ****	**Suppressed**	**RT-qPCR**
	**-0.56±0.12 ***	**0.94±0.23 ****	**0.56±0.03 ***		**Western blot**
**TXN1**	**-0.39±0.12 ***	**0.63±0.10 ***	**0.59±0.15 ****	**Suppressed**	**RT-qPCR**
**FTH1**	**-0.82±0.12 ****	**1.80±0.13 ****	**1.95±0.12 ****	**Suppressed**	**RT-qPCR**
**FTL**	**-0.71±0.06 ****	**0.97±0.15 ****	**0.73±0.08 ****	**Suppressed**	**RT-qPCR**
**ME1**	**-0.53±0.06 ****	**0.80±0.16 ****	**1.12±0.11 ****	**Suppressed**	**RT-qPCR**
**TIGAR**	**-0.51±0.08 ****	**0.94±0.21 ****	**0.57±0.07 ****	**Suppressed**	**RT-qPCR**
**SESN1**	**-0.34±0.10 ***	**0.54±0.17 ***	**0.79±0.10 ****	**Suppressed**	**RT-qPCR**
**HO-1**	**-1.72±0.28 ****	**2.04±0.07 ****	**1.96±0.12 ****	**Suppressed**	**Western blot**
**GCLC**	**-0.50±0.12 ***	**0.58±0.14 ***	**0.53±0.07 ***	**Suppressed**	**Western blot**
**GPX1**	**-0.58±0.14 ***	**1.49±0.20 ****	**1.59±0.10 ****	**Suppressed**	**Western blot**
**G6PD**	**-0.70±0.17 ****	**1.00±0.23 ****	**0.71±0.10 ***	**Suppressed**	**Western blot**

Gene/Protein	HepG2 Lenti	HepG2 KO	HCCLM3 KO	Regulation	Evidence
**SNAI1**	**0.49±0.09 ***	**-0.51±0.09 ***	**-0.87±0.19****	**Activated**	**RT-qPCR**
	**1.64±0.21 ****	**-1.07±0.20****	**-1.66±0.25****		**Western blot**
**SNAIL2**	**0.41±0.16 ***	**-1.16±0.14****	**-1.06±0.21****	**Activated**	**RT-qPCR**
	**1.65±0.20 ****	**-0.57±0.10 ***	**-1.56±0.28****		**Western blot**
**GLUT1**	**0.35±0.07 ***	**-0.48±0.07 ***	**-0.38±0.09 ***	**Activated**	**RT-qPCR**
	**0.59±0.19 ***	**-1.07±0.07****	**-0.83±0.14****		**Western blot**
**PDK1**	**0.39±0.18 ***	**-0.45±0.11 ***	**-0.31±0.06 ***	**Activated**	**RT-qPCR**
	**0.83±0.10 ****	**-0.76±0.16****	**-0.58±0.16****		**Western blot**
**GLUT4**	**0.48±0.17 ***	**-0.80±0.14****	**-0.49±0.11 ***	**Activated**	**Western blot**
**GAPDH**	**0.49±0.08 ***	**-0.37±0.09 ***	**-0.43±0.12 ***	**Activated**	**Western blot**

*Values represent Log2 Fold Change (mean ± SE), *p < 0.05, **p < 0.01.
