# Supplementary material for: BACH1 stabilization by antioxidants facilitates hepatocellular carcinoma metastasis
Source: Cell Death Dis. 2026 Jun 9;17(1):725. doi: 10.1038/s41419-026-08865-0 (PMC13473626; doi:10.1038/s41419-026-08865-0)

Figure 1G

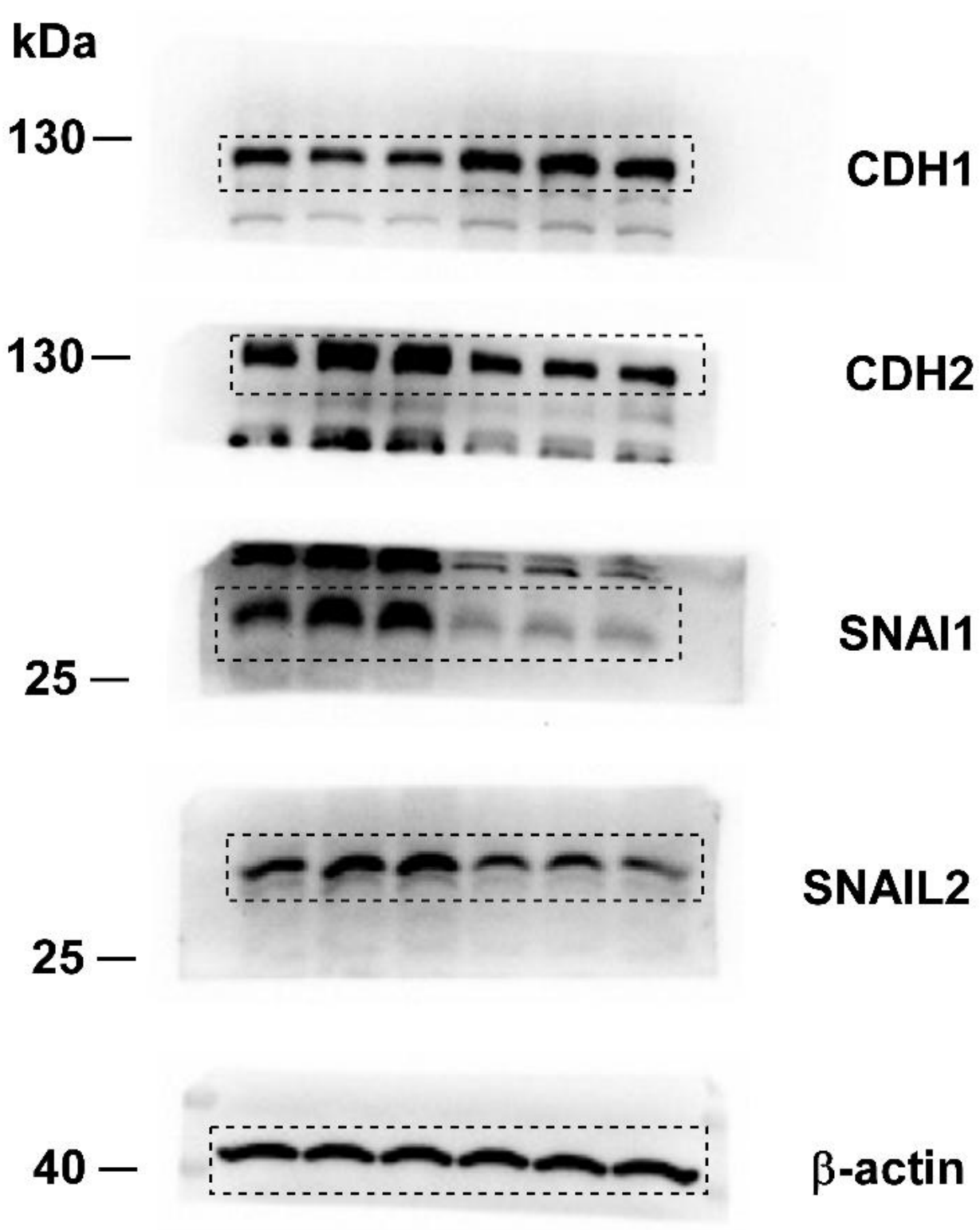

Figure 2J

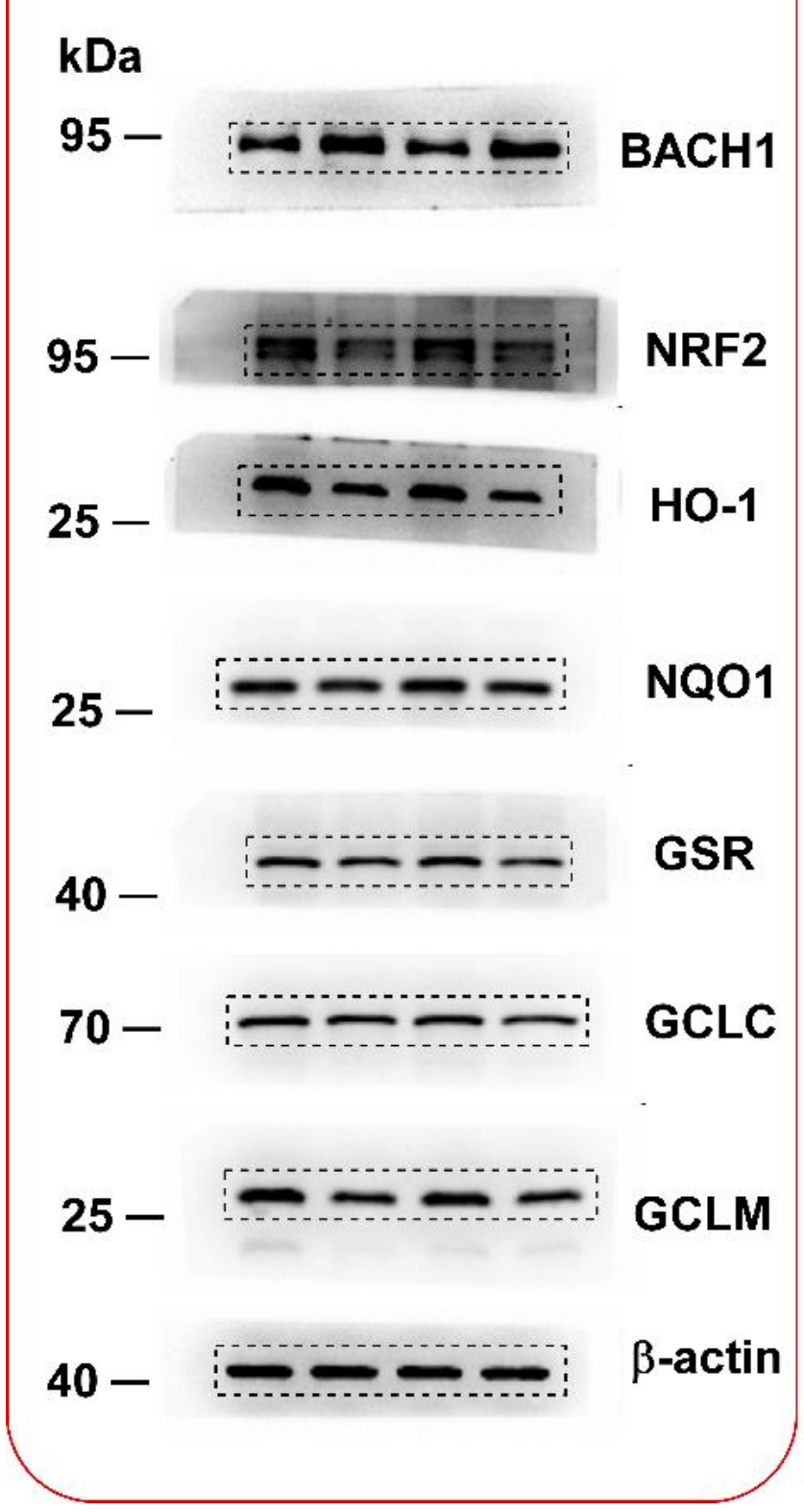

Figure 2K

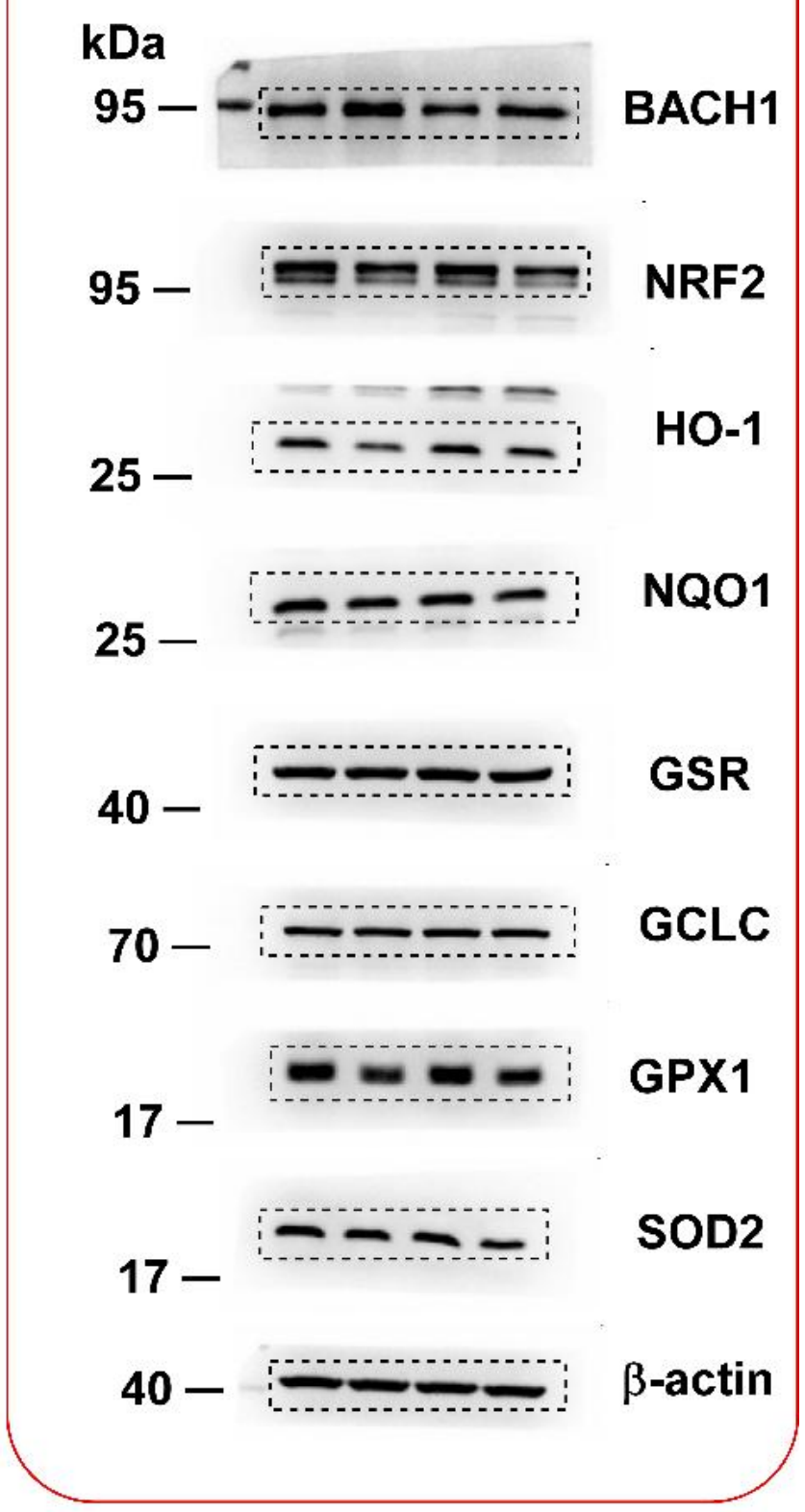

Figure 2L

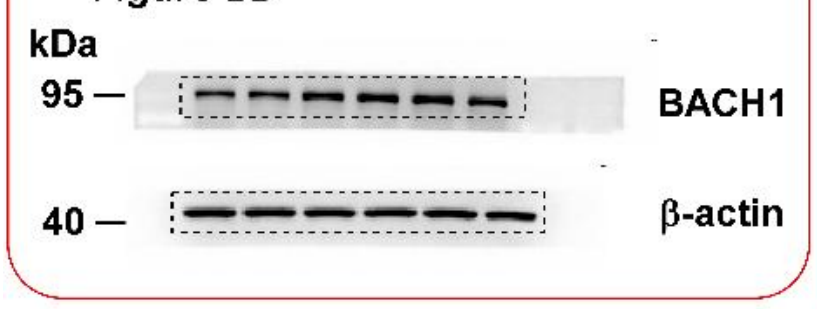

Figure 2M

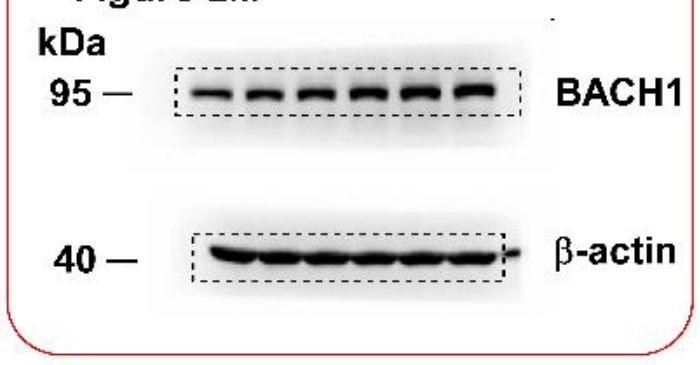

Figure 2N

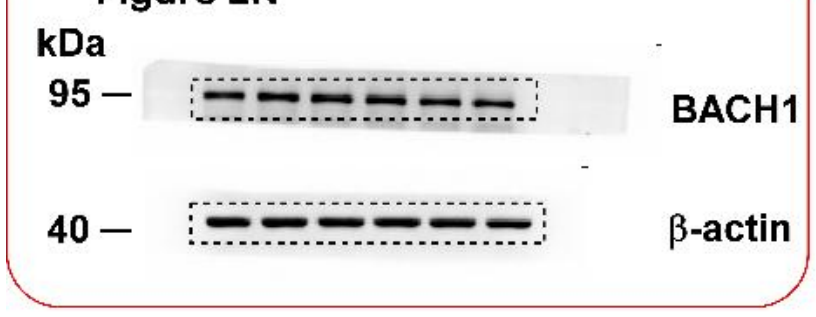

Figure 2O

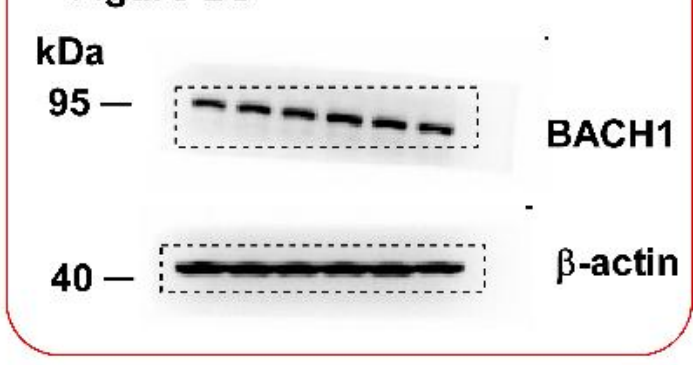

Figure 2P

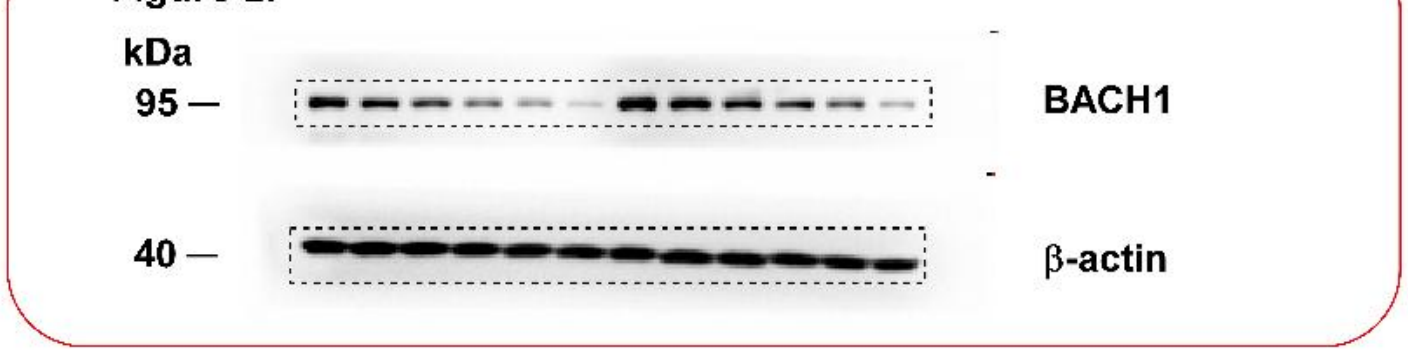

Figure 2Q

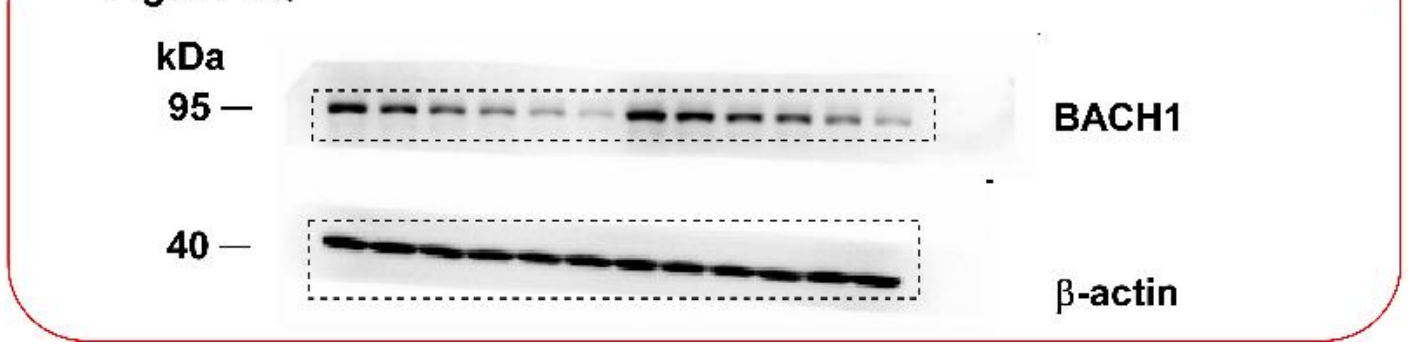

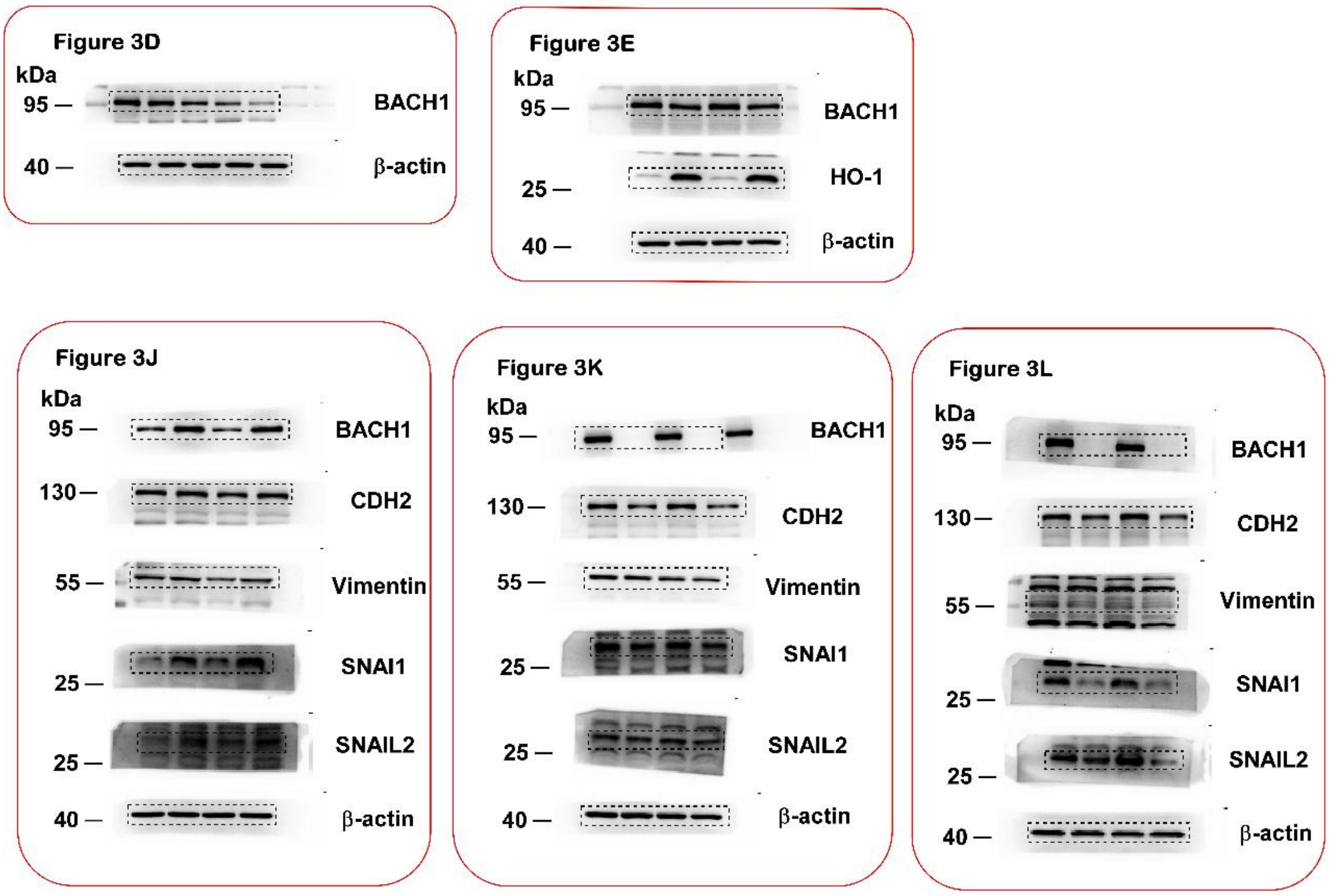
Figure 3D
kDa
95 —
BACH1
40 —
β-actin
Figure 3E
kDa
95 —
BACH1
HO-1
25 —
40 —
β-actin
Figure 3J
kDa
95 —
BACH1
130—
CDH2
55 —
Vimentin
SNAI1
25 —
SNAIL2
25 —
40 —
β-actin
Figure 3K
kDa
95 —
BACH1
130 —
CDH2
55 —
Vimentin
25 —
SNAI1
25 —
SNAIL2
40 —
β-actin
Figure 3L
kDa
95 —
BACH1
130 —
CDH2
55 —
Vimentin
SNAI1
25 —
SNAIL2
25 —
40 —
β-actin

Figure 4E

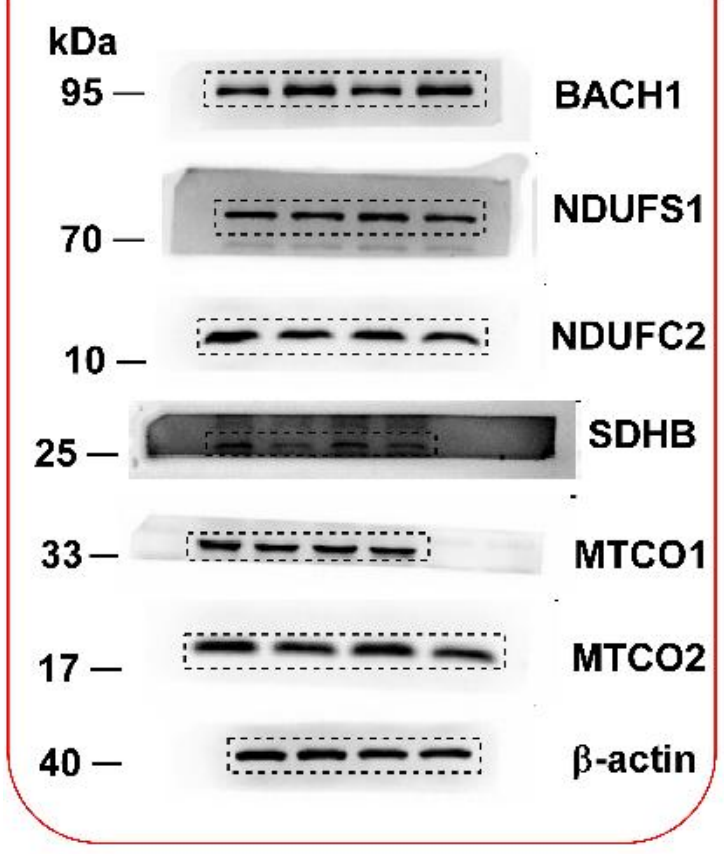

Figure 4F

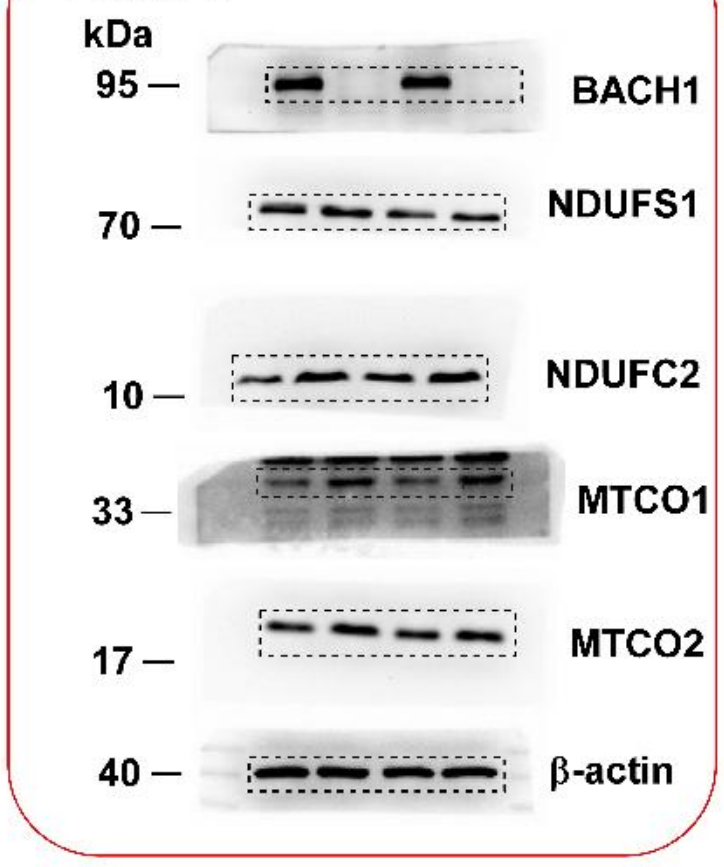

Figure 4N

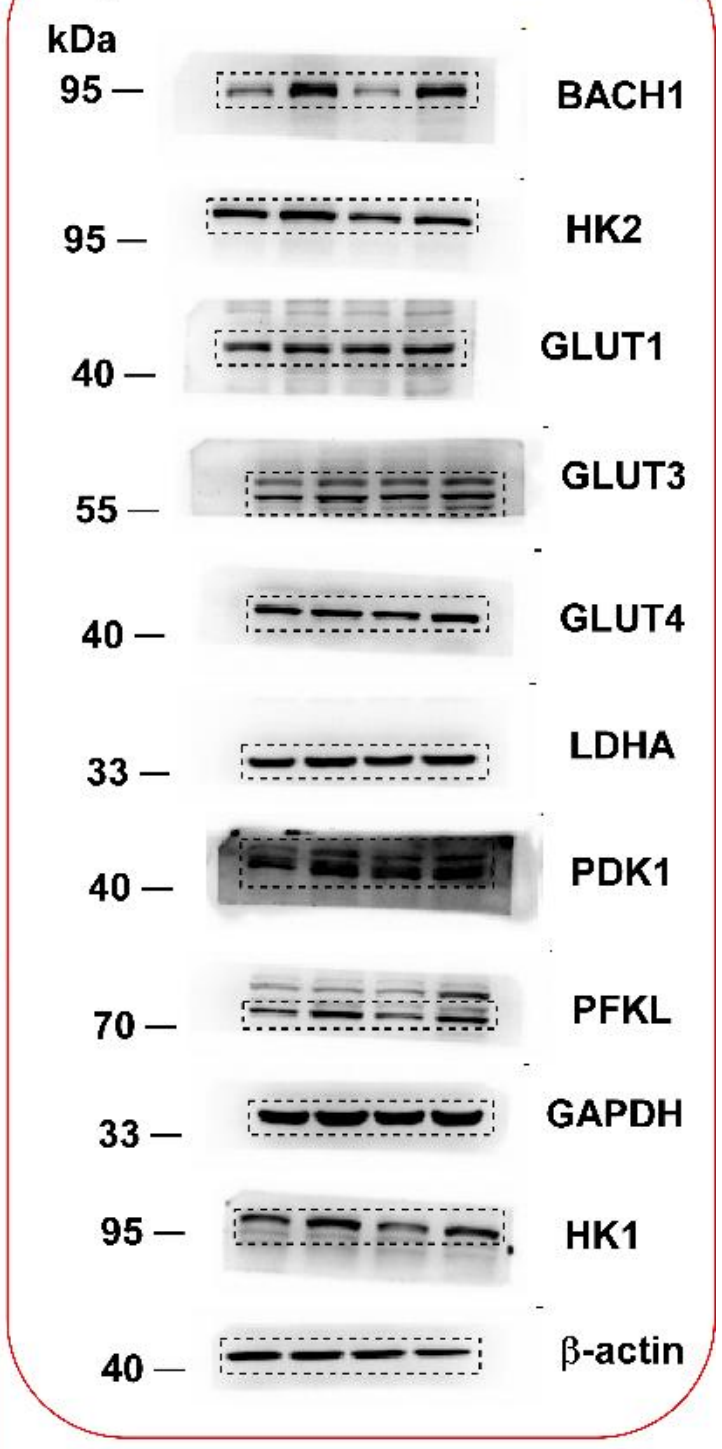

Figure 4O

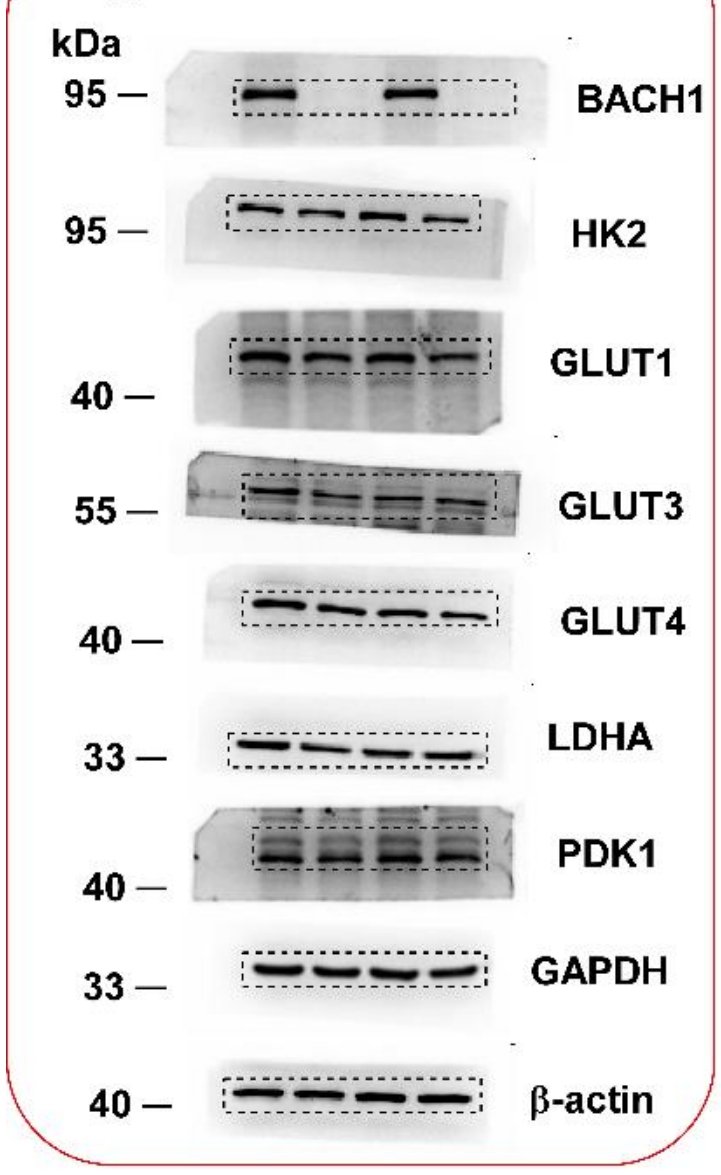

Figure 4P

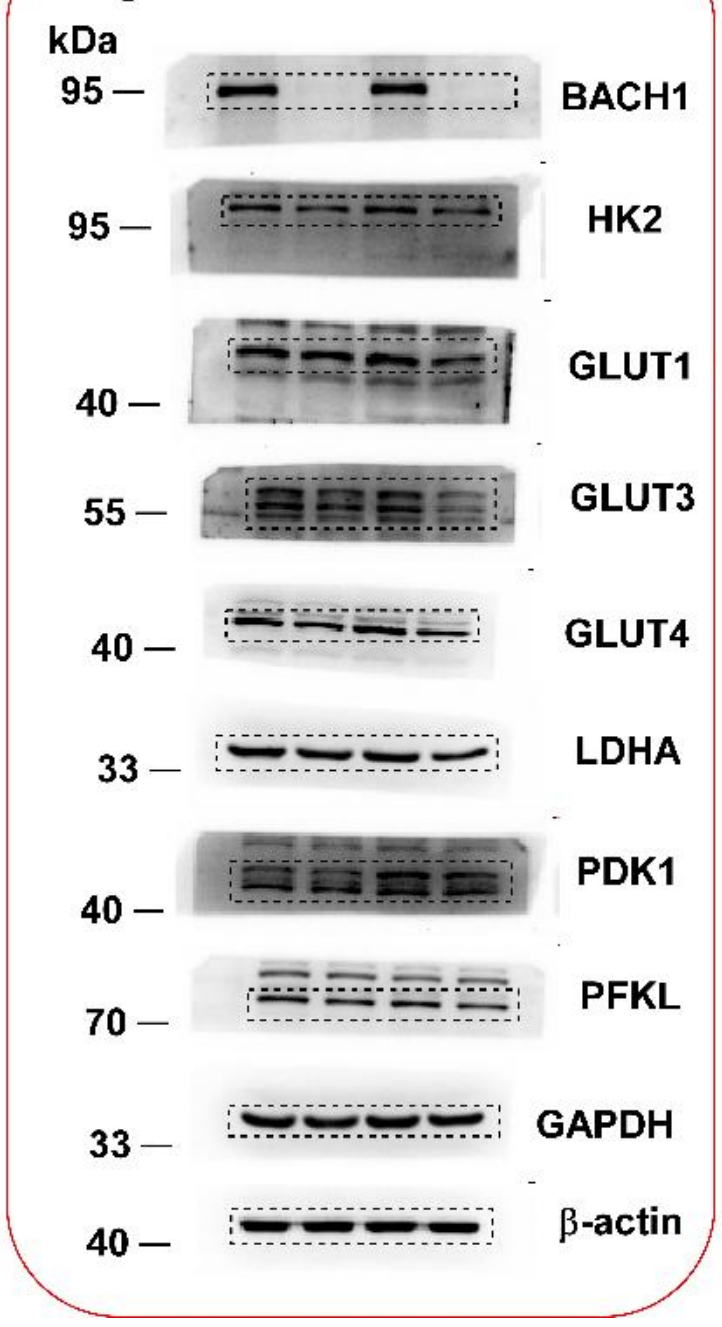

Figure S1A

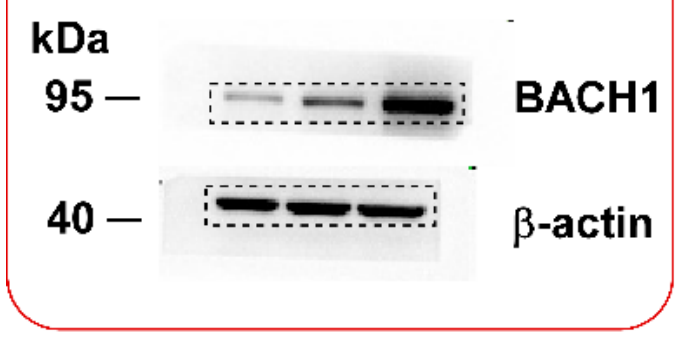

Figure S1B

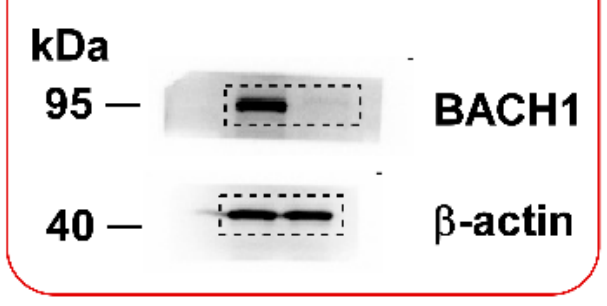

Figure S1C

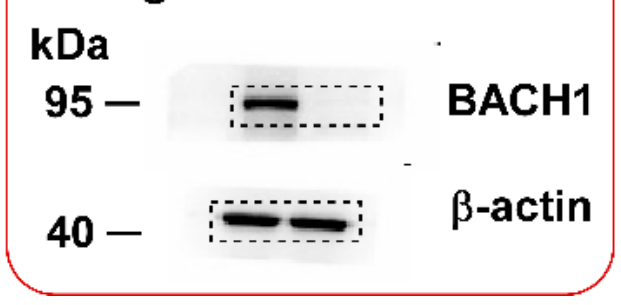

Figure S1D

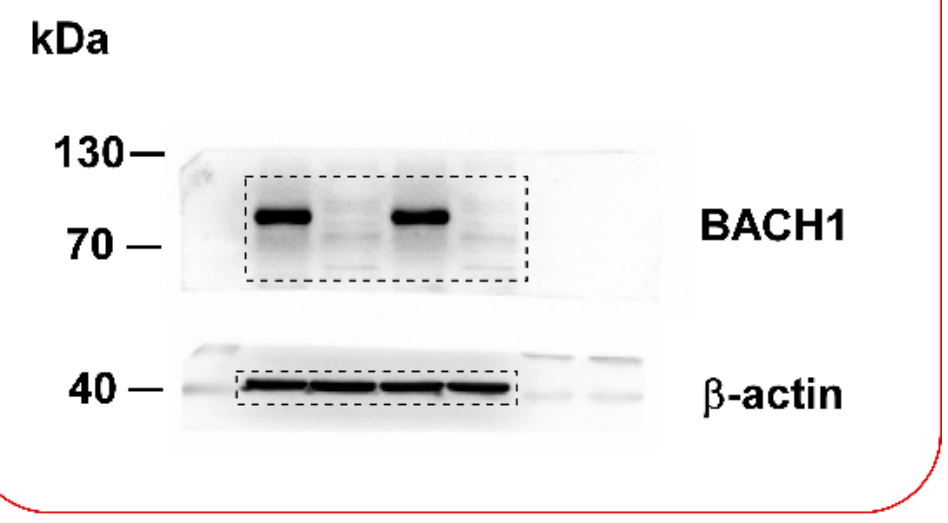

Figure S1E

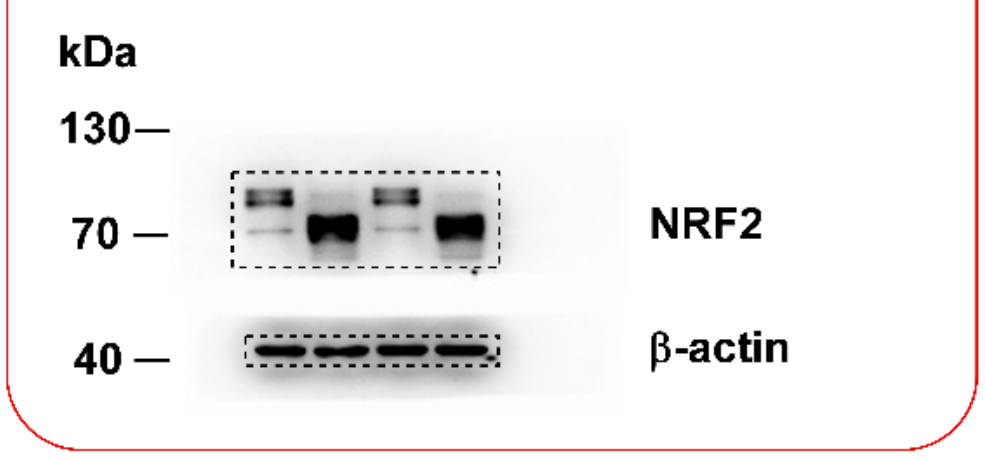

Figure S1J

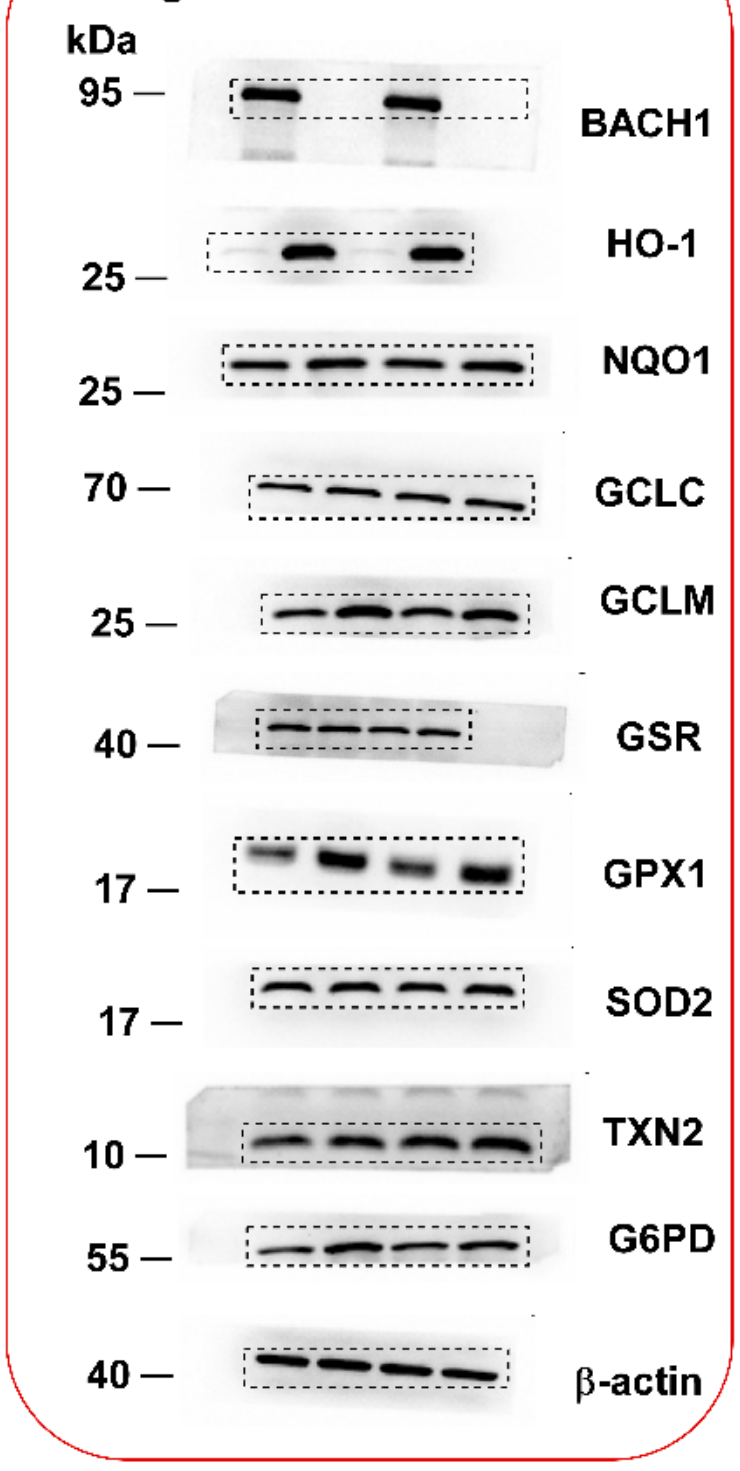

Figure S1K

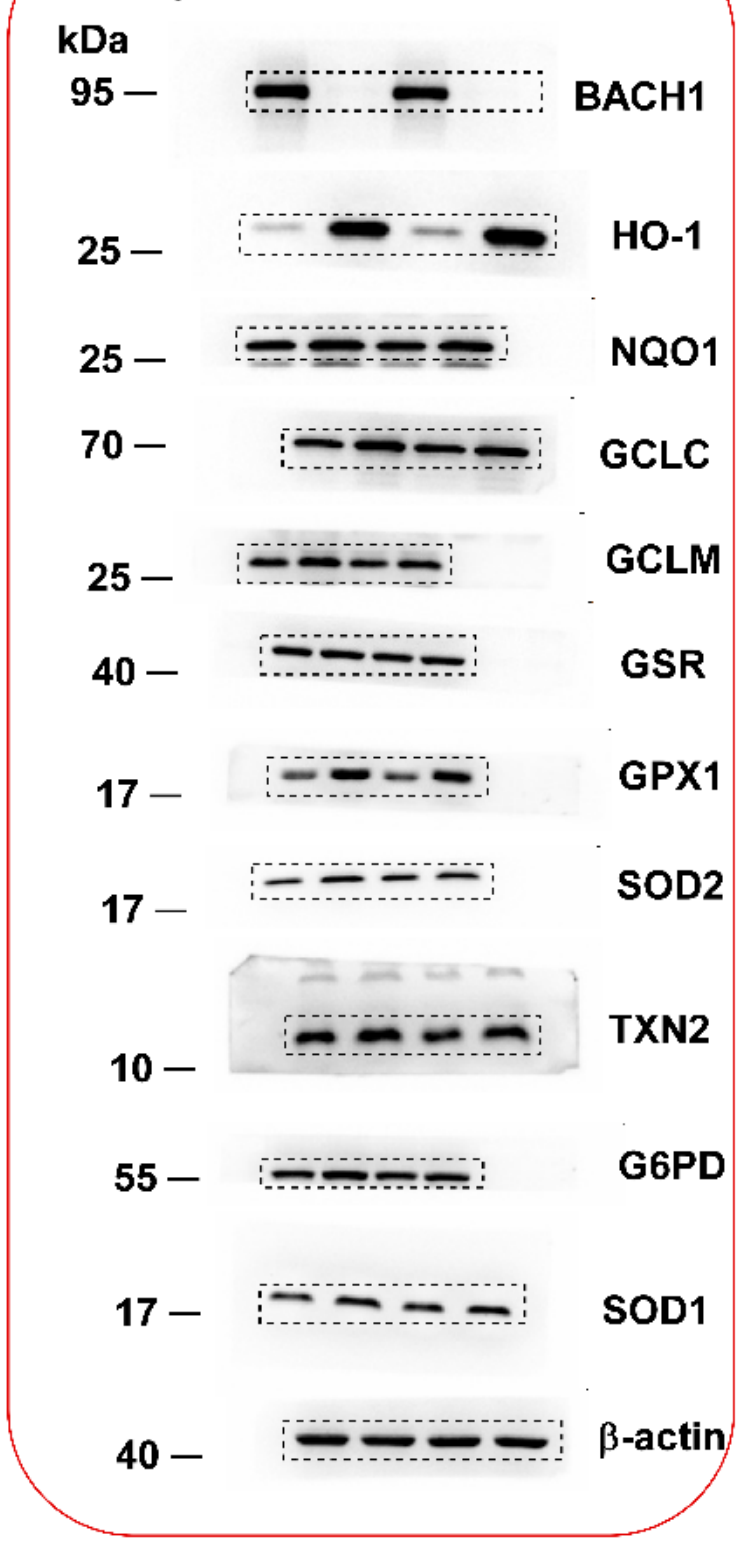

Figure S1L

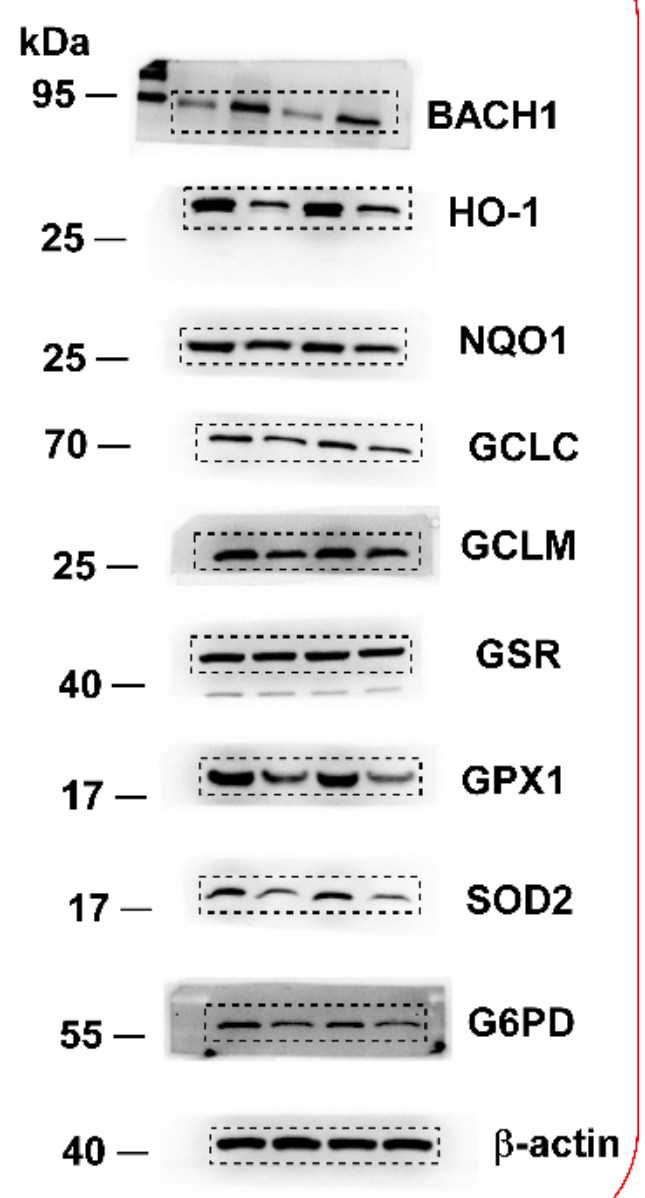

Figure S1O

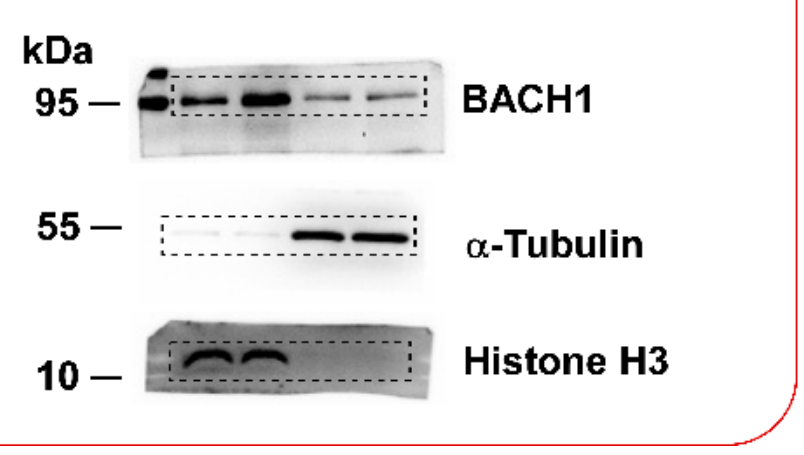

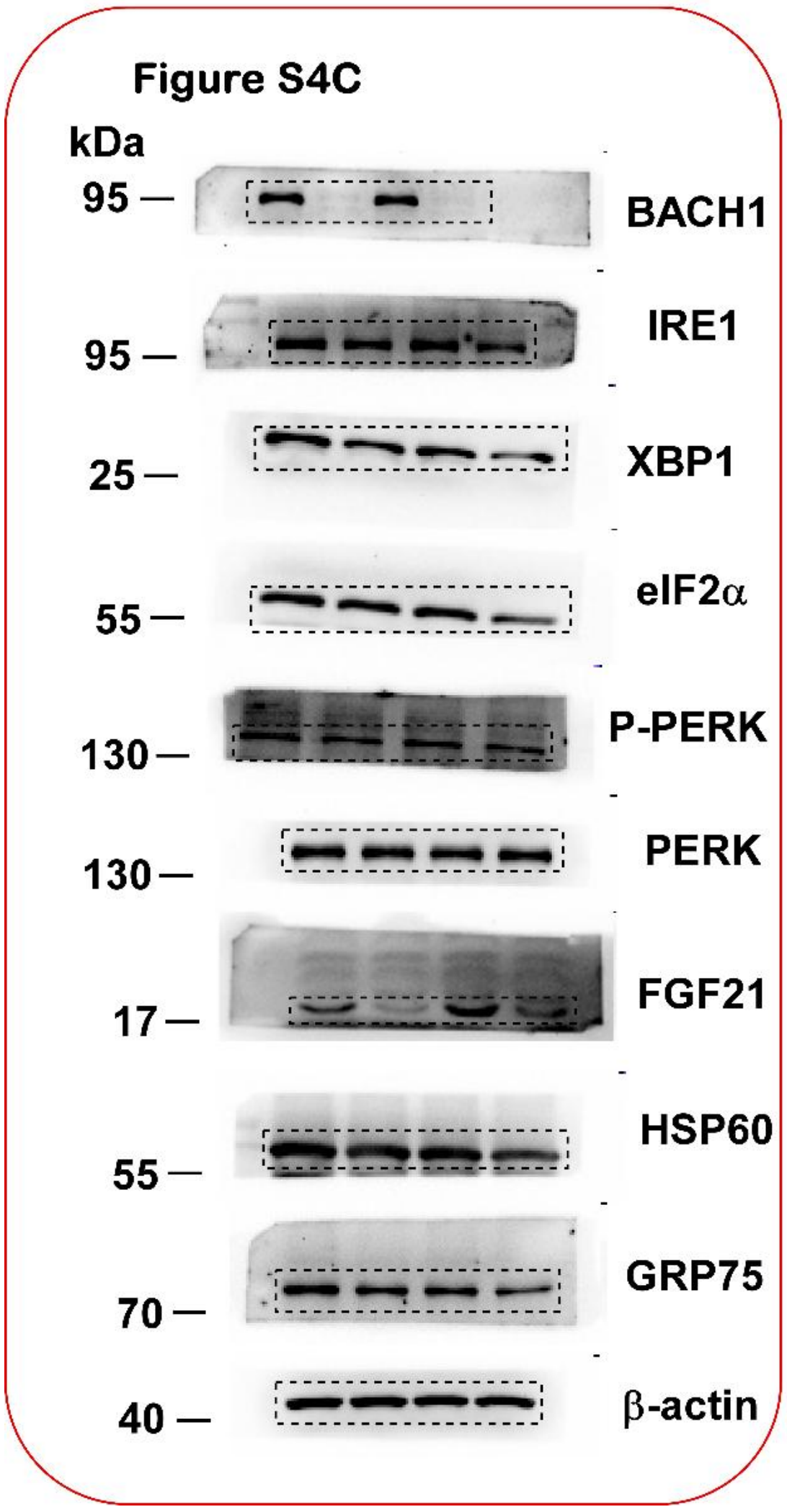
Figure S4C
kDa
95
BACH1
95
IRE1
25
XBP1
55
eIF2α
130
P-PERK
130
PERK
17
FGF21
55
HSP60
70
GRP75
40
β-actin

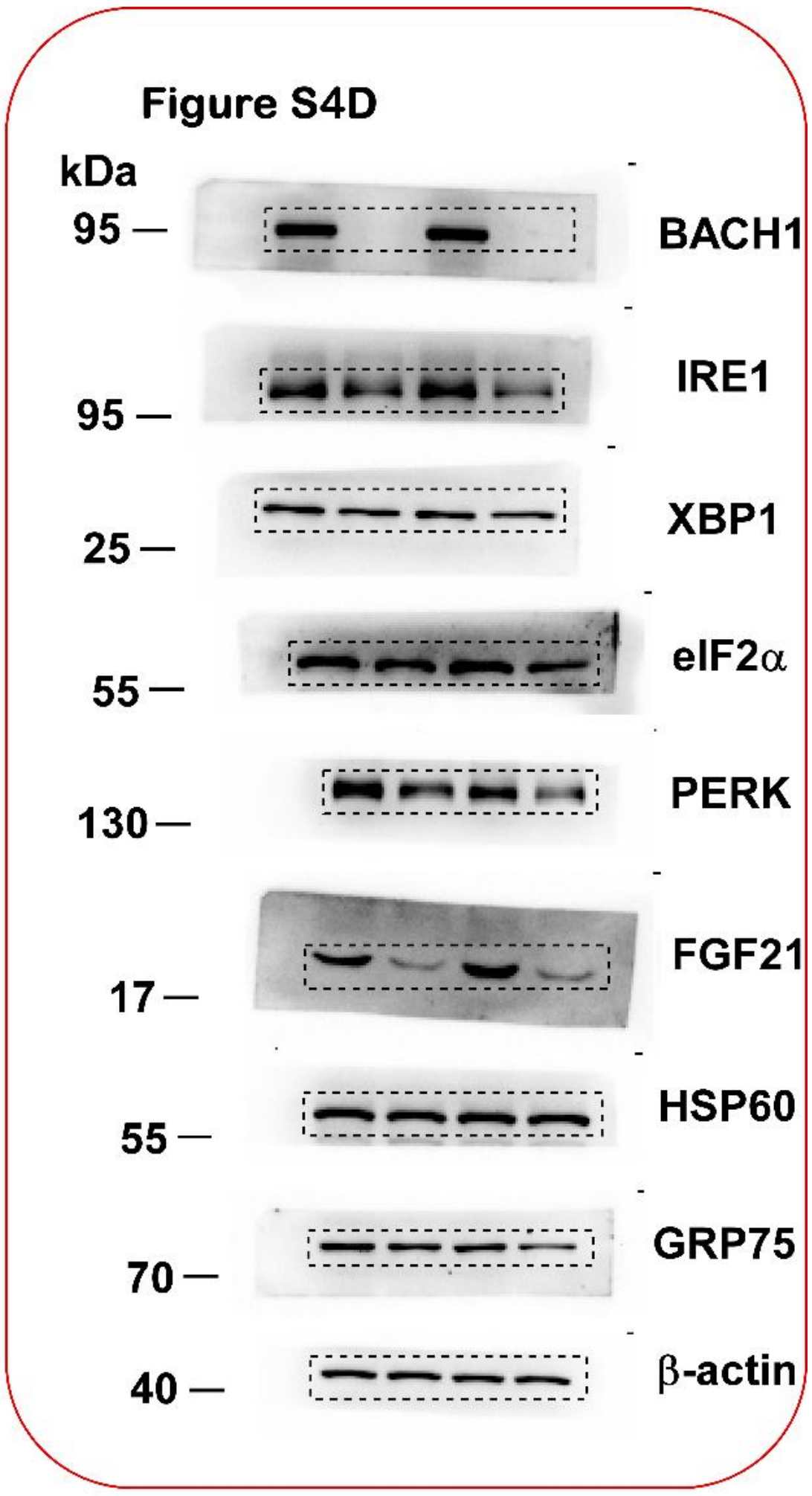
Figure S4D
kDa
95
BACH1
95
IRE1
25
XBP1
55
eIF2α
130
PERK
17
FGF21
55
HSP60
70
GRP75
40
β-actin

Figure S6A

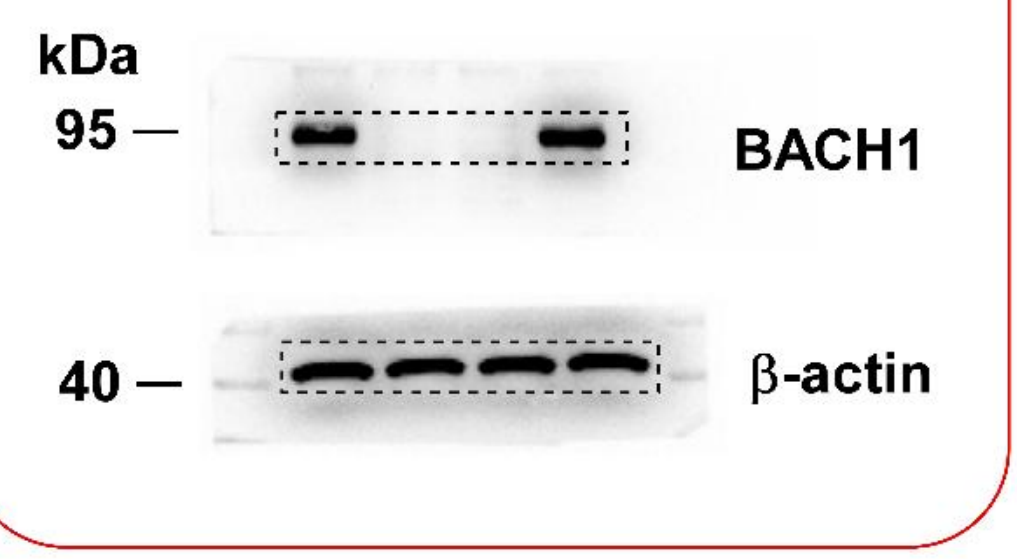

Figure S6B

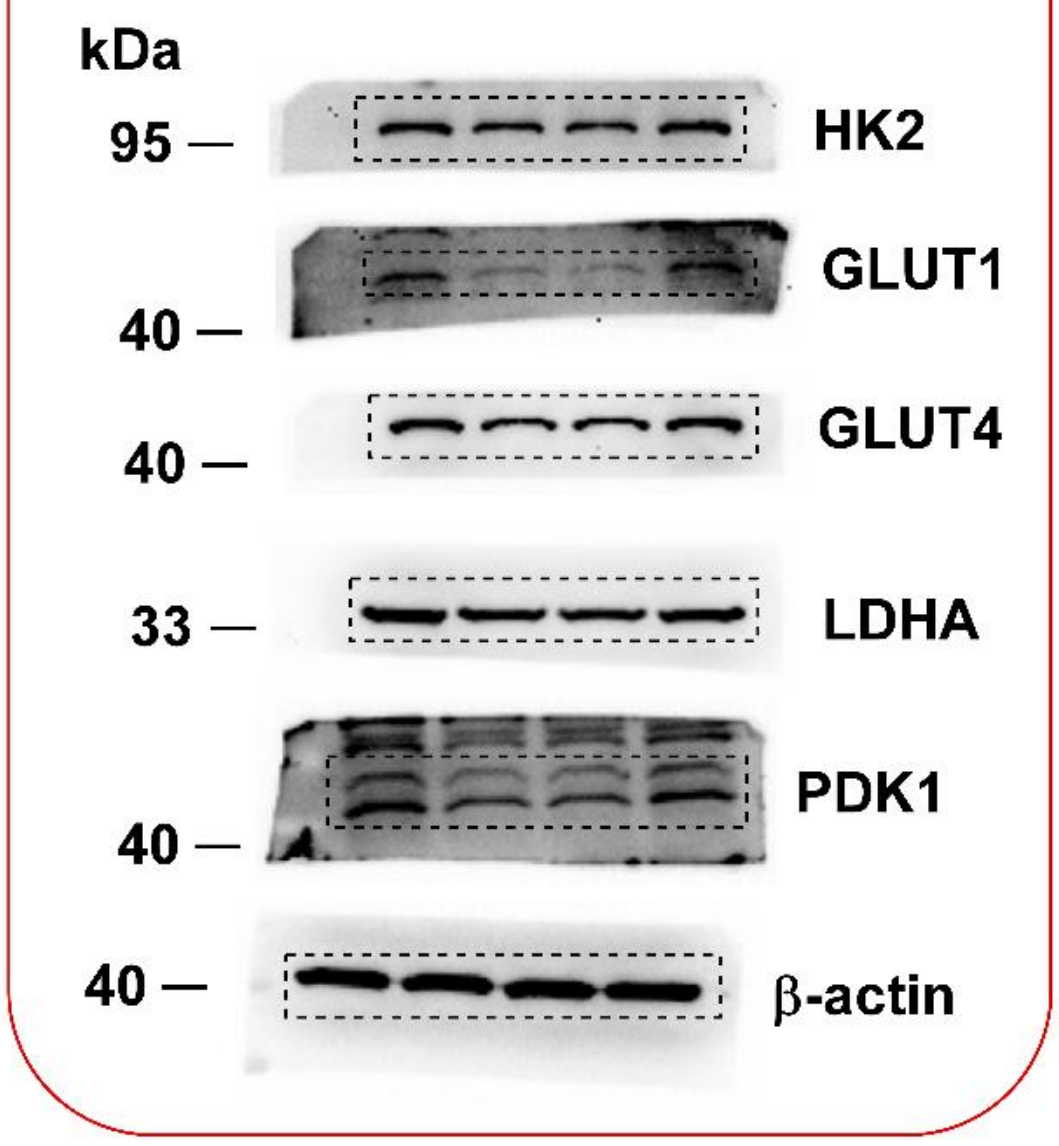

Figure S6C

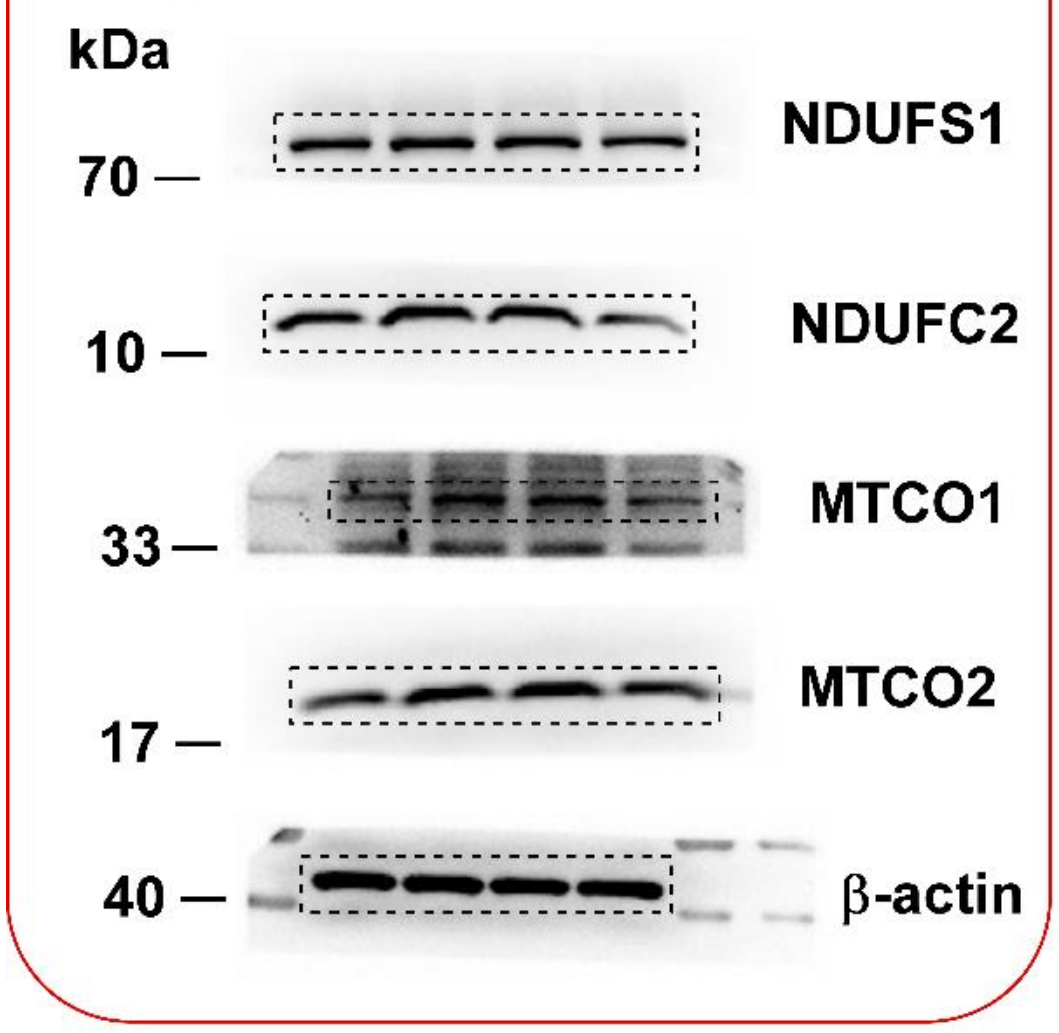

Figure S6D

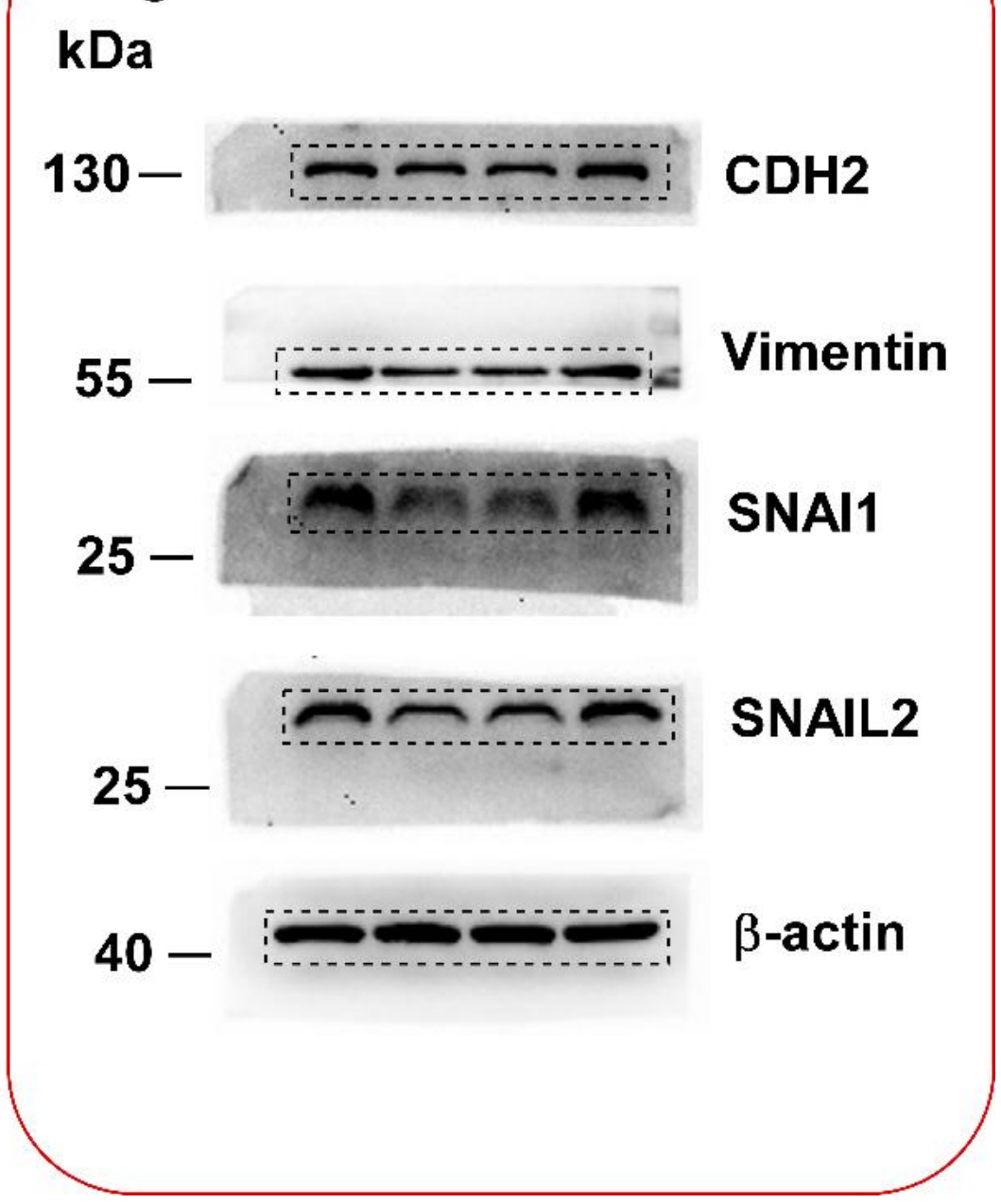

Supplement: Supplementary file 2 [file 41419_2026_8865_MOESM2_ESM.pdf]
